# Design, synthesis and potent anti-pancreatic cancer activity of new pyrazole derivatives bearing chalcone, thiazole and thiadiazole moieties: gene expression, DNA fragmentation, cell cycle arrest and SAR†

**DOI:** 10.1039/d4ra03005b

**Published:** 2024-08-27

**Authors:** Monica G. Kamel, Farid M. Sroor, Mahmoud KH. Hanafy, Karima F. Mahrous, Hamdi M. Hassaneen

**Affiliations:** a Department of Chemistry, Faculty of Science, Cairo University Giza Egypt hamdi_251@yahoo.com; b Organometallic and Organometalloid Chemistry Department, National Research Centre Cairo 12622 Egypt faridsroor@gmx.de fm.sroor@nrc.sci.eg; c Bioassay-Cell Culture Laboratory, National Research Centre Dokki 12622 Egypt; d Cell Biology Department, National Research Centre Dokki 12622 Egypt

## Abstract

Less than 5% of pancreatic cancer patients survive for more than five years after diagnosis. Therefore, there is an urgent need for novel therapeutic drugs to treat pancreatic cancer. Herein, we report the synthesis and full characterization of fifteen novel pyrazole derivatives bearing chalcone (4–10), thiazole (16–19) and thiadiazole (23–26) moieties. All the newly synthesized pyrazole derivatives were tested *in vitro* as anti-cancer agents against pancreatic cancer (PaCa-2), breast cancer (MCF-7), prostate cancer (PC3), and normal cell lines (BJ1). Three pyrazolyl-chalcone derivatives (4, 5, and 7) and a pyrazolyl-thiadiazole derivative (25) showed potent anti-cancer activity against the PaCa-2 cell line with IC_50_ values of 13.0, 31.5, 24.9, and 5.5 μg mL^−1^, respectively, compared with doxorubicin (IC_50_ = 28.3 μg mL^−1^). Compound 25 showed potent anti-cancer activity against the PC3 cell line with an IC_50_ value of 11.8 μg mL^−1^. In contrast, compounds 4, 5 and 7 are safer against the normal human-cell line (BJ1) with IC_50_ values of 74.2, 76.6 and 81.1 μg mL^−1^, respectively, compared with compound 25, which has an IC_50_ value of 23.7 μg mL^−1^. The mechanism of action of compounds 4, 5 and 7 against pancreatic cancer cells was studied by investigating gene expression, DNA fragmentation, comet assay and flow cytometry experiments using doxorubicin as a reference drug. Moreover, the structure–activity relationship between the structures of these compounds and their biological properties was discussed.

## Abstract 2

Four pyrazol derivatives (4, 5, 7, and 25) showed potent anti-PACA-2 cell line with IC_50_ (13.0, 31.5 and 24.9, 5.5 μg mL^−1^) respectively, while compounds 23 and 25 showed potent anti-PC3 cell line with IC_50_ (26.1 and 11.8 μg mL^−1^).

## Graphical abstract

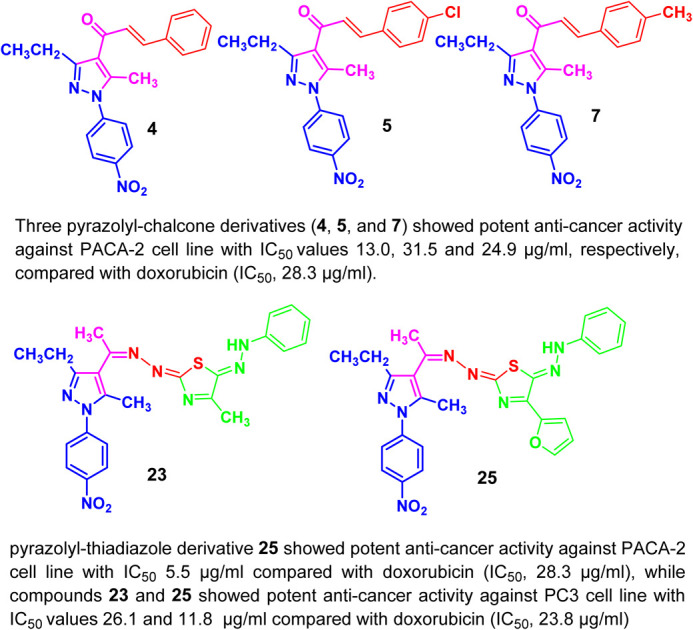

## 1. Introduction

Uncontrolled cell growth can generate cancer cells, which is a multifactorial disease and kills millions of people each year worldwide. Cancer is the cause of one in six deaths. In 2018, 18.1 million people were diagnosed with cancer globally, and it is expected that this number will increase to 29.4 million in 2040.^1^ Cancer treatments depend on a range of techniques, such as surgery, radiation, and chemotherapy, which can be used separately or together.^1,2^ Side effects and multidrug resistance (MDR) are the main obstacles in cancer therapy. Similarly, pancreatic cancer, also known as pancreatic adenocarcinoma (PDAC), is one of the most aggressive types of tumors in the world and is reported as the primary cause of cancer-related deaths.^3,4^ Moreover, less than 5% of pancreatic cancer patients have a survival rate exceeding five years.^5–7^ Therefore, there is an urgent need for novel therapeutic drugs to treat pancreatic cancer.

**Fig. 1:**
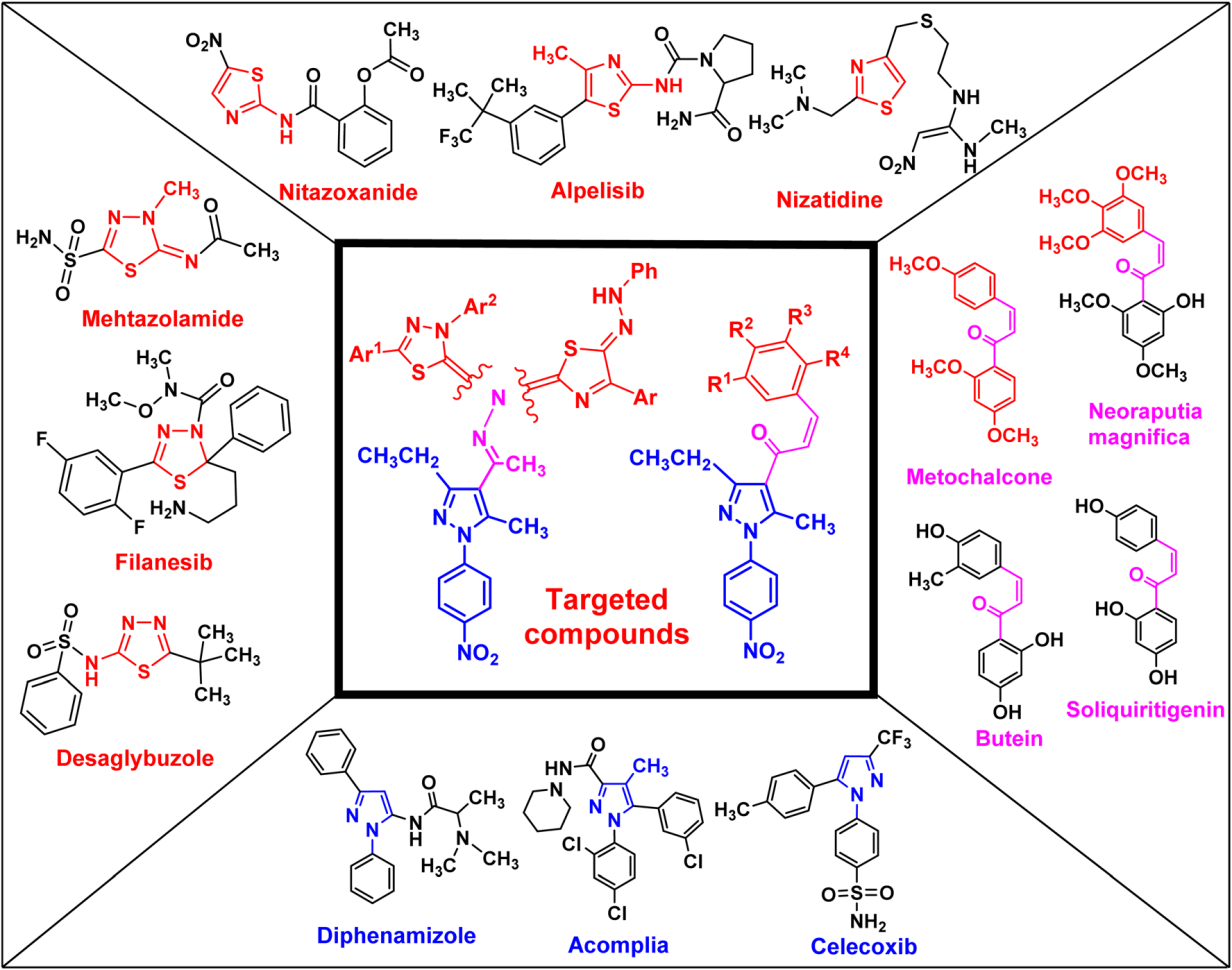
Rationale design of the targeted pyrazole derivatives using the molecular hybridization strategy.

Chalcone and its derivatives, which have an α,β-unsaturated system with three carbons, exhibit diverse biological properties and a preferred synthon to generate different kinds of heterocyclic rings.^8–11^ Chalcones are a member of the flavonoid family; moreover, the chemical structure of chalcone has been reported in many natural products, including vegetables, teas, fruits, and spices.^12,13^ Furthermore, they are used as anti-inflammatory,^14^ antiobesity,^15^ thioredoxin reductase inhibitory,^16^ antiprotozoal,^17^ antiplatelet,^18^ tubulin polymerization inhibitory,^19^ antidiabetic,^20^ antitubercular,^21^ antibacterial,^8,22^ anti-Alzheimer,^23^ and antimalarial agents^24^ (some biologically active chalcone derivatives are given in Fig. 1).

Heterocyclic compounds are an essential and unique class of compounds that cover a wide range of reactivity and stability and have a broad spectrum of chemical and biological characteristics.^25–29^ Heterocycle-based medications are adaptable enough to target a wide range of metabolic pathways and cellular processes involved in the development and progression of cancer.^30–33^ Therefore, numerous heterocyclic derivatives have been designed and synthesized for their possible uses as anti-cancer agents. Among these structures, the five-membered heterocyclic rings, such as pyrazole, thiazole, and thiadiazoles, are particularly important compounds.^34–37^ The pyrazole ring, as a five-membered heterocycle with two neighboring nitrogen atoms, is present in a wide range of compounds that possess different applications. Furthermore, it is commonly known that pyrazoles, both naturally occurring and synthesized, have a wide range of biological properties (some biologically active pyrazoles are given in Fig. 1).^38^ The thiazole moiety is a significant aromatic five-membered heterocycle. Its distinctive biological properties are established by the atoms of sulfur and nitrogen and the thiazole scaffold is found in over eighteen FDA-approved medications.^39^ It has been indicated that thiazole-containing compounds demonstrate a variety of biological properties, such as antifungal,^40^ antibacterial,^41^ anticancer,^28^ diuretic,^42^ anti-inflammatory,^43^ analgesic,^44^ neuroprotective,^45^ antimalarial,^46^ and antioxidant^47^ (some biologically active thiazoles are given in Fig. 1). The thiadiazole scaffold can be of four different types: 1,2,3-, 1,2,4-, 1,2,5- and 1,3,4-thiadiazole.^48–50^ The remarkable pharmacological characteristics of 1,3,4-thiadiazole derivatives are ascribed to their stability, high aromaticity, and absence of toxicity.^51^ Furthermore, due to the hydrogen binding domain of the 1,3,4-thiadiazole ring, it can be used as a potential agent in several FDA-approved medications, including methazolamide, sulfamethizole, desaglybuzole, litronesib, and filanesib (Fig. 1).^34^

The design and development of heterocyclic small molecules as anti-cancer agents has been an active area of research in recent years. Several studies have reported the synthesis and biological evaluation of pyrazole-containing compounds as potential therapeutics for various tumor types.^52^ For instance, Dabhade *et al.* described the preparation of novel pyrazole-chalcone hybrids and demonstrated their cytotoxic effects against a panel of breast cancer cell lines.^53^ Similarly, Yadav *et al.* developed pyrazole-based derivatives bearing thiazole moieties and found them to be promising anti-gastric agents.^54^ However, the incorporation of multiple heterocyclic substructures, such as chalcone, thiazole, and thiadiazole, within a single pyrazole scaffold as anti-pancreatic agents has been less explored. The present study builds upon these earlier efforts and presents a novel class of pyrazole derivatives that display enhanced anti-pancreatic cancer potency compared to previously reported pyrazole-based compounds.^55^ The synergistic effects arising from the combination of these heterocyclic fragments represent a unique structural design approach that sets this work apart from the state-of-the-art in this research area. The detailed structure–activity relationship analysis provided in this study further contributes to the growing body of knowledge on the development of heterocyclic small molecules as promising anti-pancreatic drug candidates.

In this study, we used the molecular hybridization strategy, a frequently utilized technique in the field of drug discovery, to synthesize effective pyrazole derivatives bearing chalcone, thiazole or thiadiazole moieties (Fig. 1). Fifteen novel hybrid pyrazole derivatives were designed and tested as anti-cancer agents against selected human cancerous cell lines of breast (MCF-7), pancreatic (PaCa-2), and prostate (PC3) relative to healthy noncancerous control skin fibroblast cells (BJ-1). Moreover, the mechanism of action of the most active compounds was investigated by studying the DNA fragmentation and gene expression as well as the structure–activity relationship (SAR).

## 2. Results and discussion

### 2.1. Chemistry

**Scheme 1:**
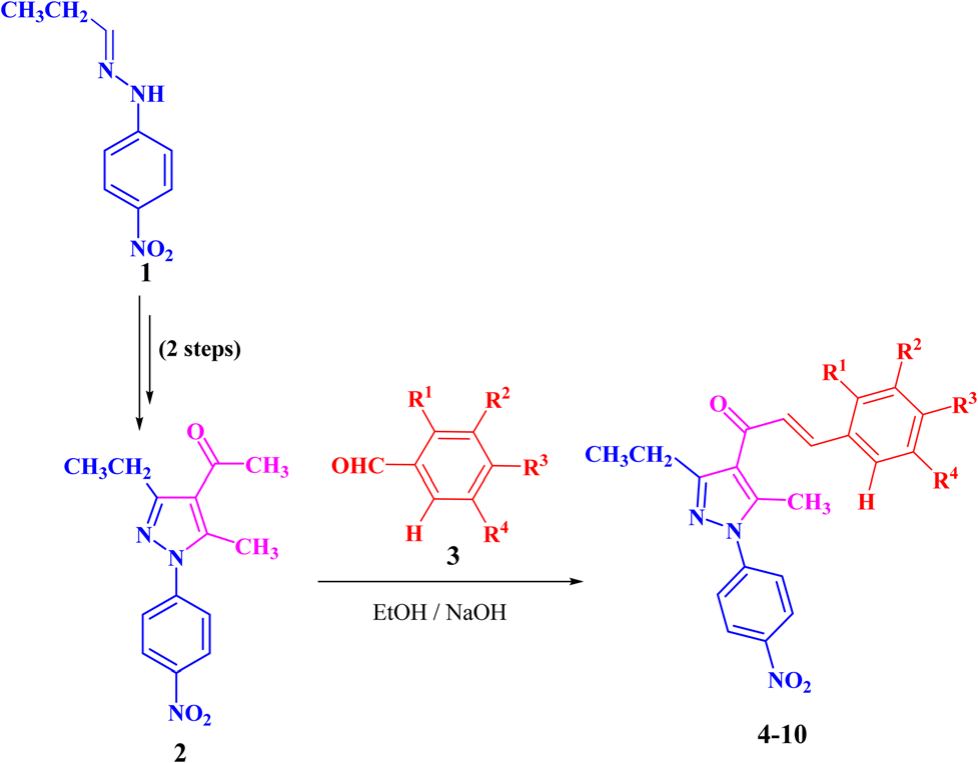
Synthesis of pyrazolyl-chalcone derivatives 4–10.

**Fig. 2:**
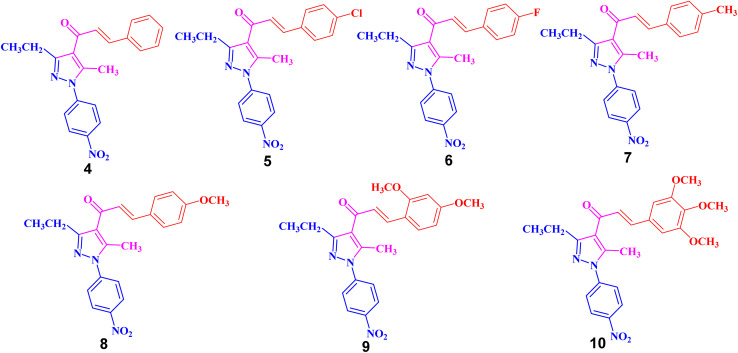
Chemical structures of pyrazolyl-chalcone derivatives 4–10.

The targeted precursor of 2 was prepared in two steps as described in the literature.^9,56^ The bromination of propionaldehyde-4-nitrophenylhydrazone followed by the reaction of the produced *N*-(4-nitrophenyl)propionohydrazonoyl bromide with acetylacetone in the presence of sodium ethoxide afforded 1-(3-ethyl-5-methyl-1-(4-nitrophenyl)-1*H*-pyrazol-4-yl)ethan-1-one (2) in good yields. Claisen–Schmidt condensation of compound 2 with equimolar amounts of aryl-aldehyde derivatives (3) in ethanol and in the presence of sodium hydroxide solution afforded the targeted pyrazolyl-chalcone derivatives (4–10) (Scheme 1 and Fig. 2).

The structures of the formed pyrazolyl-chalcone derivatives 4–10 (Fig. 2) were confirmed from their spectral data, such as ^1^H NMR, ^13^C NMR, IR, MS, and elemental analysis. For instance, the ^1^H NMR spectrum of pyrazolyl-chalcone 4 as a representative example showed the following signals: a triplet at *δ* 1.35 (ppm) corresponding to the CH_2_C*H*_3_ group and a singlet signal at *δ* 2.62 (ppm) corresponding to the CH_3_ group. The methylene group (C*H*_2_CH_3_) was assigned as a quartet at *δ* 2.98 (ppm), while the aromatic protons were observed as a multiplet at *δ* 7.20–7.73 (ppm) and a doublet at *δ* 8.40 (ppm). The ^13^C NMR spectrum of 4 revealed 17 signals corresponding to asymmetric carbon atoms. Also, the mass spectrum (EI) of 4 showed a molecular ion peak at *m*/*z* = 361. In addition, the IR spectrum of 4 showed the band at 1654 (cm^−1^) corresponding to the conjugated C=O group.

**Scheme 2:**
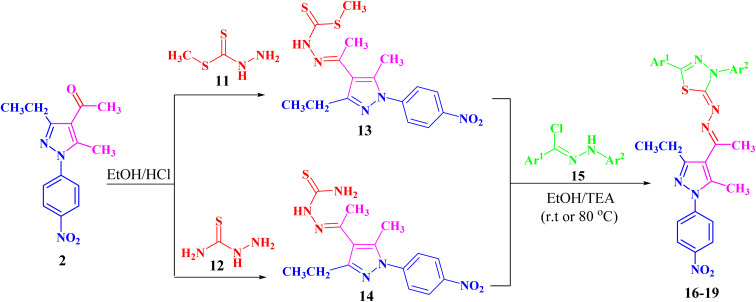
Synthesis of the targeted pyrazolyl-thiadiazole derivatives 16–19 in two different ways.

Refluxing of 1-(3-ethyl-5-methyl-1-(4-nitrophenyl)-1*H*-pyrazol-4-yl)-ethan-1-one (2) with methyl hydrazinecarbodithioate (11)^57,58^ in absolute ethanol in the presence of a few drops of hydrochloric acid afforded methyl 2-(1-(3-ethyl-5-methyl-1-(4-nitrophenyl)-1*H*-pyrazol-4-yl)ethyl-idene)hydrazine-1-carbodithioate (13) (Scheme 2). The structure of compound 13 was elucidated from spectral data (^1^H NMR, ^13^C NMR, IR and MS) and elemental analysis. The ^1^H NMR spectrum of 13 revealed the following signals: a triplet at *δ* 1.34 (ppm) corresponding to the CH_2_C*H*_3_ group, four singlets at *δ* 2.35, 2.52, 2.61 and 10.03 (ppm), which could be assigned to three CH_3_ groups and NH group, respectively. A quartet signal of the methylene group (C*H*_2_CH_3_) was observed at *δ* 2.94 (ppm) and two doublets at *δ* 7.73 and 8.39 (ppm) corresponding to the protons of 4-NO_2_C_6_H_4_. The ^13^C NMR spectrum showed 14 signals corresponding to asymmetric carbon atoms. Also, the mass spectrum (EI) of 13 showed a molecular ion peak at *m*/*z* = 377. In addition, its IR spectrum showed a band at 3179 (cm^−1^), which could be attributed to the NH group.

Likewise, 2-(1-(3-ethyl-5-methyl-1-(4-nitrophenyl)-1*H*-pyrazol-4-yl)ethylidene)hydrazine-1-carbothioamide (14) was prepared *via* the treatment of acetylpyrazole 2 with hydrazinecarbothioamide 12 under the same conditions (Scheme 2). The structure of compound 14 was elucidated from spectral data (^1^H NMR, ^13^C NMR, IR and MS) and elemental analysis. The ^1^H NMR spectrum of compound 14 demonstrated the following signals: a triplet at *δ* 1.18 (ppm) corresponding to the methyl group (CH_2_C*H*_3_) and five singlets at *δ* 2.29, 2.44, 7.48, 8.23 and 10.16 (ppm) corresponding to two CH_3,_ NH_2_ and NH groups, respectively. A quartet signal at *δ* 2.73 (ppm) refers to the methylene group (C*H*_2_CH_3_) and two doublets at *δ* 7.83 and 8.38 (ppm) correspond to the protons of 4-NO_2_C_6_H_4_. Its ^13^C NMR spectrum showed 13 signals corresponding to asymmetric carbon atoms. Also, the mass spectrum (EI) of 14 showed a molecular ion peak at *m*/*z* = 346. Its IR spectrum showed bands at 3464 cm^−1^ corresponding to the NH group and 3225 and 3298 (cm^−1^) referring to the NH_2_ group.

**Fig. 3:**
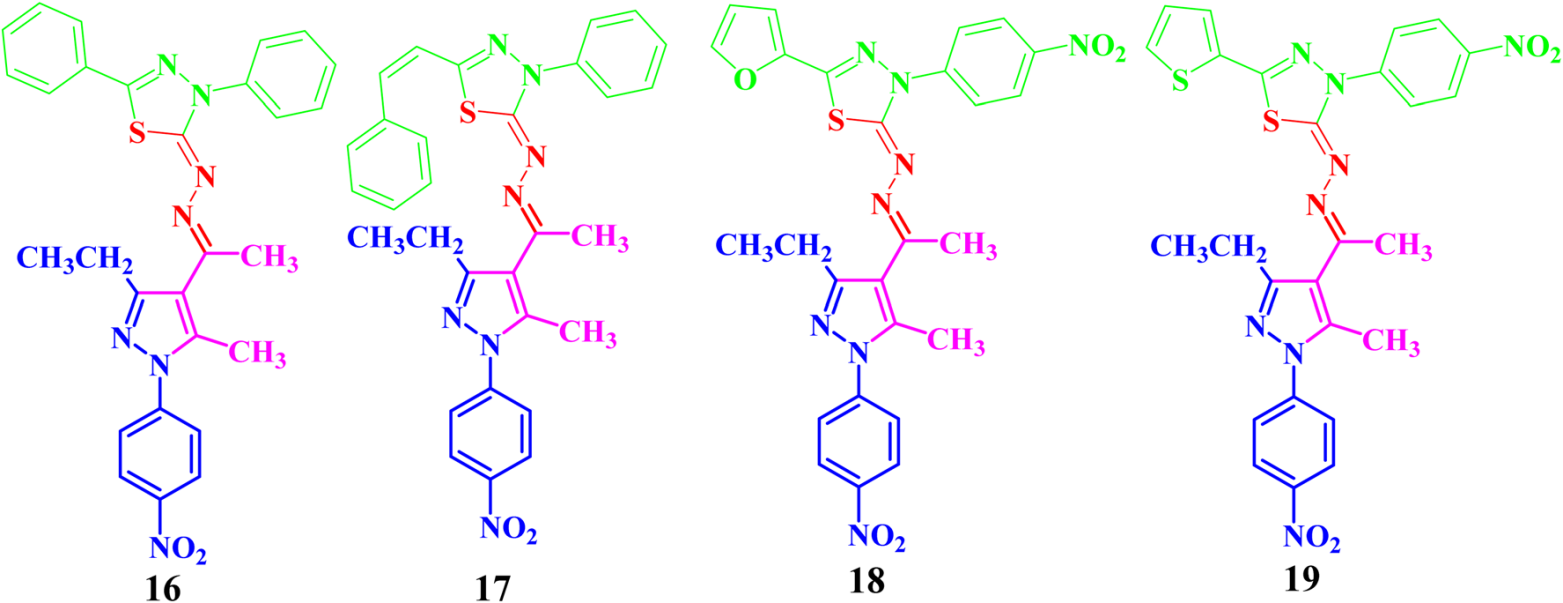
Chemical structures of the targeted pyrazolyl-thiadiazole derivatives 16–19.

Both compounds 13 and 14 were used as suitable precursors to synthesize the targeted pyrazolyl-thiadiazole derivatives 16–19, as depicted in Scheme 2. Compound 13 was treated with appropriate hydrazonoyl chlorides (15) in absolute ethanol and in the presence of trimethylamine as a catalyst at ambient temperature to afford the corresponding pyrazolyl-thiadiazole derivatives 16–19 (Scheme 2 and Fig. 3). Compounds 16–19 could be obtained in different routes *via* the reaction of 14 with hydrazonoyl chlorides (15) at 80 °C in absolute ethanol and in the presence of trimethylamine (Scheme 2). Structures of pyrazolyl-thiadiazole derivatives 16–19 (Fig. 3) were elucidated from their spectral data (^1^H NMR, ^13^C NMR, IR and MS) and elemental analysis. For example, the ^1^H NMR spectrum of 16 (Fig. 3) showed the following signals: a triplet at *δ* 1.35 (ppm) corresponding to the methyl group (CH_2_C*H*_3_), two singlet at *δ* 2.51 and 2.60 (ppm) corresponding to the other methyl groups, and a quartet at *δ* 2.92 (ppm) corresponding to the methylene group (C*H*_2_CH_3_). The aromatic protons were assigned as a multiplet at the range *δ* 7.27–7.83 (ppm) corresponding to 10 protons and two doublets at *δ* 8.26 and 8.38 (ppm) belonging to 4 protons of NO_2_C_6_H_4_. Its ^13^C NMR spectrum showed 22 signals corresponding to asymmetric carbon atoms. In addition, the IR spectrum of 16 revealed the absence of both the NH and NH_2_ bands.

**Scheme 3:**
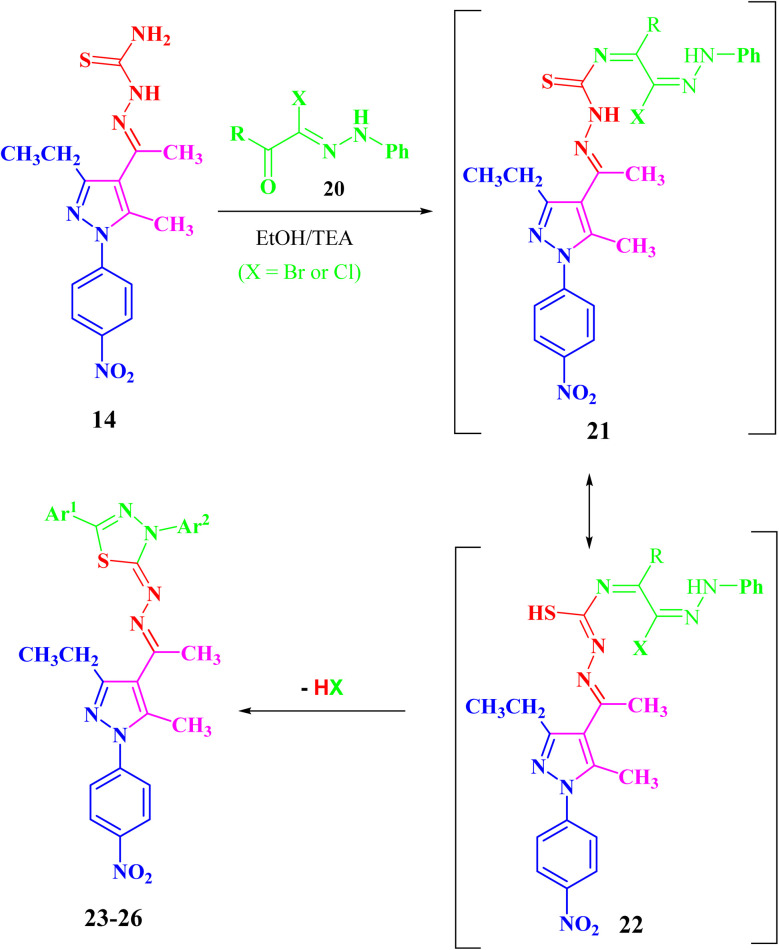
Synthesis of the targeted pyrazolyl-thiazole derivatives 23–26.

**Fig. 4:**
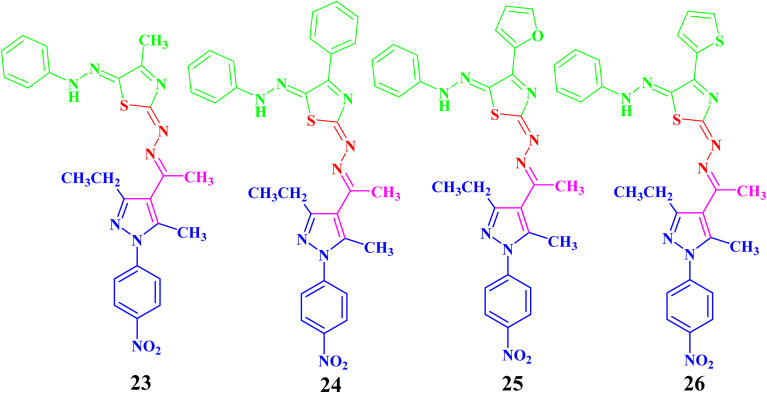
Chemical structures of the targeted pyrazolyl-thiazole derivatives 23–26.

In the same manner, the targeted pyrazolyl-thiazole derivatives 23–26 (Scheme 3 and Fig. 4) were prepared using methyl 2-(1-(3-ethyl-5-methyl-1-(4-nitrophenyl)-1*H*-pyrazol-4-yl)ethylidene)hydrazine-1-carbothioamide (14) as a suitable precursor by reaction with appropriate α-ketohydrazonoyl halides (20) in absolute ethanol and the presence of trimethylamine. The reaction involved condensation *via* elimination of water molecule to give the expected intermediate (21), which converted as soon as it was formed to the other intermediate (22), which gave the final products 23–26*via* elimination of hydrogen halide (Scheme 3). Elemental analyses and spectral data (^1^H NMR, ^13^C NMR, IR and MS) verified the chemical structures of the synthesized pyrazolyl-thiazole derivatives 23–26 (Fig. 4).

Compound 23 will be discussed in more detail as a representative example for a series of pyrazolyl-thiazole (23–26). In the ^1^H NMR spectrum of 23 (Fig. 4), a triplet signal at *δ* 1.34 (ppm) is due to the methyl group (CH_2_C*H*_3_), three singlet signals at *δ* 2.01, 2.61 and 2.62 (ppm) refer to the other methyl groups, and a quartet signal at *δ* 2.97 (ppm) for the methylene group (C*H*_2_CH_3_). The aromatic protons were assigned as a multiplet at range *δ* 7.03–7.37 (ppm) due to 5 protons and two doublets at *δ* 7.72 and 8.39 (ppm) belonging to 4-NO_2_C_6_H_4_, while the proton of the NH group was observed at *δ* 7.49 (ppm). The ^13^C NMR spectrum showed 20 signals corresponding to asymmetric carbon atoms. The IR spectrum revealed the presence of a band at 3427 (cm^−1^), referring to the NH group. Also, the mass spectrum (EI) of 23 depicted a molecular ion peak at *m*/*z* = 489.

### 2.2. Anti-cancer activity

#### 2.2.1. Primary screening

The newly synthesized pyrazolyl-chalcone (4–10), pyrazolyl-thiadiazole (16–19) and pyrazolyl-thiazole (23–25) derivatives were screened *in vitro* against pancreatic cancer (PaCa-2), breast cancer (MCF-7) and prostate cancer (PC3) cell lines as well as normal cell lines (BJ1). The findings showed that the majority of these compounds had a potent inhibition effect on PaCa2 and MCF-7 cell lines, while compounds 23 and 25 showed remarkable mortality against the PC3 cell line with (92.8%) and (100%), respectively (Table 1). As summarized in Table 1, compounds 4–10 of pyrazolyl-chalcone derivatives exhibited potent anti-cancer mortality, as shown for compounds 4 (100%), 5 (88.3%), 6 (51.2%), 7 (91.2%), 8 (90.5%), 9 (53.8%) and 10 (63.4%) on PaCa-2 cell line, while only compound 16 from pyrazolyl-thiadiazole series showed 74.6% mortality. Compounds 23 and 25 from the pyrazolyl-thiazole series showed 94.2% and 100% mortality, respectively, on the PaCa-2 cell line. On the MCF-7 cell line, pyrazolyl-chalcone derivatives exhibited medium mortality for compounds 5 (61.2%), 7 (65.3%), 8 (86.5%), and 10 (68.3%) compared with pyrazolyl-thiadiazoles in compounds 16 (74.9%) and 17 (71.2%) and pyrazolyl-thiazoles in compounds 23 (100%) and 24 (73.9%). Finally, compounds 4, 5 and 7 showed high safety against the human normal cell line (BJ1) with limited % mortality of 61%, 62% and 45%, respectively, compared with compounds 8 (74%), 23 (83%) and 25 (100%). Due to these results, compounds 4, 5, and 7 underwent secondary screening to determine their selectivity index and IC_50_ values.

**Table 1:** (%) mortality of cancer and normal cell lines at 100 μg mL^−1^

Comp.	PaCa2	MCF7	PC3	BJ1
4	100	31.2 ± 0.77	5.3 ± 0.87	61.2 ± 0.44
5	88.3 ± 0.11	61.2 ± 0.94	5.7 ± 0.21	61.8 ± 0.27
6	51.2 ± 0.23	28.6 ± 0.16	8.4 ± 0.87	
7	91.2 ± 0.41	65.3 ± 0.19	31.5 ± 0.70	44.9 ± 0.62
8	90.5 ± 0.57	86.5 ± 0.32	30.7 ± 0.19	74.6 ± 0.28
9	53.8 ± 0.27	16.3 ± 0.52	27.6 ± 0.14	
10	63.4 ± 0.31	68.3 ± 0.63	48.6 ± 0.55	
16	74.6 ± 0.22	74.9 ± 0.87	1.5 ± 0.65	
17	45.6 ± 0.84	71.2 ± 0.14	51.2 ± 0.70	
18	46.3 ± 0.42	51.3 ± 0.18	40.8 ± 0.74	
19	5.7 ± 0.73	28.6 ± 0.79	4.2 ± 0.45	
23	94.2 ± 0.44	100	92.8 ± 0.21	83.5 ± 0.32
24	62.4 ± 0.25	73.9 ± 0.56	56.3 ± 0.29	
25	100	100	100	100
DOX	100	100	100	100

#### 2.2.2. Secondary screening (IC_50_ determination)

As depicted in Table 2, three pyrazolyl-chalcone derivatives (4, 5 and 7) as well as pyrazolyl-thiadiazole derivative (25) have a potent anti-cancer activity against PaCa-2 cell line with IC_50_ values of 13.0, 31.5, 24.9, and 5.5 μg mL^−1^, respectively, compared with the positive reference drug Doxorubicin (Dox) with IC_50_ (28.3 μg mL^−1^). Compound 23 exhibited promising anti-cancer properties against MCF-7 and PC3 cell lines with IC_50_ values of 29.9 and 26.1 μg mL^−1^ compared with the positive control (IC_50_ 26.1 and 23.8 μg mL^−1^), while compound 25 showed potent anti-cancer activity against PC3 cell lines with IC_50_ value of 11.8 μg mL^−1^. On the other hand, compounds 4, 5 and 7 are safer against the human normal cell line (BJ1) with IC_50_ values of 74.2, 76.6 and 81.1 μg mL^−1^ compared with compounds 23 and 25, which have IC_50_ values of 42.9 and 23.7 μg mL^−1^. Consequently, compounds 4, 5 and 7 were selected to investigate their molecular properties on the PaCa-2 cell line by studying their mechanism of action using DNA fragmentation and gene expression experiments.

**Table 2:** IC_50_ (μg mL^−1^) for the most promising compounds

Comp.	PaCa2	MCF7	PC3	BJ1
4	13.0 ± 0.21	—	—	74.2 ± 0.11
5	31.5 ± 0.42	69.6 ± 0.72	—	76.6 ± 0.15
6	—	—	—	—
7	24.9 ± 0.15	78.3 ± 0.17	—	81.1 ± 0.13
8	33.9 ± 0.17	40.2 ± 0.10	—	44.9 ± 0.22
9	—	—	—	—
10	—	—	—	—
16	62.0 ± 0.35	54.8 ± 0.74	—	—
17	—	57.2 ± 0.54	83.7 ± 0.14	—
18	—	—	—	—
19	—	—	—	—
23	38.7 ± 0.78	29.9 ± 0.11	26.1 ± 0.65	42.9 ± 0.34
24	—	64.4 ± 0.18	82.1 ± 0.21	—
25	5.5 ± 0.22	—	11.8 ± 0.24	23.7 ± 0.41
DOX	28.3 ± 0.19	26.1 ± 0.1	23.8 ± 0.47	13.5 ± 0.52

#### 2.2.3. Measurement of DNA fragmentation

**Fig. 5:**
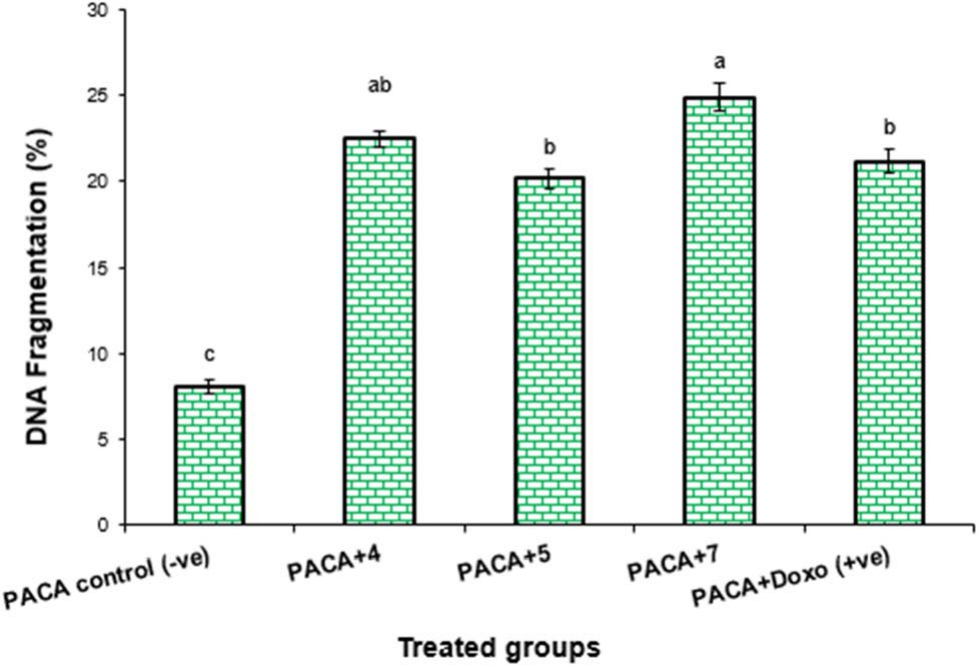
DNA fragmentation was detected in PaCa-2 cell lines treated with different compounds (4, 5 and 7) and Dox. Means with different superscripts (^a,b,c^) between locations in the same column are significantly different at *P* < 0.05.

**Fig. 6:**
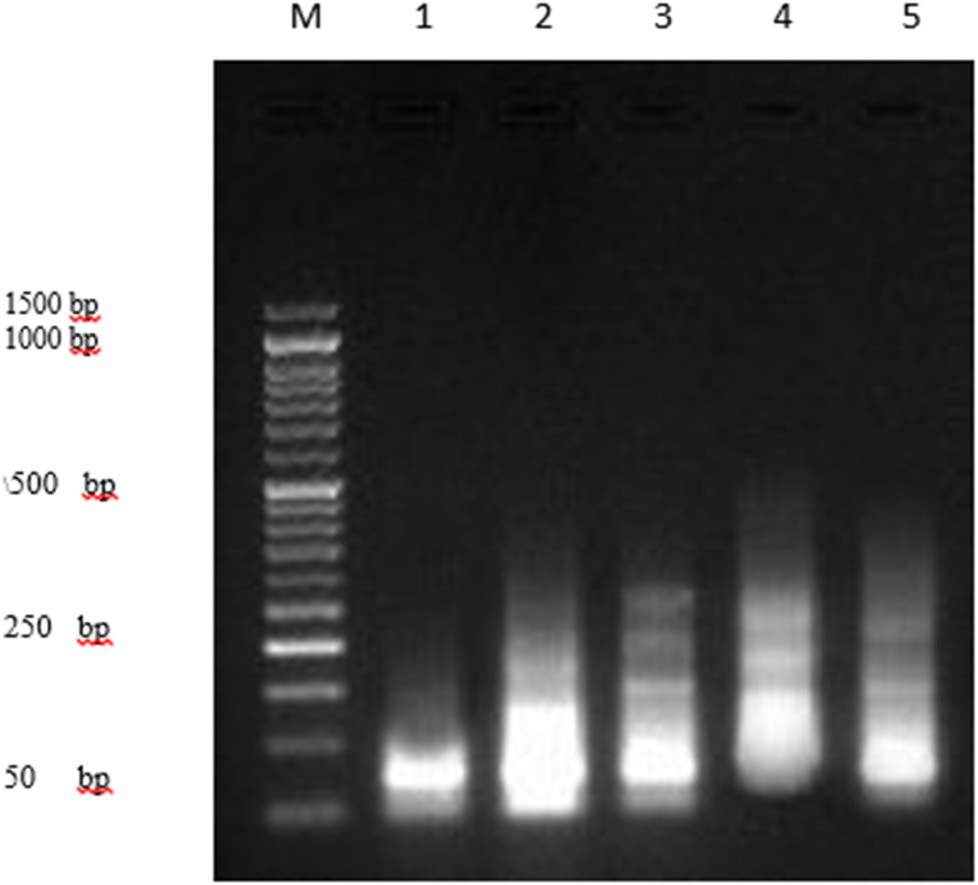
DNA fragmentation was detected with agarose gel in PaCa-2 cells treated with different compounds 4, 5 and 7 as well as Dox as the positive control. M: represent DNA marker, lane 1: represents PaCa-2 control (−ve), lane 2: represents PaCa + 4, lane 3: represents PaCa + 5, lane 4: represents PaCa + 7, lane 5: represents PaCa + Dox (+ve).

DNA fragmentation rate assessment in PaCa2 cell lines treated with compounds 4, 5 and 7 as well as the Dox drug is summarized in Table 3, Fig. 5 and 6. The results found that the negative control, namely PaCa-2 cell lines, showed a significant decrease (*P* < 0.01) in DNA fragmentation rates (8.1 ± 0.32) compared with those in positive control (21.2 ± 0.64). In contrast, the DNA fragmentation rates were increased significantly (*P* < 0.001) in treated PaCa-2 cell lines with compound 5 (24.9 ± 0.69) compared with negative control. Moreover, the DNA fragmentation increased in ascending manner from PaCa-2 negative control < PaCa-2 + 5 < PaCa + Dox (positive control) < PaCa-2 + 4 < PaCa + 7 treated cell lines (Table 3 and Fig. 5).

**Table 3:** DNA fragmentation was detected in PaCa-2 cell lines treated with compounds (4, 5 and 7) and Dox^a^

Treatment	DNA fragmentation (%) M ± SEM	Change	Inhibition (%)
PaCa-2 control (−ve)	8.1 ± 0.32^c^	0	0
PaCa-2 + 4	22.5 ± 0.44^ab^	14.4	23.21
PaCa-2 + 5	20.2 ± 0.53^b^	12.1	22.87
PaCa-2 + 7	24.9 ± 0.69^a^	16.8	28.24
PaCa-2 + Dox (+ve)	21.2 ± 0.64^b^	13.1	24.01

a Means with different superscripts (a, b, and c) between locations in the same column are significantly different at *P* < 0.05.

Pyrazole and its derivatives have been synthesized for a wide range of biological activities, including antimicrobial, antifungal, anti-inflammatory, anti-cancer, neuroprotective, and anti-viral activity.^59–61^ Certain Food and Drug Administration (FDA)-approved drugs, such as celecoxib, deracoxib, etoricoxib, and atorivodine, possess pyrazole scaffolds as the effective functional moiety.^62,63^ Furthermore, this study showed that compounds 4, 5 and 7 induce apoptosis-related genes (Caspase-3 and Caspase-8) and anticancer-related genes (CDK6) as well as increased DNA damage response. So, our data suggest that compounds 4, 5 and 7 are novel pyrazolyl-chalcone derivatives that exert antitumor activity in pancreatic carcinoma.

Pyrazole moiety is a useful lead five-membered ring to synthesize many potent bioactive molecules for drug development with good safety profiles, particularly against different types of cancers.^8,9,37,64,65^ Anti-cancer activities of several pyrazole derivatives containing thiazoles and thiadiazoles compounds have been demonstrated in both *in vitro* and *in vivo* models, often resulting in promising lead compounds.^64,66,67^

Our data showed the novel compounds 4 and 7 had the most potent antitumor activity as they exhibited higher expression levels of Caspase-3, Caspase-8 and CDK6 genes in pancreatic cancer cell lines as well as higher DNA fragmentation rates, suggesting that they could inhibit the common apoptosis and oncogenic pathways. Moreover, it has been reported that the pyrazole compound (PCW-1001) increased apoptosis in several breast cancer cells, which is in agreement with our findings, and programmed cell death is an essential mechanism to eliminate cancer cells by anti-cancer drugs.^68–70^

It is well established that anti-cancer treatments, such as chemotherapy, induce DNA damage directly or indirectly in active proliferating cancer cells rather than non-proliferating normal cells.^71,72^ Our data showed that the newly synthesized compounds 4, 5 and 7 modulated apoptosis and anticancer-related genes in pancreatic cancer cell lines, suggesting that these compounds induce DNA damage response in pancreatic cancer cell lines due to the previous action of the gene modulation.

#### 2.2.4. Gene expression in PaCa-2 cancer cell lines

**Fig. 7:**
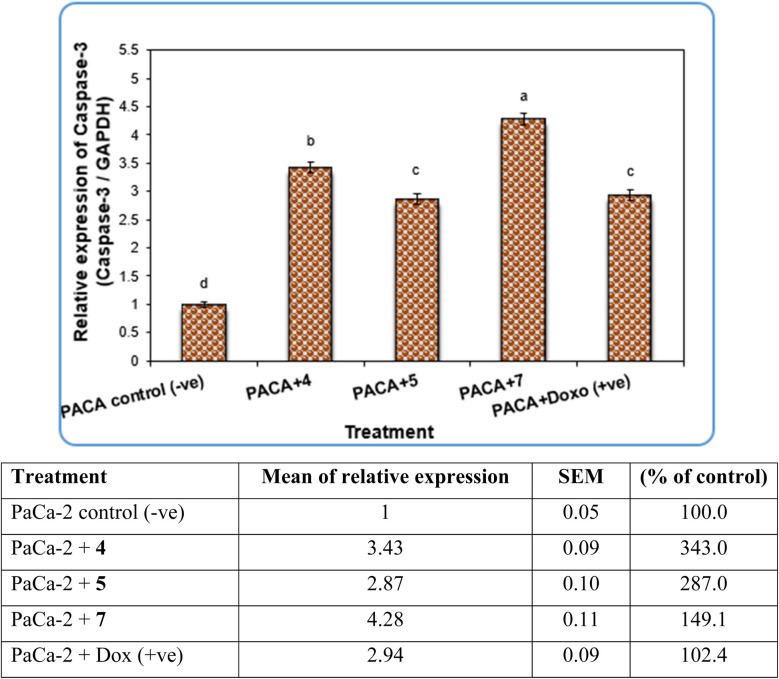
The alterations of the Caspase-3 gene in PaCa-2 cancer cell lines treated with compounds 4, 5 and 7 as well as Dox (+ve) control. Data are presented as mean ± SEM. a, b, c, d: mean values within tissue with unlike superscript letters were significantly different (*P* < 0.05).

Gene expression analysis in PaCa-2 cancer cell lines was performed using PaCa-2 cancer-related genes such as Caspase-3, CDK6 and Caspase-8. The results revealed that the expression levels of Caspase-3 and Caspase-8 genes were decreased significantly (*P* < 0.01) in negative samples of PaCa-2 cancer cell lines compared with treated PaCa-2 cell lines (Fig. 7 and 9, respectively). For the PaCa + 7 and PaCa + 4 groups, the expression levels of Caspase-3 and Caspase-8 were increased with high significant differences compared with the negative control. Additionally, for the PaCa + 4 and (+ve) control treated with doxorubicin, the expression levels of Caspase-3 and Caspase-8 were also increased significantly compared with the negative control, but their expression levels were lower than those in PaCa + 7 and PaCa + 4 groups (Fig. 7 and 9).

**Fig. 8:**
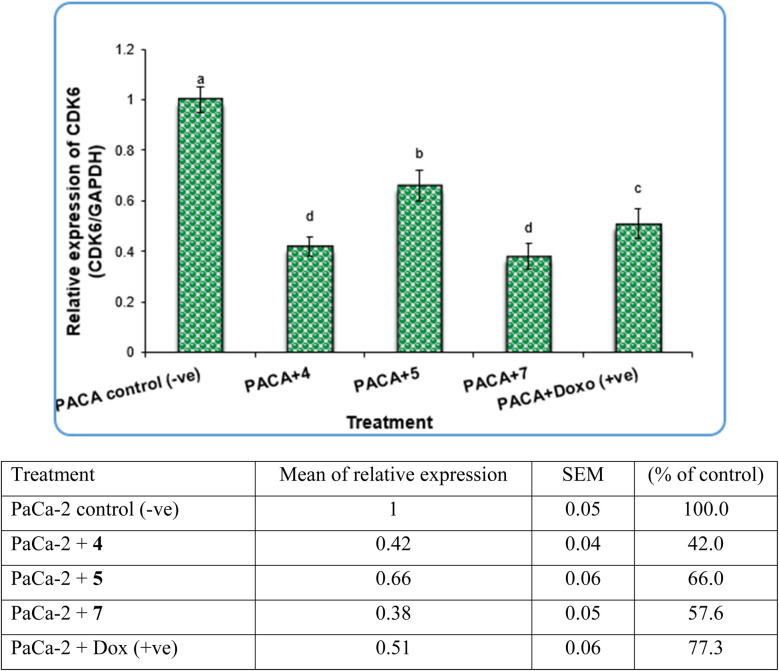
The alterations in the CDk6 gene in PaCa-2 cancer cell lines treated with compounds 4, 5 and 7 as well as Dox (+ve) control. Data are presented as mean ± SEM. a, b, c,d: mean values within tissue with unlike superscript letters were significantly different (*P* < 0.05).

In contrast, the expression levels of the CDK6 gene were increased significantly (*P* < 0.01) in negative samples of PaCa-2 cancer cell lines compared with treated cell lines (Fig. 8). For the PaCa + 7 and PaCa + 4 groups, the expression levels of CDK6 decreased with high significant differences compared with the negative control. Moreover, for the PaCa + 4 and (+ve) control treated with doxorubicin, the expression levels of CDK6 were also decreased significantly compared with the negative control, but their expression levels were higher than those in the PaCa + 7 and PaCa + 4 groups (Fig. 8).

**Fig. 9:**
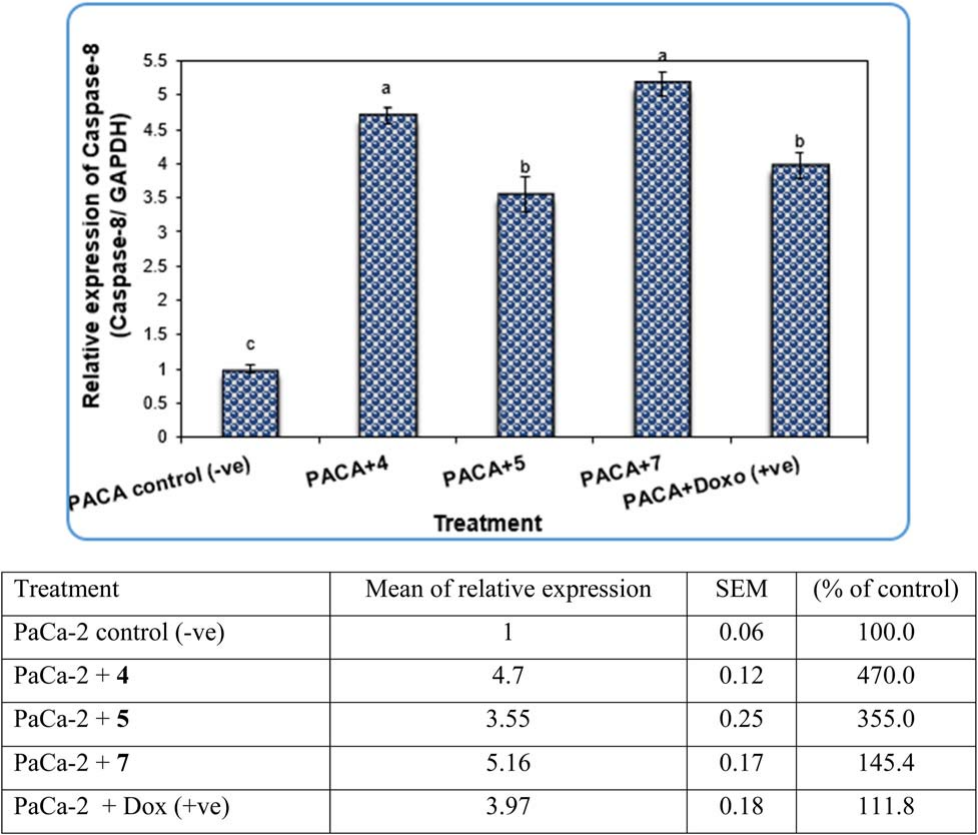
The alterations in the Caspase-3 gene in PaCa-2 cancer cell lines treated with compounds 4, 5 and 7 as well as Dox (+ve) control. Data are presented as mean ± SEM. a, b, c: mean values within tissue with unlike superscript letters were significantly different (*P* < 0.05).

#### 2.2.5. DNA damage in pancreatic cancer cell lines

**Fig. 10:**
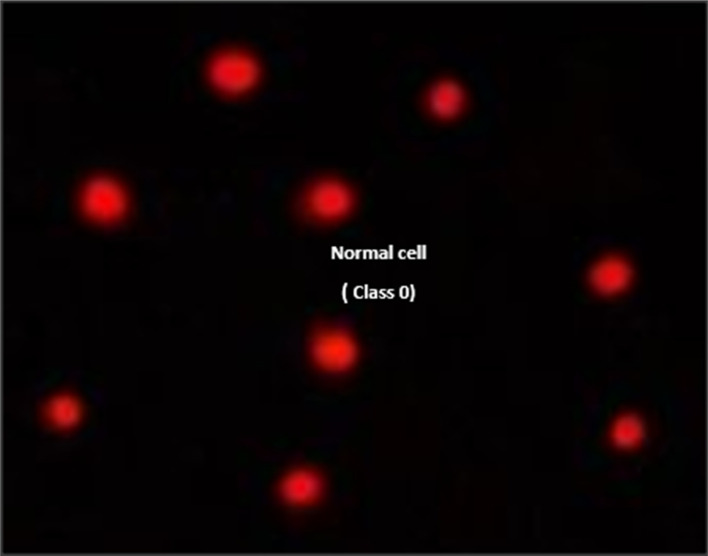
Visual score of normal DNA (class 0) using the comet assay in pancreatic cancer cell lines.

**Fig. 11:**
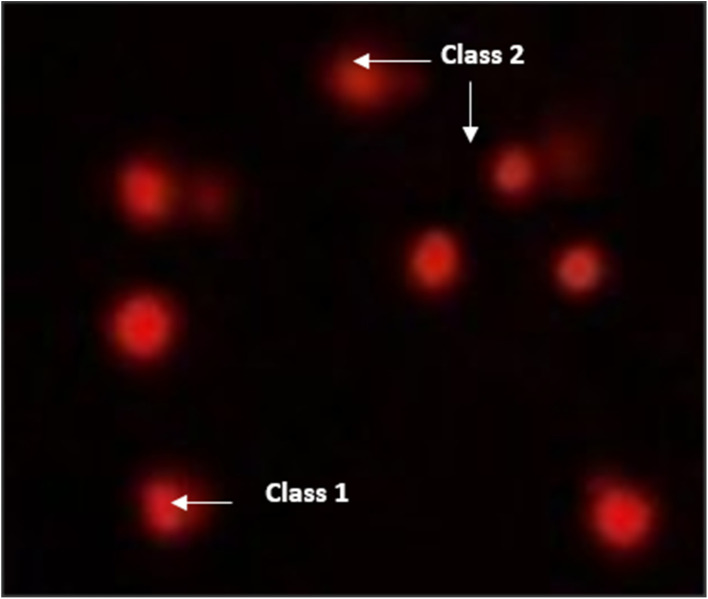
Visual score of DNA damage (classes 1 and 2) using the comet assay in pancreatic cancer cell lines exposed to 4, 5 and 7.

**Fig. 12:**
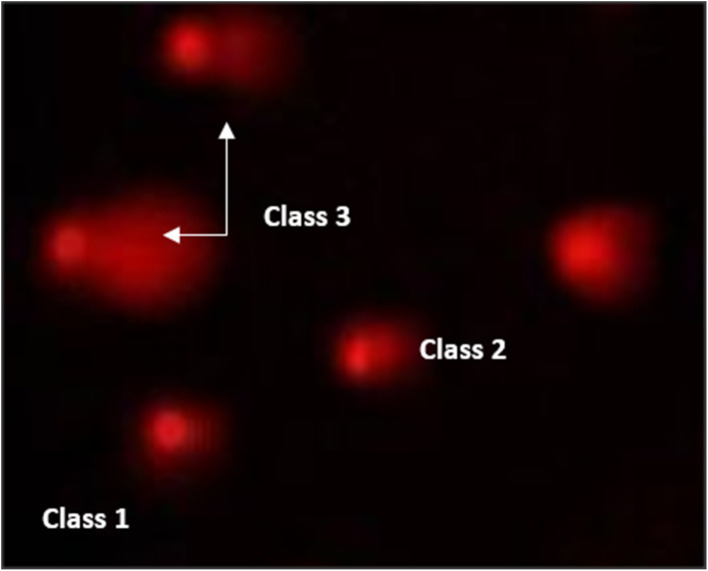
Visual score of DNA damage (classes 1, 2 and 3) using the comet assay in pancreatic cancer cell lines exposed to 4, 5 and 7.

Pancreatic cancer cell lines (PaCa-2) were used to assess the DNA damage induced by several compounds 4, 5 and 7 using comet assay, as shown in Table 4 and Fig. 10–12. The results exhibited that negative pancreatic cancer cell lines showed a significant reduction (*P* < 0.05) in DNA damage values (11.19 ± 0.75). Conversely, the DNA damage values were raised significantly (*P* < 0.01) in a treated pancreatic cancer cell line sample with compound 4 (28.81 ± 1.11), followed by PaCa-2 treated with compound 7 (26.43 ± 0.66), then by PaCa-2 treated with Dox (22.86 ± 1.75) and then PaCa-2 treated with compound 5 (21.19 ± 0.91) compared with the negative control (11.19 ± 0.75).

**Table 4:** Visual score of DNA damage in PaCa-2 cells treated with compounds 4, 5, and 7

Treatment	No of samples	No. of cells		Class^b^				DNA damaged cells% (mean ± SEM)
		Analyzed^a^	Comets	0	1	2	3	
PaCa-2 control (−ve)	4	420	47	373	31	10	6	11.19 ± 0.75^c^
PaCa-2 + 4	4	420	121	299	39	33	49	28.81 ± 1.11^a^
PaCa-2 + 5	4	420	89	331	42	26	21	21.19 ± 0.91^b^
PaCa-2 + 7	4	420	111	309	38	33	40	26.43 ± 0.66^a^
PaCa-2 + Dox (+ve)	4	420	96	324	34	37	25	22.86 ± 1.75^b^

a Number of cells examined per group.

b Class 0 = no tail; 1 = tail length < diameter of nucleus; 2 = tail length between 1× and 2× the diameter of nucleus; and 3 = tail length >2× the diameter of nucleus. Means with different superscripts (a, b and c) between groups in the same treatment are significantly different at *P* < 0.05. Data are presented as mean ± SEM.

DNA damage and repair are among the challenges that living cells face at all times, and the long-term survival of a healthy cell depends on successfully overcoming this challenge. In a variety of cancer cell lines, DOX has been shown to induce different types of cell death/growth arrest, including apoptosis and mitotic catastrophe, through inhibition of apoptosis by overexpression of Bcl-2 and necrosis.^73,74^ DOX can inhibit the synthesis of both DNA and RNA. In addition, DOX exerts its effects by preventing the uncoiling of the DNA enzyme topoisomerase II.^75^ In agreement with our finding, DOX drug induced a significantly high rate of DNA damage in PACA2 + Dox as compared with negative PaCa-2.

Free radicals, such as ROS, are naturally produced in living cells but could be increased by external sources, such as chemotherapy drugs (*e.g.*, DOX), X-rays, air pollutants or chemical compounds.^76,77^ So, the DNA damage induced by DOX in the present study could be induced by promoting ROS generation-mediated apoptosis. In the same trend, the used anti-cancer compounds 4, 5 and 7 exhibited more or less anti-cancer effects compared with DOX. Compound 4 revealed higher efficiency of anti-cancer effects as it induced DNA damage much more than DOX and other anti-cancer compounds. Thus, its anti-cancer effect could be attributed to its capacity to enhance ROS generation-mediated DNA damage and apoptosis.

#### 2.2.6. Flow cytometric cell cycle analysis

**Fig. 13:**
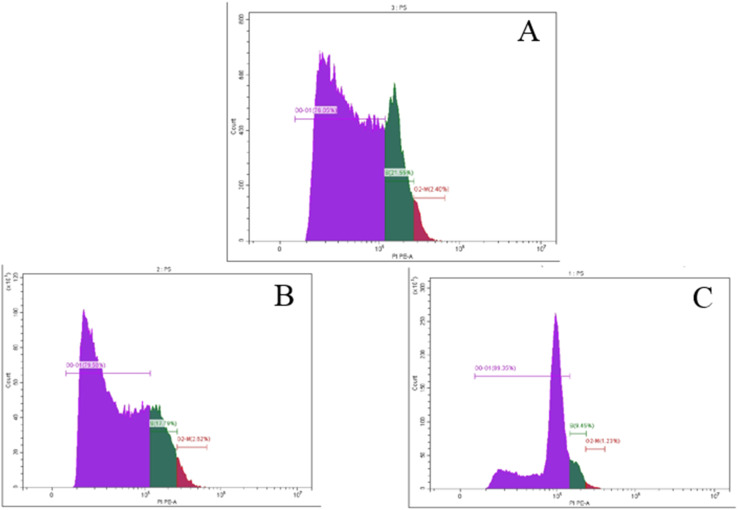
The cell distributions in the different phases of the cell cycle (G0/G1-S-G2/M) after 24 h analysed using flow cytometry: (A) 4-treated PaCa-2 cells, (B) PaCa-2 cells treated with doxorubicin as the positive (+ve) and (C) negative control (−ve).

The majority of cytotoxic substances inhibit the cell cycle at a specific stage in order to have an anti-proliferative effect. With an IC_50_ value of 13.0 μg mL^−1^, compound 4, which has the highest potency against pancreatic cells, was chosen to investigate its impact on the development of the cell cycle and induction of apoptosis in PaCa-2 cells. After incubating PaCa-2 cells with 13.0 μg mL^−1^ of compound 4 (Fig. 13A), the influence on the cell cycle distribution was evaluated by DNA flow cytometry analysis, and the cell cycle characteristics were compared to PaCa-2 cells treated with doxorubicin (Fig. 13B) and untreated control cells (Fig. 13C). The results are shown in Fig. 13. The results revealed that the treatment of PaCa-2 cells with compound 4 and positive control (Fig. 13A and B) showed an increase in the percentage of cells in the S phase (1.88-fold and 2.28-fold, respectively) and G2/M phase (2.13-fold and 1.95-fold, respectively) compared to the negative control (Fig. 13C).

#### 2.2.7. Structure–activity relationship (SAR)

**Fig. 14:**
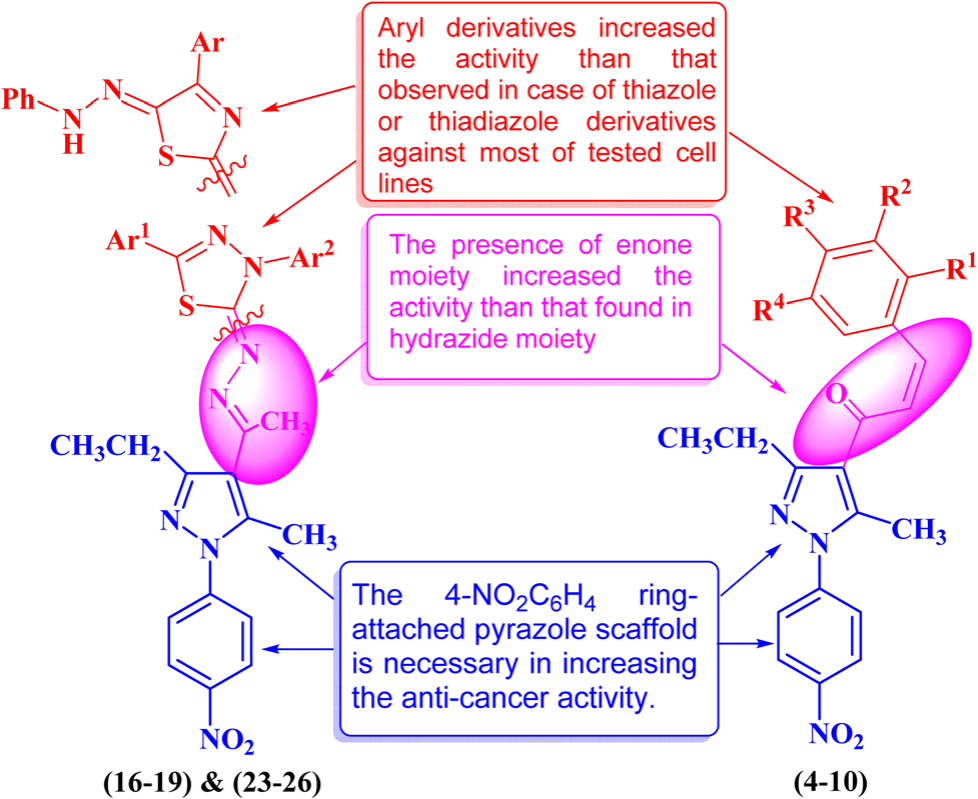
Structure–activity relationship study of the newly synthesized pyrazole derivatives.

As summarized in Fig. 14, the relation between the chemical structure of pyrazolyl-chalcone, pyrazolyl-thiazole and pyrazolyl-thiadiazole derivatives and their anti-cancer activity was explained. Our findings confirm that the pyrazolyl-chalcone series exhibits potent anti-cancer activity compared to pyrazolyl-thiazole and pyrazolyl-thiadiazole compounds. The presence of an α,β-unsaturated system in the enone moiety in compounds 4–10 increased the activity than that found in hydrazide moiety in compounds 16–19 and 23–26, especially on pancreatic cell lines. On the other hand, aryl derivatives attached to the chalcone moiety play a key role in increasing the activity than those observed in the case of thiazole or thiadiazole derivatives against most of the tested cell lines. Moreover, the pyrazolyl-chalcone derivatives are safer on the normal cell line (BJ1) than those found in the pyrazolyl-thiazole and pyrazolyl-thiadiazole derivatives.

## 3. Materials and methods

### 3.1. Chemistry

Melting points were measured with an Electrothermal 9100 apparatus and were uncorrected. The IR spectra were recorded using FTIR Bruker-vector 22 spectrophotometer as KBr pellets. The ^1^H and ^13^C NMR spectra were recorded in CDCl_3_ or DMSO-d_6_ as a solvent on a Varian Gemini NMR spectrometer at 300 MHz and 75 MHz, respectively, using TMS as the internal standard. Chemical shifts were reported as *δ* values in ppm. Mass spectra were recorded with a Shimadzu GCMS-QP-1000 EX mass spectrometer in an EI (70 eV) model. The elemental analyses were performed at the Microanalytical Center, Cairo University. The progress of the reaction was tracked by exposing silica gel G-coated TLC plates. The reagents, 1-(3-ethyl-5-methyl-1-(4-nitrophenyl)-1*H*-pyrazol-4-yl)ethan-1-one 2,^9,56^ methyl hydrazinecarbodithioate 11,^57,58^*N*-aryl-*C*-substituted methanohydrazonoyl chlorides 15^9,78,79^ and α-ketohydrazonoyl halide derivatives 20^80,81^ were prepared according to the literature.

#### 3.1.1 Synthesis of chalcone derivatives (4–10)

To a stirred mixture of the appropriate pyrazole 2 (0.6 g, 2 mmol) and the appropriate aldehyde 3 (2 mmol) in ethanol (30 mL), 20% sodium hydroxide solution was added, and the reaction mixture was stirred for 6 h at room temperature and left overnight. Reaction progress was monitored by TLC till the disappearance of the starting material. The resulting precipitated solid product was filtered, washed with water and crystallized from a suitable solvent to give the corresponding chalcones 4–10. Moreover, the purity of the final products was tested using the TLC technique to have one spot for each compound. The compounds prepared are listed below.

#### 3.1.2 1-(3-Ethyl-5-methyl-1-(4-nitrophenyl)-1*H*-pyrazol-4-yl)-3-phenylprop-2-en-1-one (4)

Yellow crystals (EtOH); mp 132–134 °C; yield (61%). IR (*ν*_max_, cm^−1^) *ν* 3425, 2978, 1654 (C=O), 1597, 1535, 1519, 1381, 1334, 1049, 964, 856, 983, 686, 563. ^1^H NMR (300 MHz, CDCl_3_) *δ* 1.35 (t, 3H, CH_3_, *J* ≈ 8 Hz), 2.62 (s, 3H, CH_3_), 2.98 (q, 2H, CH_2_, *J* ≈ 8 Hz), 7.20–7.73 (m, 9H, Ar–H), 8.40 (d, 2H, Ar–H, *J* ≈ 9 Hz). ^13^C NMR (75 MHz, CDCl_3_) *δ* 13.3, 13.2, 21.7, 115.1, 121.5, 124.5, 124.6, 127.6, 128.2, 129.1, 135.0, 142.7, 143.5, 145.5, 146.1, 154.1, 188.8. MS (EI, 70 eV) *m*/*z* (%): 361 (M^+^, 100), 346 (M–CH_3_, 77.93), 284 (M–C_6_H_5_, 39.98), 270 (M–(CH_3_ + C_6_H_5_), 35.21), 212 (M–(C_2_H_5_ + 4-NO_2_–C_6_H_4_), 25.73), 103 (C_6_H_5_–CH=CH_2_, 23.12), 77 (C_6_H_5_, 34.08). Anal. Calcd. for C_21_H_19_N_3_O_3_ (361.40): C, 69.79; H, 5.30; N, 11.63. Found: C, 69.87; H, 5.39; N, 11.55.

#### 3.1.3 3-(4-Chlorophenyl)-1-(3-ethyl-5-methyl-1-(4-nitrophenyl)-1*H*-pyrazol-4-yl)prop-2-en-1-one (5)

Yellow crystals (CH_3_CN); mp 162–164 °C; yield (63%). IR (*ν*_max_, cm^−1^) *ν* 2425, 1913, 1659 (C=O), 1597, 1512, 1334, 1049, 1010, 858, 825, 748, 686, 501. ^1^H NMR (300 MHz, CDCl_3_) *δ* 1.33 (t, 3H, CH_3_, *J* ≈ 8 Hz), 2.61 (s, 3H, CH_3_), 2.94 (q, 2H, CH_2_, *J* ≈ 8 Hz), 7.16–8.37 (m, 10H, Ar–H). ^13^C NMR (75 MHz, CDCl_3_) *δ* 13.2, 13.3, 21.8, 115.0, 121.1, 124.3, 124.5, 128.5, 129.1, 133.0, 133.3, 133.7, 142.5, 145.2, 145.2, 145.6, 154.0, 189.5. Anal. Calcd. for C_21_H_18_ClN_3_O_3_ (395.84): C, 63.72; H, 4.58; N, 10.62. Found: C, 63.80; H, 4.67; N, 10.71.

#### 3.1.4 1-(3-Ethyl-5-methyl-1-(4-nitrophenyl)-1*H*-pyrazol-4-yl)-3-(4-fluorophenyl)prop-2-en-1-one (6)

Yellow crystals (CH_3_CN); mp 152–154 °C; yield (63%). ^1^H NMR (300 MHz, CDCl_3_) *δ* 1.34 (t, 3H, CH_3_, *J* ≈ 8 Hz), 2.62 (s, 3H, CH_3_), 2.95 (q, 2H, CH_2_, *J* ≈ 8 Hz), 7.09–7.72 (m, 8H, Ar–H), 8.39 (d, 2H, Ar–H, *J* ≈ 9 Hz). ^13^C NMR (75 MHz, CDCl_3_) *δ* 12.8, 13.2, 21.3, 115.1, 115.5, 121.2, 124.2, 124.7, 130.3, 130.9, 142.6, 145.0, 145.3, 145.7, 154.2, 161.9, 187.8. Anal. Calcd. for C_21_H_18_FN_3_O_3_ (379.39): C, 66.48; H, 4.78; N, 11.08. Found: C, 66.56; H, 4.70; N, 11.16.

#### 3.1.5 1-(3-Ethyl-5-methyl-1-(4-nitrophenyl)-1*H*-pyrazol-4-yl)-3-(*p*-tolyl)prop-2-en-1-one (7)

Yellow crystals (EtOH); mp 136–138 °C; yield (61%). IR (*ν*_max_, cm^−1^) *ν* 3448, 3093, 2978, 2924, 1921, 1652 (C=O), 1589, 1527, 1334, 1049, 856, 694, 524, 501. ^1^H NMR (300 MHz, CDCl_3_) *δ* 1.34 (t, 3H, CH_3_, *J* ≈ 8 Hz), 2.40 (s, 3H, CH_3_), 2.61 (s, 3H, CH_3_), 2.95 (q, 2H, CH_2_, *J* ≈ 8 Hz), 7.14–7.72 (m, 8H, Ar–H), 8.39 (d, 2H, Ar–H, *J* ≈ 9 Hz). ^13^C NMR (75 MHz, CDCl_3_) *δ* 13.2, 13.3, 21.4, 21.9, 121.7, 124.6, 124.7, 125.2, 125.6, 128.2, 129.7, 131.8, 141.0, 142.9, 143.6, 146.5, 156.0, 187.5. MS (EI, 70 eV) *m*/*z* (%): 375 (M^+^, 100), 360 (M–CH_3_, 70.45), 284 (M–(C_6_H_4_–CH_3_), 22.41), 270 (M–(CH_3_ + C_6_H_4_–CH_3_), 17.85), 115 (CH_3_–C_6_H_4_–CH=CH_2_, 20.77), 76 (C_6_H_5_, 14.51). Anal. Calcd. for C_22_H_21_N_3_O_3_ (375.43): C, 70.38; H, 5.64; N, 11.19. Found: C, 70.47; H, 5.72; N, 11.23.

#### 3.1.6 1-(3-Ethyl-5-methyl-1-(4-nitrophenyl)-1*H*-pyrazol-4-yl)-3-(4-methoxyphenyl)prop-2-en-1-one (8)

Yellow crystals (MeOH); mp 102–104 °C; yield (60%). ^1^H NMR (300 MHz, CDCl_3_) *δ* 1.34 (t, 3H, CH_3_, *J* ≈ 8 Hz), 2.61 (s, 3H, CH_3_), 2.94 (q, 2H, CH_2_, *J* ≈ 8 Hz), 3.87 (s, 3H, OCH_3_), 6.94–7.72 (m, 8H, Ar–H), 8.39 (d, 2H, Ar–H, *J* ≈ 9 Hz). ^13^C NMR (75 MHz, CDCl_3_) *δ* 13.3, 13.4, 21.9, 55.4, 114.5, 121.9, 123.5, 124.7, 125.2, 127.3, 130.1, 142.8, 143.6, 143.9, 146.6, 156.0, 161.7, 187.6. Anal. Calcd. for C_22_H_21_N_3_O_4_ (391.43): C, 67.51; H, 5.41; N, 10.74. Found: C, 67.60; H, 5.51; N, 10.81.

#### 3.1.7 3-(2,4-Dimethoxyphenyl)-1-(3-ethyl-5-methyl-1-(4-nitrophenyl)-1*H*-pyrazol-4-yl)prop-2-en-1-one (9)

Yellow crystals (EtOH); mp 116–118 °C; yield (62%). ^1^H NMR (300 MHz, CDCl_3_) *δ* 1.33 (t, 3H, CH_3_, *J* ≈ 8 Hz), 2.55 (s, 3H, CH_3_), 2.95 (q, 2H, CH_2_, *J* ≈ 8 Hz), 3.86 (s, 3H, OCH_3_), 3.92 (s, 3H, OCH_3_), 6.47–8.38 (m, 9H, Ar–H). ^13^C NMR (75 MHz, CDCl_3_) *δ* 13.2, 13.6, 21.9, 55.3, 55.4, 98.4, 105.4, 115.7, 118.3, 121.1, 124.5, 125.0, 130.1, 139.9, 142.6, 143.7, 145.2, 145.7, 151.6, 161.7, 187.7. MS (EI, 70 eV) *m*/*z* (%): 421 (M^+^, 28.40), 390 (M–OCH_3_, 100), 257 (M–(CH_3_ + C_2_H_5_ + 4-NO_2_–C_6_H_4_), 54.26), 151 (2,4-di-OCH_3_–C_6_H_4_–CH_2_, 49.40), 138 (2,4-di-OCH_3_–C_6_H_4_, 20.60), 77 (C_6_H_5_, 14.35). Anal. Calcd. for C_23_H_23_N_3_O_5_ (421.45): C, 65.55; H, 5.50; N, 9.97. Found: C, 65.63; H, 5.59; N, 9.86.

#### 3.1.8 1-(3-Ethyl-5-methyl-1-(4-nitrophenyl)-1*H*-pyrazol-4-yl)-3-(3,4,5-trimethoxyphenyl)prop-2-en-1-one (10)

Yellow crystals (EtOH); mp 112–114 °C; yield (61%). IR (*ν*_max_, cm^−1^) *ν* 3425, 2962, 2864, 1650 (C=O), 1589, 1512, 1419, 1342, 1118, 1064, 987, 840, 758, 686, 609. ^1^H NMR (300 MHz, CDCl_3_) *δ* 1.34 (t, 3H, CH_3_, *J* ≈ 8 Hz), 2.60 (s, 3H, CH_3_), 2.93 (q, 2H, CH_2_, *J* ≈ 8 Hz), 3.91 (s, 3H, OCH_3_), 3.92 (s, 6H, 2OCH_3_), 6.83–7.72 (m, 6H, Ar–H), 8.39 (d, 2H, Ar–H, *J* ≈ 9 Hz). ^13^C NMR (75 MHz, CDCl_3_) *δ* 13.2, 13.4, 21.9, 56.2, 60.9, 105.5, 115.0, 121.7, 124.7, 125.2, 125.3, 130.1, 142.9, 143.8, 144.1, 146.6, 153.5, 156.0, 187.6. Anal. Calcd. for C_24_H_25_N_3_O_6_ (451.48): C, 63.85; H, 5.58; N, 9.31. Found: C, 63.97; H, 5.70; N, 9.42.

#### 3.1.9 Synthesis of 2-(1-(3-ethyl-5-methyl-1-(4-nitrophenyl)-1*H*-pyrazol-4-yl)ethylidene)hydrazine derivatives (13 and 14)

1-(3-Ethyl-5-methyl-1-(4-nitrophenyl)-1*H*-pyrazol-4-yl)ethan-1-one 2 (1.4 g, 5 mmol) was refluxed with methyl hydrazinecarbodithioate 11 (0.6 g, 5 mmol) or hydrazinecarbothioamide 12 (0.5 g, 5 mmol) in absolute ethanol (50 mL) for 2 h in the presence of a few drops of hydrochloric acid. Reaction progress was monitored by TLC till the disappearance of the starting material. The precipitated solid product was collected, washed with ethanol and crystallized from a suitable solvent to give 13 or 14, respectively. Moreover, the purity of the final products was tested using the TLC technique to have one spot for each compound. The compounds prepared are listed below.

#### 3.1.10 Methyl-2-(1-(3-ethyl-5-methyl-1-(4-nitrophenyl)-1*H*-pyrazol-4-yl)ethylidene)hydrazine-1-carbodithioate (13)

Yellow crystals (CH_3_CN); mp 160–162 °C; yield (72%). IR (*ν*_max_, cm^−1^) *ν* 3179 (NH), 2985, 2908, 1597, 1550, 1519, 1496, 1342, 1319, 1257, 1111, 1066, 964, 848, 748, 694, 578, 509, 478. ^1^H NMR (300 MHz, CDCl_3_) *δ* 1.34 (t, 3H, CH_3_, *J* ≈ 8 Hz), 2.35 (s, 3H, CH_3_), 2.52 (s, 3H, CH_3_), 2.61 (s, 3H, CH_3_), 2.94 (q, 2H, CH_2_, *J* ≈ 8 Hz), 7.73 (d, 2H, 4-NO_2_C_6_H_4_, *J* ≈ 9 Hz), 8.39 (d, 2H, 4-NO_2_C_6_H_4_, *J* ≈ 9 Hz), 10.03 (s, 1H, NH). ^13^C NMR (75 MHz, CDCl_3_) *δ* 13.1, 13.5, 17.1, 18.5, 21.6, 115.7, 124.6, 125.0, 142.7, 145.3, 145.7, 154.1, 159.3, 199.2. MS (EI, 70 eV) *m*/*z* (%): 377 (M^+^, 2.72), 329 (M–SCH_3_, 43.50), 296 (M–(S + SCH_3_), 37.82), 271 (M–(NH–C(S)–SCH_3_), 87.05), 225 (M–(CH_3_ + C_2_H_5_ + NH–C(S)–SCH_3_), 100), 117 ((N–NH–C(S)–SCH_3_), 71.75), 76 (C_6_H_4_, 98.26). Anal. Calcd. for C_16_H_19_N_5_O_2_S_2_ (377.48): C, 50.91; H, 5.07; N, 18.55. Found: C, 50.82; H, 5.18; N, 18.64.

#### 3.1.11 2-(1-(3-Ethyl-5-methyl-1-(4-nitrophenyl)-1*H*-pyrazol-4-yl)ethylidene)hydrazine-1-carbothioamide (14)

Yellow crystals (DMF); mp 192–194 °C; yield (74%). IR (*ν*_max_, cm^−1^) *ν* 3464 (NH), 3225 and 3298 (NH_2_), 2970, 2931, 1589, 1504, 1435, 1327, 1273, 1096, 1049, 956, 848, 601, 516. ^1^H NMR (300 MHz, DMSO-d_6_) *δ* 1.18 (t, 3H, CH_3_, *J* ≈ 8 Hz), 2.29 (s, 3H, CH_3_), 2.44 (s, 3H, CH_3_), 2.73 (q, 2H, CH_2_, *J* ≈ 8 Hz), 7.48 (s, 1H, NH_2_), 7.83 (d, 2H, 4-NO_2_C_6_H_4_, *J* ≈ 9 Hz), 8.23 (s, 1H, NH_2_), 8.38 (d, 2H, 4-NO_2_C_6_H_4_, *J* ≈ 9 Hz), 10.16 (s, 1H, NH). ^13^C NMR (75 MHz, DMSO-d_6_) *δ* 12.8, 13.7, 17.7, 21.5, 115.0, 124.3, 124.9, 142.6, 145.2, 145.6, 148.5, 154.2, 178.7. MS (EI, 70 eV) *m*/*z* (%): 346 (M^+^, 68.82), 331 (M–NH_2_, 45.90), 271 (M–(NH–C(S)–NH_2_), 75.76), 225 (M–(4-NO_2_–C_6_H_4_), 38.60), 117 (H_2_N–C(S)–NH–N=C–CH_3_, 56.08), 76 (C_6_H_4_, or H_2_N–C(S)–NH, 76.87), 60 (H_2_N–C=S, 100). Anal. Calcd. for C_15_H_18_N_6_O_2_S (346.41): C, 52.01; H, 5.24; N, 24.26. Found: C, 52.11; H, 5.33; N, 24.37.

#### 3.1.12 Synthesis of 2-((1-(3-ethyl-5-methyl-1-(4-nitrophenyl)-1*H*-pyrazol-4-yl)ethylidene)hydrazono)-2,3-dihydro-1,3,4-thiadiazole derivatives (16–19)

Method A: a mixture of methyl 2-(1-(3-ethyl-5-methyl-1-(4-nitrophenyl)-1*H*-pyrazol-4-yl)ethylidene) hydrazine-1-carbodithioate 13 (0.4 g, 1 mmol) and the appropriate hydrazonoyl chlorides 15 (1 mmol) was dissolved in absolute ethanol (20 mL). To the resulting solution, triethylamine was added, and the reaction mixture was stirred for 6 h at room temperature. Reaction progress was monitored by TLC till the disappearance of the starting material. The resulting solid product that precipitated was collected, washed with ethanol and crystallized from dimethylformamide to give the corresponding thiadiazole derivatives 16–19.

Method B: refluxing of 2-(1-(3-ethyl-5-methyl-1-(4-nitrophenyl)-1*H*-pyrazol-4-yl)ethylidene)hydrazine-1-carbothioamide 14 (0.3 g, 1 mmol) with the appropriate hydrazonoyl chlorides 15 (1 mmol) in absolute ethanol (20 mL) was performed in the presence of triethylamine for 4 h. Reaction progress was monitored by TLC till the disappearance of the starting material. The resulting solid product that precipitated was collected, washed with ethanol and crystallized from dimethylformamide to afford the same products 16–19. Moreover, the purity of the final products was tested using the TLC technique to have one spot for each compound. The compounds prepared are listed below.

#### 3.1.13 2-((1-(3-Ethyl-5-methyl-1-(4-nitrophenyl)-1*H*-pyrazol-4-yl)ethylidene)hydrazono)-3,5-diphen-yl-2,3-dihydro-1,3,4-thiadiazole (16)

Yellow crystals; yield (61%); mp 184–186 °C. ^1^H NMR (300 MHz, CDCl_3_) *δ* 1.35 (t, 3H, CH_3_, *J* ≈ 8 Hz), 2.51 (s, 3H, CH_3_), 2.60 (s, 3H, CH_3_), 2.92 (q, 2H, CH_2_, *J* ≈ 8 Hz), 7.27–7.83 (m, 10H, Ar–H), 8.26 (d, 2H, Ar–H), 8.38 (d, *J* ≈ 9 Hz, 2H, Ar–H). ^13^C NMR (75 MHz, CDCl_3_) *δ* 13.4, 13.6, 19.2, 21.5, 120.5, 121.1, 124.5, 124.6, 125.5, 126.1, 128.6, 128.9, 130.3, 130.5, 138.4, 140.0, 144.6, 145.9, 150.2, 155.0, 155.7, 163.9. Anal. Calcd. for C_28_H_25_N_7_O_2_S (523.62): C, 64.23; H, 4.81; N, 18.73. Found: C, 64.33; H, 4.90; N, 18.85.

#### 3.1.14 2-((1-(3-Ethyl-5-methyl-1-(4-nitrophenyl)-1*H*-pyrazol-4-yl)ethylidene)hydrazono)-3-phenyl-5-(styryl)-2,3-dihydro-1,3,4-thiadiazole (17)

Yellow crystals; yield (64%); mp 206–208 °C. ^1^H NMR (300 MHz, CDCl_3_) *δ* 1.36 (t, 3H, CH_3_, *J* ≈ 8 Hz), 2.50 (s, 3H, CH_3_), 2.61 (s, 3H, CH_3_), 2.92 (q, 2H, CH_2_, *J* ≈ 8 Hz), 6.94–7.54 (m, 10H, Ar–H), 7.74 (d, 2H, Ar–H, *J* ≈ 9 Hz), 8.19 (d, 2H, Ar–H, *J* ≈ 8 Hz), 8.38 (d, 2H, Ar–H, *J* ≈ 9 Hz). ^13^C NMR (75 MHz, CDCl_3_) *δ* 13.5, 13.6, 19.3, 21.6, 118.9, 120.5, 121.2, 124.5, 124.6, 125.6, 127.1, 128.7, 128.9, 129.3, 135.2, 136.7, 138.5, 139.9, 144.6, 145.9, 150.4, 155.1, 156.0, 163.0, 128.9, 129.2, 135.1, 136.6, 138.6, 139.8, 144.4, 146.0, 149.8, 150.4, 156.0, 163.0. MS (EI, 70 eV) *m*/*z* (%): 549 (M^+^, 13.99), 256 (4-NO_2_–C_6_H_4_)–(CH_3_)–pyrazole–(C_2_H_5_)–CH–CH_3_)^+^, 20.85), 242 ((4-NO_2_–C_6_H_4_)–(CH_3_)–pyrazole–C(N)–CH_3_), 30.84), 91 (C_6_H_5_–CH^+^, 100), 77 (C_6_H_4_, 44.57). Anal. Calcd. for C_30_H_27_N_7_O_2_S (549.65): C, 65.56; H, 4.95; N, 17.84. Found: C, 65.67; H, 4.86; N, 17.93.

#### 3.1.15 2-((1-(3-Ethyl-5-methyl-1-(4-nitrophenyl)-1*H*-pyrazol-4-yl)ethylidene)hydrazono)-5-(furan-2-yl)-3-(4-nitrophenyl)-2,3-dihydro-1,3,4-thiadiazole (18)

Yellow crystals; yield (61%); mp 242–244 °C. ^1^H NMR (300 MHz, CDCl_3_) *δ* 1.36 (t, 3H, CH_3_, *J* ≈ 8 Hz), 2.55 (s, 3H, CH_3_), 2.60 (s, 3H, CH_3_), 2.92 (q, 2H, CH_2_, *J* ≈ 8 Hz), 6.61–8.54 (m, 11H, Ar–H). ^13^C NMR (75 MHz, CDCl_3_) *δ* 13.5, 14.5, 19.1, 21.7, 109.4, 110.2, 113.0, 115.4, 124.5, 124.6, 125.5, 138.2, 141.8, 142.0, 143.2, 144.5, 145.2, 146.0, 154.3, 155.0, 158.4, 160.2. MS (EI, 70 eV) *m*/*z* (%): 559 (M^+^, 34.40), 256 ((4-NO_2_–C_6_H_4_)–(CH_3_)–C_2_H_5_–pyrazole–C–CH_3_)^2+^, 44.60, 242 ((4-NO_2_–C_6_H_4_)–(CH_3_)–C_2_H_5_–pyrazole–C)^2+^, 52.70), 122 (4-NO_2_–C_6_H_4_, 17.98), 76 (C_6_H_4_, 20.85). Anal. Calcd. for C_26_H_22_N_8_O_5_S (558.57): C, 55.91; H, 3.97; N, 20.06. Found: C, 55.99; H, 3.88; N, 20.15.

#### 3.1.16 2-((1-(3-Ethyl-5-methyl-1-(4-nitrophenyl)-1*H*-pyrazol-4-yl)ethylidene)hydrazono)-3-(4-nitro-phenyl)-5-(thiophen-2-yl)-2,3-dihydro-1,3,4-thiadiazole (19)

Yellow crystals; yield (61%); mp 248–250 °C. ^1^H NMR (300 MHz, CDCl_3_) *δ* 1.37 (t, 3H, CH_3_, *J* ≈ 8 Hz), 2.56 (s, 3H, CH_3_), 2.62 (s, 3H, CH_3_), 2.91 (q, 2H, CH_2_, *J* ≈ 8 Hz), 7.15–8.52 (m, 11H, Ar–H). ^13^C NMR (75 MHz, CDCl_3_) *δ* 13.5, 14.5, 19.0, 21.7, 113.2, 115.3, 124.5, 124.7, 124.8, 127.2, 127.4, 129.6, 134.8, 137.9, 138.5, 142.8, 145.0, 145.4, 145.8, 151.7, 158.3, 159.6. MS (EI, 70 eV) *m*/*z* (%): 575 (M^+^, 36.00), 574 (M, 100), 256 ((4-NO_2_–C_6_H_4_)–(CH_3_)–C_2_H_5_–pyrazole–C–CH_3_)^2+^, 44.11, 242 ((4-NO_2_–C_6_H_4_)–(CH_3_)–C_2_H_5_–pyrazole–C)^2+^, 47.12, 76 (C_6_H_4_, 26.55). Anal. Calcd. for C_26_H_22_N_8_O_4_S_2_ (574.63): C, 54.35; H, 3.86; N, 19.50. Found: C, 54.46; H, 3.97; N, 19.60.

#### 3.1.17 Synthesis of 5-((1-(3-ethyl-5-methyl-1-(4-nitrophenyl)-1*H*-pyrazol-4-yl)ethylidene)hydrazono)-4-phenyl-4,5-dihydro-1,3,4-thiadiazole derivatives (23–26)

A mixture of compound 14 (0.3 g, 1 mmol) with the appropriate α-ketohydrazonoyl halides 20 (1 mmol) in absolute ethanol (20 mL) was refluxed in the presence of triethylamine for 4 h. Reaction progress was monitored by TLC till the disappearance of the starting material. The resulting solid product that precipitated was collected, washed with ethanol and crystallized from a suitable solvent to give products 23–26. Moreover, the purity of the final products was tested using the TLC technique to have one spot for each compound. The compounds prepared are listed below.

#### 3.1.18 2-((1-(3-Ethyl-5-methyl-1-(4-nitrophenyl)-1*H*-pyrazol-4-yl)ethylidene)hydrazono)-4-methyl-5-(2-phenylhydrazono)-2,5-dihydrothiazole (23)

Orange crystals (CH_3_CN), mp 204–206 °C, yield (63%). ^1^H NMR (300 MHz, CDCl_3_) *δ* 1.34 (t, 3H, CH_3_, *J* ≈ 8 Hz), 2.01 (s, 3H, CH_3_), 2.61 (s, 3H, CH_3_), 2.62 (s, 3H, CH_3_), 2.97 (q, 2H, CH_2_, *J* ≈ 8 Hz), 7.03–7.37 (m, 5H, Ar–H), 7.49 (s, 1H, NH), 7.72 (d, 2H, Ar–H, *J* ≈ 9 Hz), 8.39 (d, 2H, Ar–H, *J* ≈ 9 Hz). ^13^C NMR (75 MHz, CDCl_3_) *δ* 13.5, 13.9, 16.7, 19.2, 21.9, 114.1, 115.0, 122.9, 124.7, 125.0, 129.5, 141.9, 142.4, 144.7, 145.2, 148.4, 154.9, 157.9, 161.4, 174.6. MS (EI, 70 eV) *m*/*z* (%): 489 (M^+^, 3.18), 488 (M, 10.01), 270 ((4-NO_2_–C_6_H_4_)–(CH_3_)–pyrazole–(C_2_H_5_)–C(N)–CH_3_), 54.54), 225 ((4-NO_2_–C_6_H_4_)–(CH_3_)–pyrazole–C–CH_3_)^2+^, 21.35, 117 ((C_6_H_5_–NH–N–C)^2+^, 40.29), 92 (C_6_H_5_–NH, 33.26), 77 (C_6_H_5_, 100). Anal. Calcd. for C_24_H_24_N_8_O_2_S (488.57): C, 59.00; H, 4.95; N, 22.94. Found: C, 59.09; H, 4.86; N, 22.85.

#### 3.1.19 2-((1-(3-Ethyl-5-methyl-1-(4-nitrophenyl)-1*H*-pyrazol-4-yl)ethylidene)hydrazono)-4-phenyl-5-(2-phenylhydrazono)-2,5-dihydrothiazole (24)

Red crystals (CH_3_CN), mp 218–220 °C, yield (61%). ^1^H NMR (300 MHz, CDCl_3_) *δ* 1.36 (t, 3H, CH_3_, *J* ≈ 8 Hz), 2.65 (s, 3H, CH_3_), 2.66 (s, 3H, CH_3_), 2.97 (q, 2H, CH_2_, *J* ≈ 8 Hz), 7.07–7.40 (m, 5H, Ar–H), 7.48 (s, 1H, NH), 7.54–8.49 (m, 9H, Ar–H). ^13^C NMR (75 MHz, CDCl_3_) *δ* 13.3, 13.7, 19.2, 21.7, 114.2, 115.0, 122.2, 124.4, 124.7, 128.4, 129.2, 129.9, 132.8, 142.6, 143.1, 144.9, 145.4, 148.1, 154.0, 158.9, 160.7, 174.9. MS (EI, 70 eV) *m*/*z* (%): 551 (M^+^, 4.23), 550 (M, 11.70), 271 ((4-NO_2_–C_6_H_4_)–(CH_3_)–pyrazole–(C_2_H_5_)–C(N)–CH_3_), 32.86), 225 ((4-NO_2_–C_6_H_4_)–(CH_3_)–pyrazole–C–CH_3_)^2+^, 13.39), 117 ((C_6_H_5_–NH–N–C)^2+^, 28.63), 92 (C_6_H_5_–NH, 20.75), 77 (C_6_H_5_, 100). Anal. Calcd. for C_29_H_26_N_8_O_2_S (550.64): C, 63.26; H, 4.76; N, 20.35. Found: C, 63.35; H, 4.83; N, 20.43.

#### 3.1.20 2-((1-(3-Ethyl-5-methyl-1-(4-nitrophenyl)-1*H*-pyrazol-4-yl)ethylidene)hydrazono)-4-(furan-2-yl)-5-(2-phenylhydrazono)-2,5-dihydrothiazole (25)

Red crystals (DMF), mp 214–216 °C, yield (65%). IR (*ν*_max_, cm^−1^) *ν* 3433 (NH), 2970, 2877, 1658, 1519, 1442, 1334, 1249, 1111, 758, 686, 624. ^1^H NMR (300 MHz, CDCl_3_) *δ* 1.34 (t, 3H, CH_3_, *J* ≈ 8 Hz), 2.56 (s, 3H, CH_3_), 2.65 (s, 3H, CH_3_), 2.90 (q, 2H, CH_2_, *J* ≈ 8 Hz), 6.71–7.84 (m, 11H, Ar–H), 8.41 (d, 2H, Ar–H, *J* ≈ 9 Hz). ^13^C NMR (75 MHz, CDCl_3_) *δ* 13.5, 13.9, 19.4, 21.9, 109.2, 109.7, 114.4, 116.0, 122.7, 124.6, 124.9, 129.6, 137.6, 139.9, 142.5, 143.3, 144.1, 145.3, 146.4, 153.9, 158.8, 160.6, 174.8. Anal. Calcd. for C_27_H_24_N_8_O_3_S (540.60): C, 59.99; H, 4.48; N, 20.73. Found: C, 59.91; H, 4.57; N, 20.81.

#### 3.1.21 2-((1-(3-Ethyl-5-methyl-1-(4-nitrophenyl)-1*H*-pyrazol-4-yl)ethylidene)hydrazono)-5-(2-phenyl-hydrazono)-4-(thiophen-2-yl)-2,5-dihydrothiazole (26)

Red crystals (DMF), mp 212–214 °C, yield (60%). ^1^H NMR (300 MHz, CDCl_3_) *δ* 1.35 (t, 3H, CH_3_, *J* ≈ 8 Hz), 2.63 (s, 3H, CH_3_), 2.65 (s, 3H, CH_3_), 2.97 (q, 2H, CH_2_, *J* ≈ 8 Hz), 7.08–7.42 (m, 5H, Ar–H), 7.54 (s, 1H, NH), 7.70–8.52 (m, 7H, Ar–H); ^13^C NMR (75 MHz, CDCl_3_) *δ* 13.5, 13.9, 19.3, 21.9, 114.5, 116.0, 122.9, 123.0, 123.0, 124.7, 125.0, 125.9, 128.2, 129.6, 134.6, 139.8, 143.1, 144.3, 146.3, 150.6, 156.0, 162.6, 177.8. MS (EI, 70 eV) *m*/*z* (%): 557 (M^+^, 2.08), 528 (M–C_2_H_5_, 14.58), 271 ((4-NO_2_–C_6_H_4_)–(CH_3_)–pyrazole–(C_2_H_5_)–C(N)–CH_3_), 38.01), 117 ((C_6_H_5_–NH–N–C)^2+^, (39.22), 77 (C_6_H_5_, 100), 65 (pyrazole ring, 22.68). Anal. Calcd. for C_27_H_24_N_8_O_2_S_2_ (556.66): C, 58.26; H, 4.35; N, 20.13. Found: C, 58.35; H, 4.46; N, 20.22.

### 3.2. Anti-cancer evaluation

Cell viability was assessed by the mitochondrial-dependent reduction of yellow MTT (3-(4,5-dimethylthiazol-2-yl)-2,5-diphenyl tetrazolium bromide) to purple formazan. Procedure: all the following procedures were done in a sterile area using a Laminar flow cabinet biosafety class II level (Baker, SG403INT, Sanford, ME, USA). Cells were suspended in DMEM-F12 medium (for PaCa-2, MCF7, and PC3) beside one normal cell line (BJ1), 1% antibiotic–antimycotic mixture (10 000 U mL^−1^ potassium penicillin, 10 000 μg mL^−1^ streptomycin sulfate and 25 μg mL^−1^ amphotericin B) and 1% l-glutamine at 37 °C under 5% CO_2_.^82^

Cells were cultured for 10 days, then seeded at a concentration of 10 × 10^3^ cells per well in a fresh complete growth medium in 96-well microtiter plastic plates at 37 °C for 24 h under 5% CO_2_ using a water-jacketed carbon dioxide incubator (Sheldon, TC2323, Cornelius, OR, USA). Media was aspirated, fresh medium (without serum) was added, and cells were incubated either alone (negative control) or with different concentrations of the sample to give a final concentration of (100, 50, 25, 12.5, 6.25, 3.125, 0.78 and 1.56 μg mL^−1^). After 48 h of incubation, the medium was aspirated, 40 μL MTT salt (2.5 μg mL^−1^) was added to each well and incubated for a further four hours at 37 °C under 5% CO_2_. To stop the reaction and dissolve the formed crystals, 200 μL of 10% sodium dodecyl sulfate (SDS) in deionized water was added to each well and incubated overnight at 37 °C. A positive control composed of 100 μg mL^−1^ was used as a known cytotoxic natural agent that gives 100% lethality under the same conditions.^82^

The absorbance was then measured using a microplate multi-well reader (Bio-Rad Laboratories Inc., model 3350, Hercules, California, USA) at 595 nm and a reference wavelength of 620 nm. A statistical significance was tested between samples and the negative control using an independent *t*-test by the SPSS 11 program. DMSO is the vehicle used for the dissolution of plant extracts, and its final concentration in the cells was less than 0.2%. The percentage of change in viability was calculated according to the formula: ((reading of extract/reading of negative control) – 1) × 100. A probit analysis was carried out for IC_50_ determination using the SPSS 11 program. In the present study, the degree of selectivity of the synthetic compounds is expressed as SI = IC_50_ of a pure compound in a normal cell line/IC_50_ of the same pure compound in the cancer cell line, where IC_50_ is the concentration required to kill 50% of the cell population.

### 3.3. DNA fragmentation assay

The DNA fragmentation assay was performed in accordance with the protocol established by Yawata (1998)^83^ with some modifications. Briefly, after 24 h of exposure of PaCa-2 cell lines to the tested substances (H20) and doxorubicin in different Petri dishes (60 × 15 mm, Greiner), the cells were trypsinized, suspended, homogenized in 1 mL of medium and centrifuged (10 min at 800 rpm). Low-molecular-weight genomic DNA was extracted as described by Yawata (1998).^83^ Approximately 1 × 10^6^ cells were plated and treated with the tested substances in various experiments. All the cells (including floating cells) were harvested by trypsinization and washed with Dulbecco's Phosphate Buffered Saline. Cells were lysed with the lysis buffer containing 10 mM Tris (pH 7.4), 150 mM NaCl, 5 mM ethylenediaminetetraacetic acid (EDTA), and 0.5% Triton X-100 for 30 min on ice. Lysates were vortexed and cleared by centrifugation at 10 000*g* for 20 min. Fragmented DNA in the supernatant was extracted with an equal volume of neutral phenol : chloroform : isoamyl alcohol mixture (25 : 24 : 1) and analyzed electrophoretically on 2% agarose gels containing 0.1 μg mL^−1^ ethidium bromide.^29,84^

### 3.4. Gene expression analysis

#### 3.4.1. RNA isolation and reverse transcription (RT) reaction

RNeasy Mini Kit (Qiagen, Hilden, Germany) supplemented with DNaseI (Qiagen) digestion step was used to isolate total RNA from PaCa-2 cell line samples according to the manufacturer's protocol. Isolated total RNA was treated with one unit of RQ1 RNAse-free DNAse (Invitrogen, Germany) to digest DNA residues, re-suspended in DEPC-treated water and quantified photospectrometrically at 260 nm. The purity of total RNA was assessed by the 260/280 nm ratio, which was between 1.8 and 2.1. Additionally, integrity was assured with ethidium bromide-stain analysis of 28S and 18S bands by the formaldehyde-containing agarose gel electrophoresis. Aliquots were used immediately for reverse transcription (RT), otherwise they were stored at −80 °C.

Complete poly(A)^+^ RNA isolated from PaCa-2 cell line samples was reverse transcribed into cDNA in a total volume of 20 μL using RevertAid™ First Strand cDNA Synthesis Kit (Fermentas, Germany). An amount of total RNA (5 μg) was used with a master mix. The master mix consisted of 50 mM MgCl_2_, 10× RT buffer (50 mM KCl; 10 mM Tris–HCl; pH 8.3), 10 mM of each dNTP, 50 μM oligo-dT primer, 20 IU ribonuclease inhibitor (50 kDa recombinant enzyme to inhibit RNase activity) and 50 IU MuLV reverse transcriptase. The mixture of each sample was centrifuged for 30 s at 1000 g and transferred to the thermocycler. The RT reaction was carried out at 25 °C for 10 min, followed by 1 h at 42 °C, and finished with a denaturation step at 99 °C for 5 min. Afterward, the reaction tubes containing RT preparations were flash-cooled in an ice chamber until being used for cDNA amplification through quantitative real-time-polymerase chain reaction (qRT-PCR).

#### 3.4.2. Real-time-PCR (qPCR)

Determination of the PaCa-2 cell line cDNA copy number was carried out using the StepOne™ Real-Time PCR System from Applied Biosystems (Thermo Fisher Scientific, Waltham, MA USA). PCR reactions were set up in 25 μL reaction mixtures containing 12.5 μL 1× SYBR® Premix Ex TaqTM (TaKaRa, Biotech. Co. Ltd), 0.5 μL 0.2 μM sense primer, 0.5 μL 0.2 μM antisense primer, 6.5 μL distilled water, and 5 μL of cDNA template. The reaction program was allocated to 3 steps. The first step was at 95 °C for 3 min. The second step consisted of 40 cycles in which each cycle was divided into 3 steps: (a) at 95 °C for 15 s, (b) at 55 °C for 30 s, and (c) at 72 °C for 30 s. The third step consisted of 71 cycles, which started at 60.0 °C and then increased by about 0.5 °C every 10 s up to 95 °C. At the end of each qRT-PCR, a melting curve analysis was performed at 95 °C to check the quality of the used primers. Each experiment included a distilled water control. The sequences of specific primers of the PaCa-2 cell line-related genes, such as Caspase-3, CDK6 (Cyclin-dependent kinase 6) and Caspase-8 genes, were designed and are listed in Table 5. The relative quantification of the target to the reference was determined by using the 2^−ΔΔCT^ method (Watanabe *et al.*, 2010; Yang *et al.*, 2017).^85–87^

**Table 5:** Primer sequences used for qRT-PCR of PaCa-2 cancer cell lines^a^

Gene	Primer sequence	GenBank (accession no.)
Caspase-3	F: ACT GGA CTG TGG CAT TGA GA	AJ413269.1
	R: GCA CAA AGC GAC TGG ATG AA	
CDK6	F: TCT CCC GGC ACT TCT GAA AT	NM_001145306.2
	R: ACA CCA GGT AGA AGG ACT GC	
Caspase-8	F: TTG TGG TAC CCT GCC TAG TG	AH007578.2
	R: TGG TTT CTC TCT CAC GCC TT	
GAPDH	F: CAC ATC GCT CAG ACA CCA TG	NM_001289746.2
	R: TGA CGG TGC CAT GGA ATT TG	

a Caspase-3; CDK6: cyclin dependent kinase 6; aaspase-8; GAPDH: glyceraldehyde-3-phosphate dehydrogenase.

### 3.5. Determination of the DNA damage using comet assay

The DNA damage using comet assay was determined using pancreatic cancer cell lines (PaCa-2) according to Olive *et al.* (1990).^88^ After the trypsin treatment to produce a single cell suspension, approximately 1.5 × 10^4^ cells were embedded in 0.75% low-gelling-temperature agarose and rapidly pipetted onto a pre-coated microscope slide. Cell samples were lysed for 4 h at 50 °C in 0.5% SDS and 30 mM EDTA at pH 8.0. After rinsing overnight at room temperature in tris/borate/EDTA buffer, pH 8.0, samples were electrophoresed for 25 min at 0.6 V cm^−1^, then stained with propidium iodide. Slides were viewed using a fluorescence microscope with a CCD camera, and 150 individual comet images were analyzed from each sample for the tail moment, DNA content, and percentage DNA in the tail. For each sample, about 100 cells were examined to determine the percentage of cells with DNA damage that appear like comets. The non-overlapping cells were randomly selected and were visually assigned a score on an arbitrary scale of 0–3 (*i.e.*, class 0 = no detectable DNA damage and no tail; class 1 = tail with a length less than the diameter of the nucleus; class 2 = tail with a length between 1× and 2× the nuclear diameter; and class 3 = tail longer than 2× the diameter of the nucleus) based on perceived comet tail length migration and relative proportion of DNA in the nucleus (Collins *et al.*, 1997).^89^

### 3.6. Cell cycle analysis assay of treated PaCa-2 cells

Tubes with cell suspensions were received from the client's own lab. Cells (1 × 10^6^) were suspended in 0.5 mL 1× DPBS and aspirated several times with a Pasteur pipet and fixed with 70% ethanol on ice for 2 h. The ethanol-suspended cells were centrifuged for 5 min at 300*g* and decanted ethanol thoroughly. The cell pellets were re-suspended in 5 mL 1× DPBS for 30 s and were centrifuged at 300*g* for 5 min. The cell pellets were re-suspended in 1 mL of the PI staining solution and kept in the dark at room temperature for 30 min. Cells were then transferred to the CytoFLEX Flow Cytometer (Beckman Coulter Life Sciences, USA) to measure the cell fluorescence (BECKMAN COULTER Inc., Cairo, Egypt and Cat. No. 4238055-CB) for cell cycle analysis. The percentage of cells in the G0/G1, S, and G2/M phases of the cell cycle was calculated using CytExpert Software.

### 3.7. Statistical analysis

All data were analyzed using the General Linear Models (GLM) procedure of Statistical Analysis System (1982),^90^ followed by the Scheffé test to assess significant differences between groups. The values are expressed as mean ± SEM. All statements of significance were based on the probability of *P* < 0.05.

## 4. Conclusion

In summary, in this work, we investigated the synthesis and full characterization of a new set of fifteen pyrazole hybrids. All of these pyrazole derivatives bearing chalcone, thiazole and thiadiazole moieties were tested *in vitro* against PaCa-2, MCF-7 PC3 and BJ1. Three pyrazolyl-chalcone derivatives (4, 5, and 7) showed potent anti-cancer activity against PaCa-2 cells with IC_50_ values of 13.0, 31.5 and 24.9 μg mL^−1^, respectively, compared with doxorubicin (IC_50_, 28.3 μg mL^−1^). On the other hand, pyrazolyl-thiadiazole derivative 25 showed potent anti-cancer activity against PaCa-2 cells with IC_50_ = 5.5 μg mL^−1^ compared with doxorubicin (IC_50_, 28.3 μg mL^−1^), while compounds 23 and 25 showed potent anti-cancer activity against PC3 cells with IC_50_ values 26.1 and 11.8 μg mL^−1^, respectively, compared with doxorubicin (IC_50_, 23.8 μg mL^−1^). Moreover, the mechanism of action of the most active compounds against pancreatic cancer cells from the pyrazolyl-chalcone series was studied by investigating the gene expression, DNA fragmentation, and comet assay experiments using doxorubicin as a reference drug. These results indicated that positive control and compound 4 induced cell cycle arrest at the S and G2/M phases in the treated PaCa-2 cells. Finally, the structure–activity relationship between the structures of these compounds and their biological properties was discussed.

## Data availability

The data supporting this article have been included as part of the ESI.† Additional data are available upon request from the corresponding authors.

## Author contributions

Monica Kamel: chemical reactions, methodology, investigation. Farid Sroor: conceptualization, design, chemical reactions, methodology, writing – original draft, writing – review & editing, visualization, supervision, data curation, investigation. Mahmoud Hanafy: anti-cancer activity, flow cytometry. Karima Mahrous: gene expression, DNA damage, comet assay. Hamdi M. Hassaneen: supervision, investigation, and writing the original draft. All authors have seen and approved the submitted manuscript.

## Conflicts of interest

There are no financial or other relationships that might lead to a conflict of interest.

## Supplementary Material

RA-014-D4RA03005B-s001
