# Supplementary material for: Design, synthesis and potent anti-pancreatic cancer activity of new pyrazole derivatives bearing chalcone, thiazole and thiadiazole moieties: gene expression, DNA fragmentation, cell cycle arrest and SAR
Source: RSC Adv. 2024 Aug 27;14(37):26954–70. doi: 10.1039/d4ra03005b (PMC11348842; doi:10.1039/d4ra03005b)
Supplement: RA-014-D4RA03005B-s001 [file RA-014-D4RA03005B-s001.pdf]

Supplementary Information (SI) for RSC Advances.
This journal is © The Royal Society of Chemistry 2024

Synthesis and potent anti-pancreatic cancer activity of new pyrazole derivatives bearing chalcone, thiazol and thiadiazol moieties: Gene expression, DNA fragmentation and SAR

Monica G. Kamel[1], Farid M. Sroor[2,*], Mahmoud KH. Hanafy[3], Karima F. Mahrous[4], Hamdi M. Hassaneen[1,*]

1. Department of Chemistry, Faculty of Science, Cairo University, Giza, Egypt
2. Organometallic and Organometalloid Chemistry Department, National Research Centre, 12622 Cairo, Egypt
3. Bioassay-Cell Culture Laboratory, National Research Centre, 12622-Dokki, Egypt
4. Cell Biology Department, National Research Centre, 12622-Dokki, Egypt

Correspondence authors

Farid M Sroor, faridsroor@gmx.de, fm.sroor@nrc.sci.eg
Hamdi M. Hassaneen, hamdi_251@yahoo.com

MernaTalat-M2-CDC13-H1

Archive directory: /export/home/vnmr1/vnmrsys/data
Sample directory: DD5mm_test_12Mar2014-21:34:40

Pulse Sequence: s2pul
Solvent: CDC13
Temp. 30.0 C / 303.1 K
File: MernaTalat-M2-CDC13-H1
Mercury-300BB "NMR300"

Relax. delay 6.000 sec
Pulse 25.4 degrees
Acq. time 4.004 sec
Width 6600.7 Hz
10 repetitions
OBSERVE H1, 300.0673637 MHz
DATA PROCESSING
Line broadening 0.9 Hz
FT size 65536
Total time 58 min, 56 sec
Date: Aug 25 2021

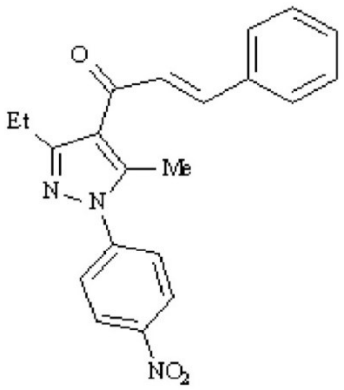

(4)

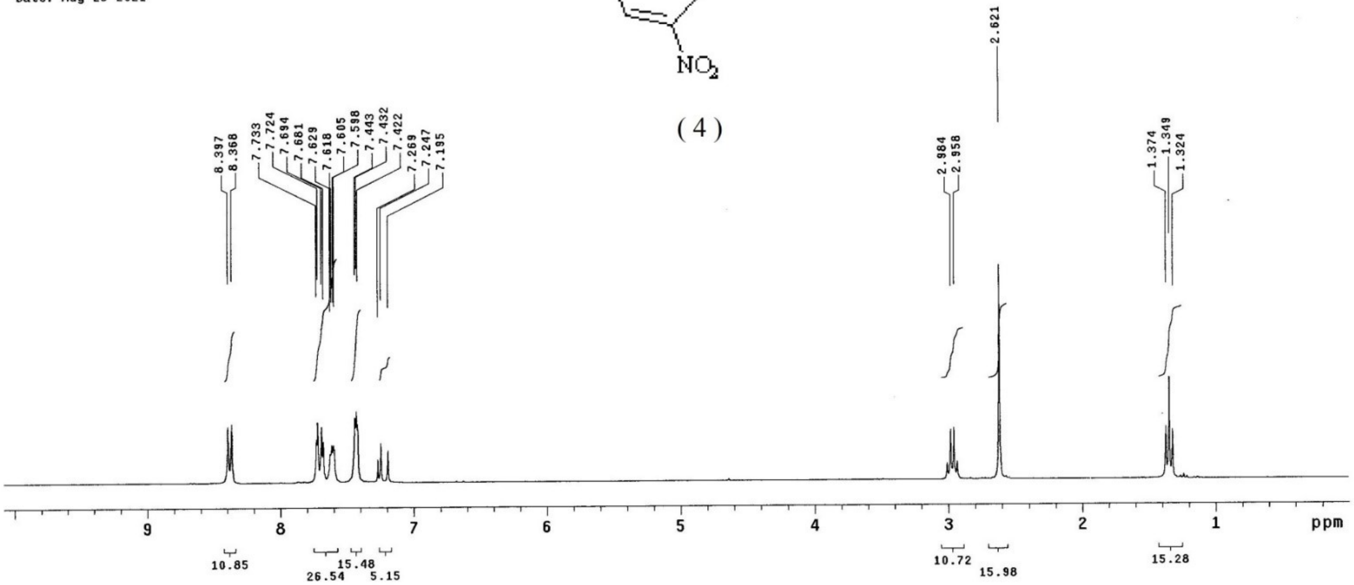

MernaTalaat-M2-CDCl3-C13

Archive directory: /export/home/vnmr1/vnmrsys/data
Sample directory: DD5mm_test_12Mar2014-21:34:40
File: PROTON

Pulse Sequence: s2pul

Solvent: CDCl3
Ambient temperature
Mercury-300BB "NMR300"

Pulse 45.0 degrees
Acq. time 1.707 sec
Width 18761.7 Hz
1360 repetitions
OBSERVE C13, 75.4520097 MHz
DECOUPLE H1, 300.0688576 MHz
Power 34 dB
continuously on
WALTZ-16 modulated
DATA PROCESSING
Line broadening 1.0 Hz
FT size 65536
Total time 24 hr, 53 min, 46 sec
Date: Aug 26 2021

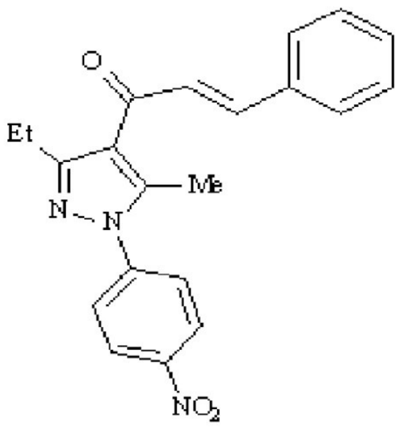

(4)

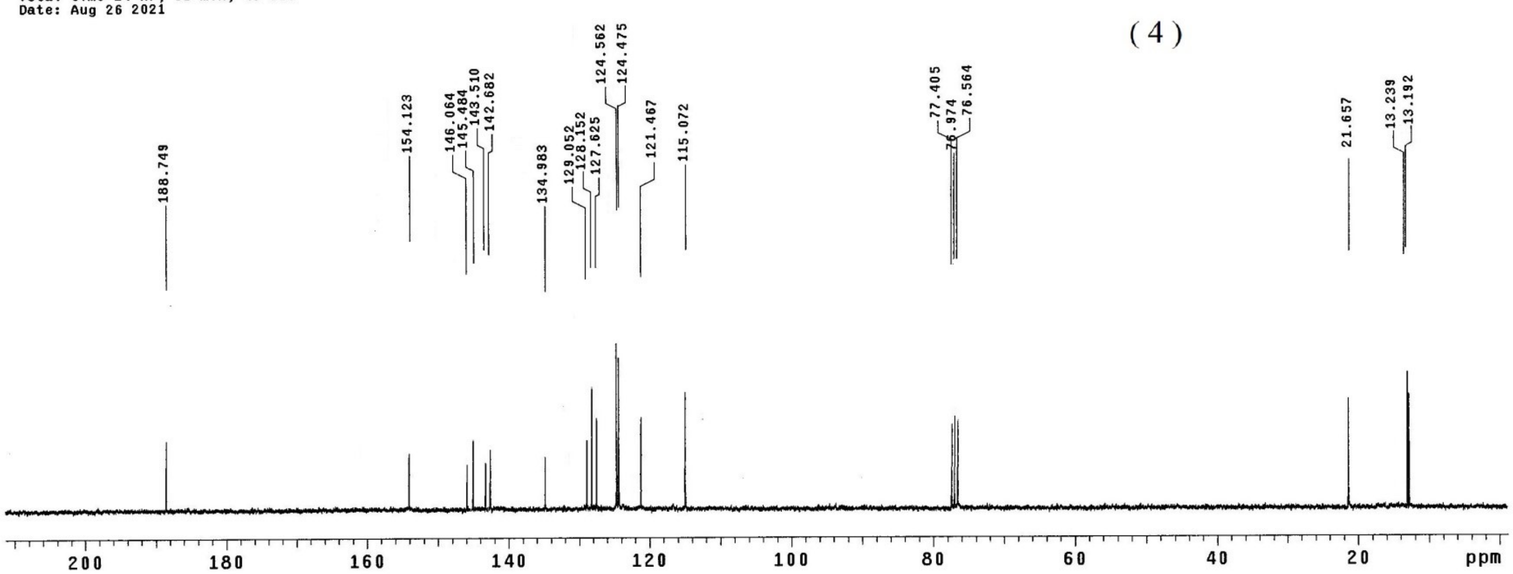

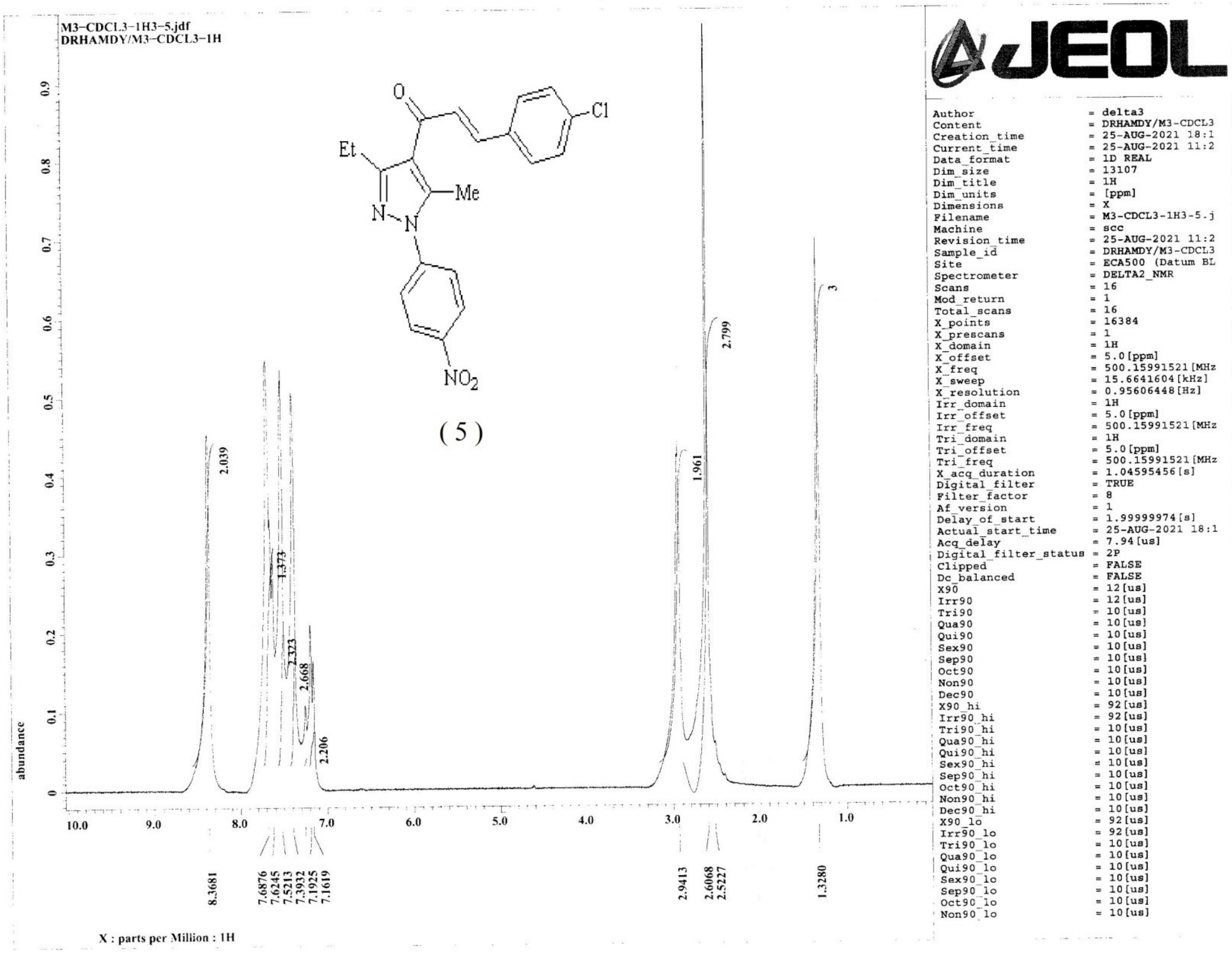

Author	= delta3
Content	= DRHAMDY/M3-CDCL3
Creation_time	= 25-AUG-2021 18:1
Current_time	= 25-AUG-2021 11:2
Data_format	= 1D REAL
Dim_size	= 13107
Dim_title	= 1H
Dim_units	= [ppm]
Dimensions	= X
Filename	= M3-CDCL3-1H3-5.j
Machine	= scc
Revision_time	= 25-AUG-2021 11:2
Sample_id	= DRHAMDY/M3-CDCL3
Site	= ECA500 (Datum BL
Spectrometer	= DELTA2_NMR
Scans	= 16
Mod_return	= 1
Total_scans	= 16
X_points	= 16384
X_prescans	= 1
X_domain	= 1H
X_offset	= 5.0[ppm]
X_freq	= 500.15991521[MHz
X_sweep	= 15.6641604[kHz]
X_resolution	= 0.95606448[Hz]
Irr_domain	= 1H
Irr_offset	= 5.0[ppm]
Irr_freq	= 500.15991521[MHz
Tri_domain	= 1H
Tri_offset	= 5.0[ppm]
Tri_freq	= 500.15991521[MHz
X_acq_duration	= 1.04595456[s]
Digital_filter	= TRUE
Filter_factor	= 8
Af_version	= 1
Delay_of_start	= 1.99999974[s]
Actual_start_time	= 25-AUG-2021 18:1
Acq_delay	= 7.94[us]
Digital_filter_status	= 2P
Clipped	= FALSE
Dc_balanced	= FALSE
X90	= 12[us]
Irr90	= 12[us]
Tri90	= 10[us]
Qua90	= 10[us]
Qui90	= 10[us]
Sex90	= 10[us]
Sep90	= 10[us]
Oct90	= 10[us]
Non90	= 10[us]
Dec90	= 10[us]
X90_hi	= 92[us]
Irr90_hi	= 92[us]
Tri90_hi	= 10[us]
Qua90_hi	= 10[us]
Qui90_hi	= 10[us]
Sex90_hi	= 10[us]
Sep90_hi	= 10[us]
Oct90_hi	= 10[us]
Non90_hi	= 10[us]
Dec90_hi	= 10[us]
X90_lo	= 92[us]
Irr90_lo	= 92[us]
Tri90_lo	= 10[us]
Qua90_lo	= 10[us]
Qui90_lo	= 10[us]
Sex90_lo	= 10[us]
Sep90_lo	= 10[us]
Oct90_lo	= 10[us]
Non90_lo	= 10[us]

HamdyHasaneen-M3-CDCl3-C13

Archive directory: /export/home/vnmr1/vnmrsys/data
Sample directory: DD5mm_test_12Mar2014-21:34:40
File: PROTON

Pulse Sequence: s2pul

Solvent: CDCl3
Ambient temperature
Mercury-300BB "NMR300"

Pulse 45.0 degrees
Acq. time 1.707 sec
Width 18761.7 Hz
1752 repetitions
OBSERVE C13, 75.4520074 MHz
DECOUPLE H1, 300.0688576 MHz
Power 34 dB
continuously on
WALTZ-16 modulated
DATA PROCESSING
Line broadening 1.0 Hz
FT size 65536
Total time 31 hr, 7 min, 12 sec
Date: Dec 7 2021

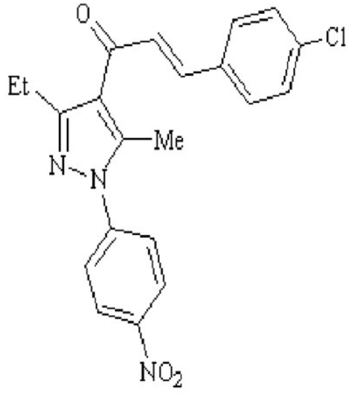

(5)

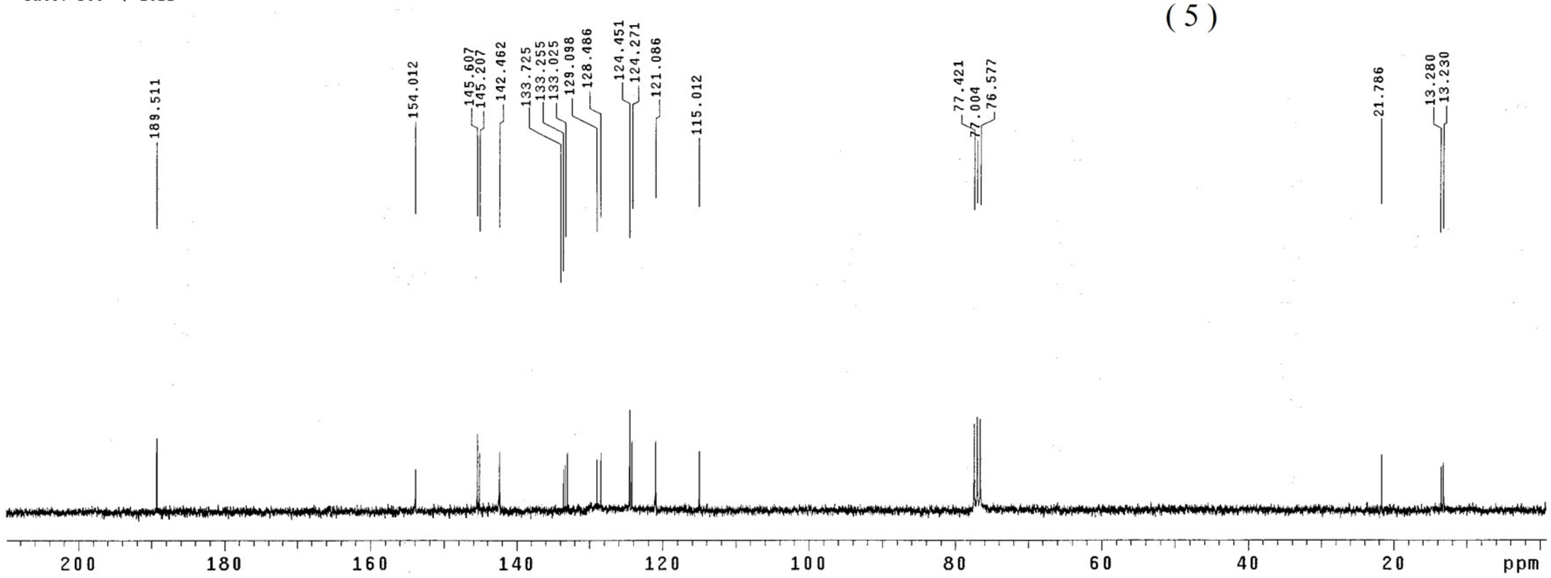

HamdyHasaneen-M13-CDCl3-H1

Archive directory: /export/home/vnmr1/vnmrsys/data
Sample directory: DD5mm_test_12Mar2014-21:34:40

Pulse Sequence: s2pul

Solvent: CDCl3
Temp. 30.0 C / 303.1 K
File: HamdyHasaneen-M13-CDCl3-H1
Mercury-300BB "NMR300"

Relax. delay 6.000 sec
Pulse 25.4 degrees
Acq. time 4.004 sec
Width 6600.7 Hz
7 repetitions
OBSERVE H1, 300.0673637 MHz
DATA PROCESSING
Line broadening 0.9 Hz
FT size 65536
Total time 58 min, 56 sec
Date: Nov 29 2023

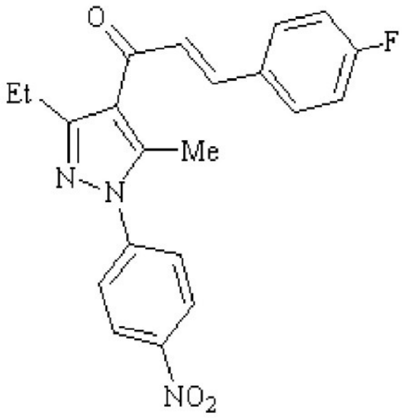

(6)

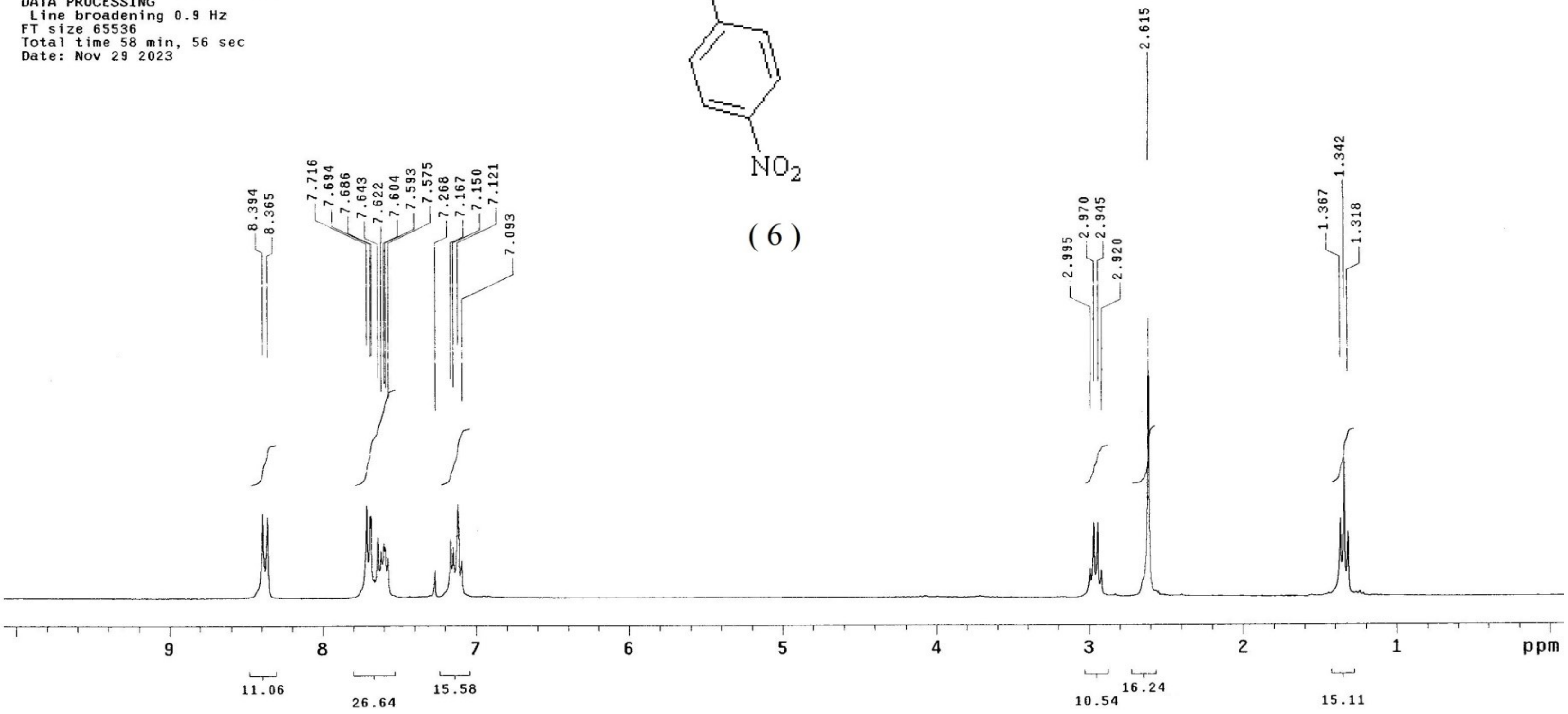

HamdyHasaneen-M13-CDC13-C13

Archive directory: /export/home/vnmr1/vnmrsys/data
Sample directory: DD5mm_test_12Mar2014-21:34:40
File: PROTON

Pulse Sequence: s2pul
Solvent: CDC13
Ambient temperature
Mercury-300BB "NMR300"

Pulse 45.0 degrees
Acq. time 1.707 sec
Width 18761.7 Hz
2088 repetitions
OBSERVE C13, 75.4520097 MHz
DECOUPLE H1, 300.0688576 MHz
Power 34 dB
continuously on
WALTZ-16 modulated
DATA PROCESSING
Line broadening 1.0 Hz
FT size 65536
Total time 24 hr, 53 min, 46 sec
Date: Nov 30 2023

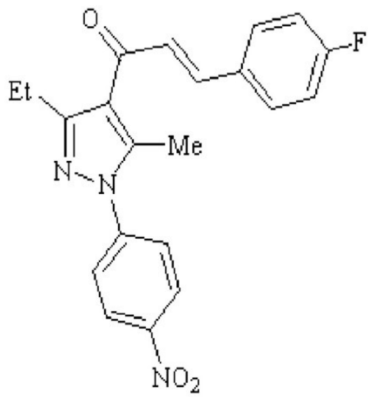

(6)

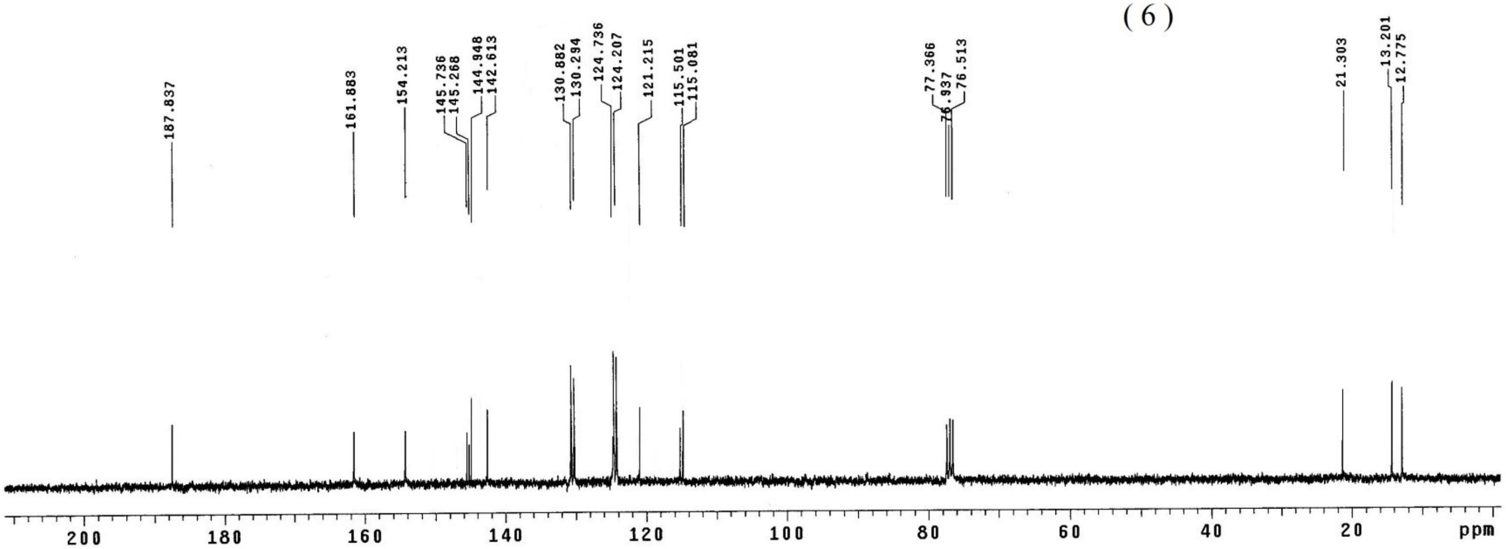

MohammadAbdulMoaty-M4-CDCl3-C13

Archive directory: /export/home/vnmr1/vnmrsys/data
Sample directory: DD5mm_test_12Mar2014-21:34:40
File: PROTON

Pulse Sequence: s2pul

Solvent: CDCl3
Ambient temperature
Mercury-300BB "NMR300"

Pulse 45.0 degrees
Acq. time 1.707 sec
Width 18761.7 Hz
1456 repetitions
OBSERVE C13, 75.4520091 MHz
DECOUPLE H1, 300.0688576 MHz
Power 34 dB
continuously on
WALTZ-16 modulated
DATA PROCESSING
Line broadening 1.0 Hz
FT size 65536
Total time 24 hr, 53 min, 46 sec
Date: Jan 13 2021

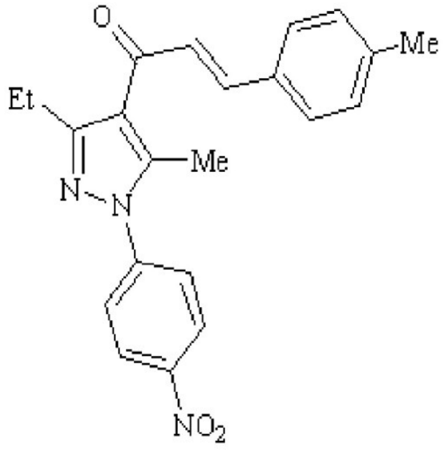

(7)

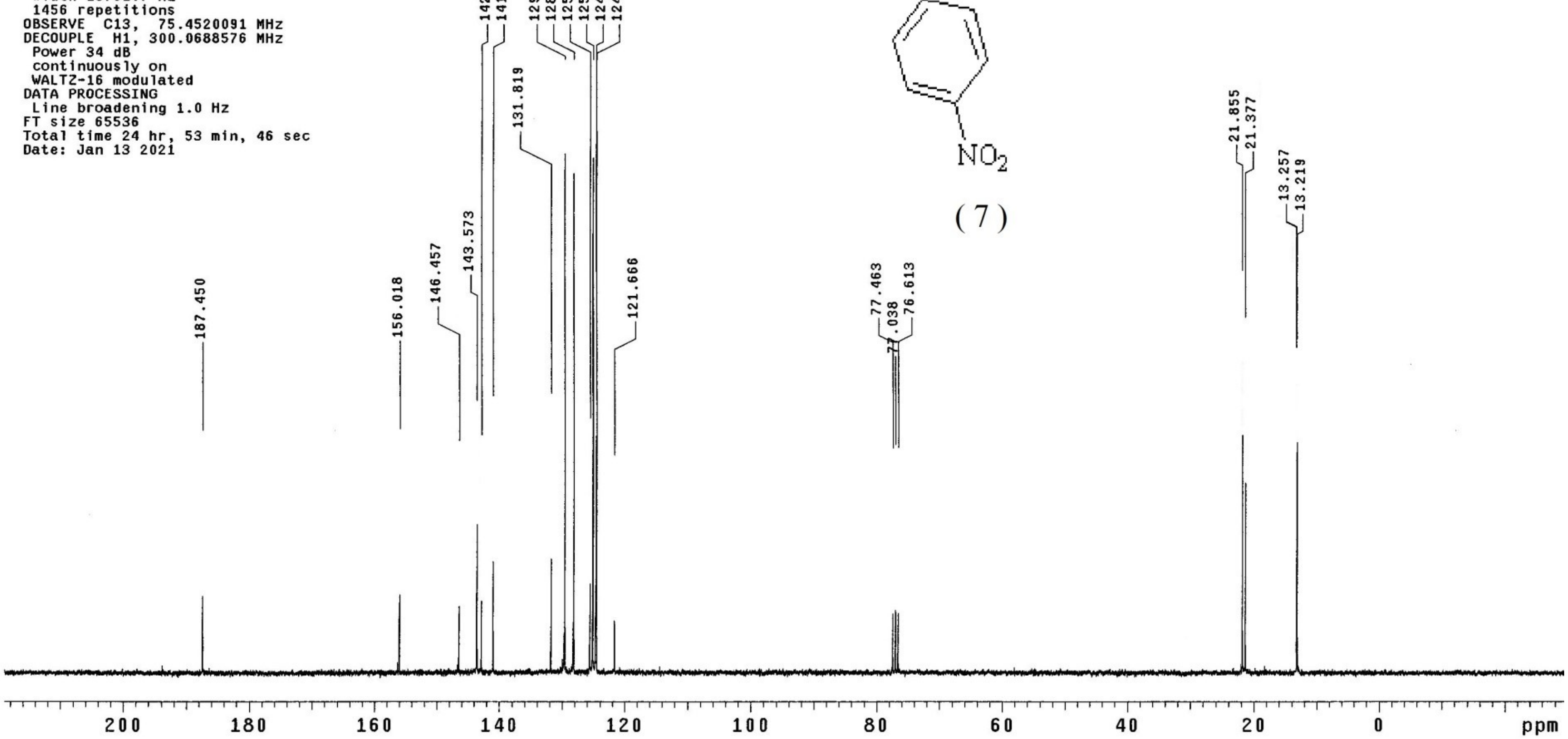

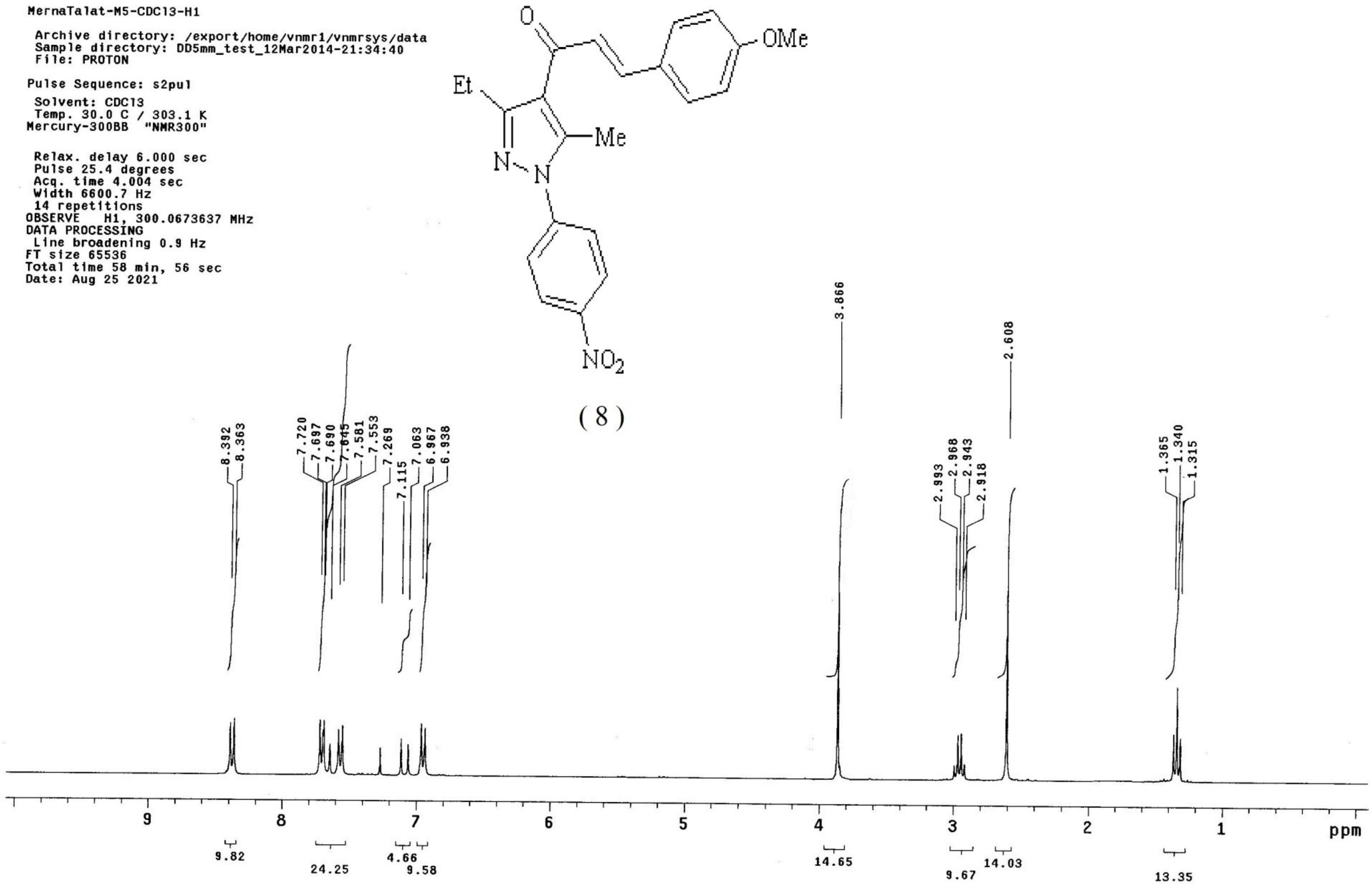
MernaTalat-M5-CDCl3-H1
Archive directory: /export/home/vnmr1/vnmrsys/data
Sample directory: DD5mm_test_12Mar2014-21:34:40
File: PROTON
Pulse Sequence: s2pul
Solvent: CDCl3
Temp. 30.0 C / 303.1 K
Mercury-300BB "NMR300"
Relax. delay 6.000 sec
Pulse 25.4 degrees
Acq. time 4.004 sec
Width 6600.7 Hz
14 repetitions
OBSERVE H1, 300.0673637 MHz
DATA PROCESSING
Line broadening 0.9 Hz
FT size 65536
Total time 58 min, 56 sec
Date: Aug 25 2021
O
OMe
Et
Me
N
N
NO2
(8)
8.392
8.363
7.720
7.697
7.690
7.645
7.581
7.553
7.269
7.115
7.063
6.967
6.938
3.866
2.608
2.993
2.968
2.943
2.918
1.365
1.340
1.315
9
8
7
6
5
4
3
2
1
ppm
9.82
24.25
4.66
9.58
14.65
9.67
14.03
13.35

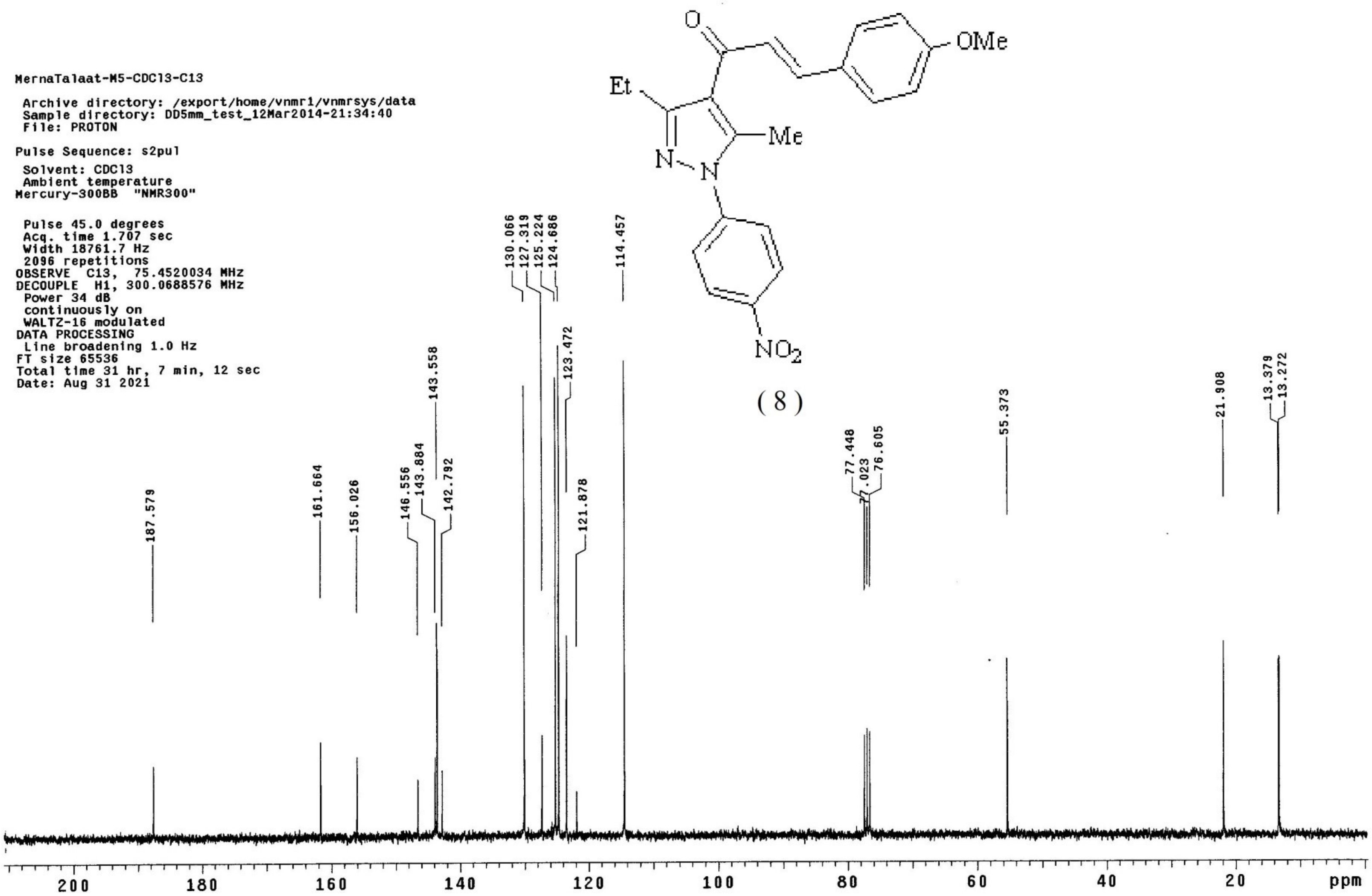
MernaTalaat-M5-CDCl3-C13
Archive directory: /export/home/vnmr1/vnmrsys/data
Sample directory: DD5mm_test_12Mar2014-21:34:40
File: PROTON
Pulse Sequence: s2pul
Solvent: CDCl3
Ambient temperature
Mercury-300BB "NMR300"
Pulse 45.0 degrees
Acq. time 1.707 sec
Width 18761.7 Hz
2096 repetitions
OBSERVE C13, 75.4520034 MHz
DECOUPLE H1, 300.0688576 MHz
Power 34 dB
continuously on
WALTZ-16 modulated
DATA PROCESSING
Line broadening 1.0 Hz
FT size 65536
Total time 31 hr, 7 min, 12 sec
Date: Aug 31 2021
O
OMe
Et
Me
N
N
NO2
(8)
187.579
161.664
156.026
146.556
143.884
143.558
142.792
130.066
127.319
125.224
124.686
123.472
121.878
114.457
77.448
77.023
76.605
55.373
21.908
13.379
13.272
200
180
160
140
120
100
80
60
40
20
ppm

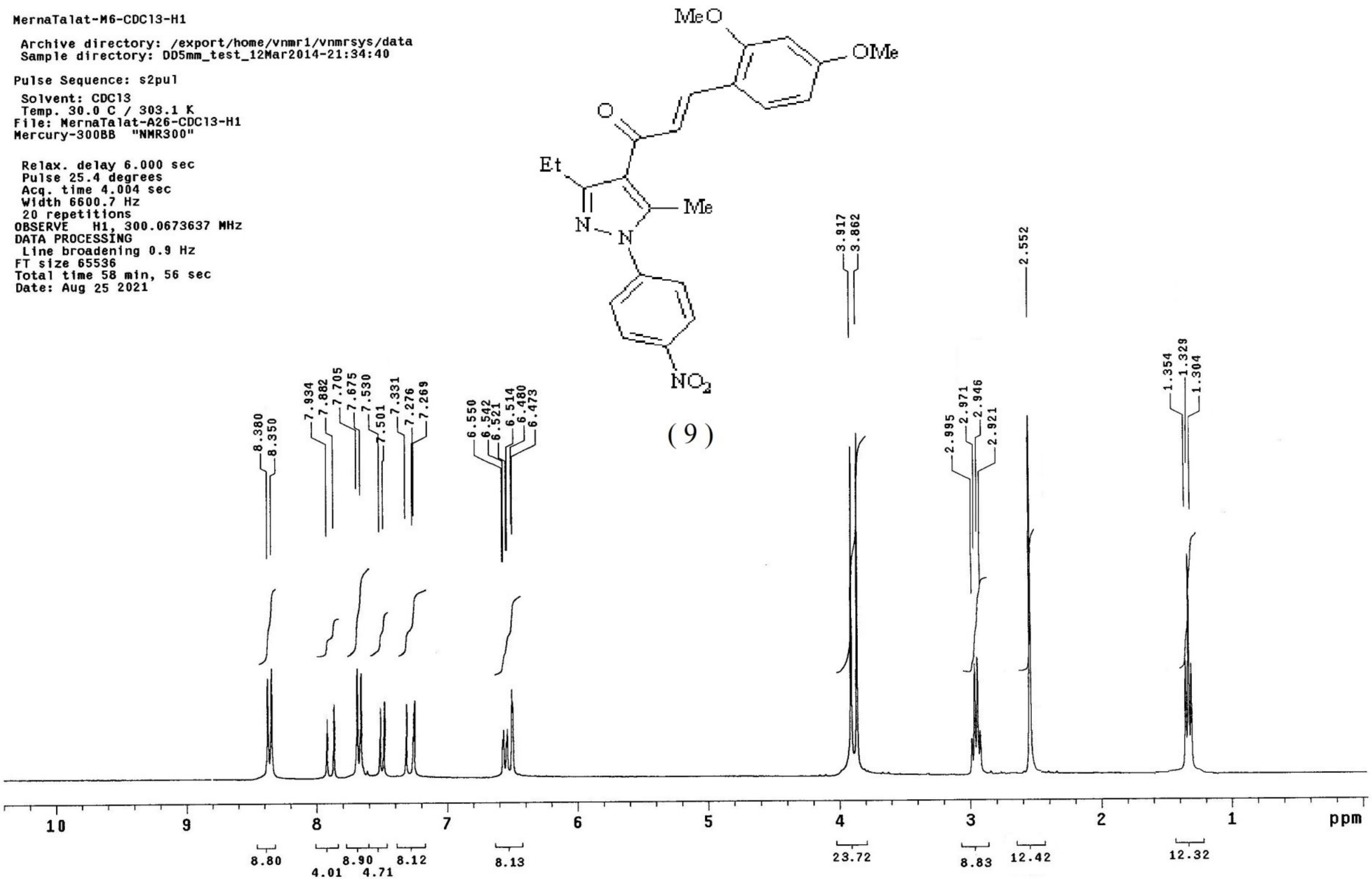
MernaTalat-M6-CDCl3-H1
Archive directory: /export/home/vnmr1/vnmrsys/data
Sample directory: DD5mm_test_12Mar2014-21:34:40
Pulse Sequence: s2pul
Solvent: CDCl3
Temp. 30.0 C / 303.1 K
File: MernaTalat-A26-CDCl3-H1
Mercury-300BB "NMR300"
Relax. delay 6.000 sec
Pulse 25.4 degrees
Acq. time 4.004 sec
Width 6600.7 Hz
20 repetitions
OBSERVE H1, 300.0673637 MHz
DATA PROCESSING
Line broadening 0.9 Hz
FT size 65536
Total time 58 min, 56 sec
Date: Aug 25 2021
MeO
OMe
O
Et
Me
N
N
NO2
8.380
8.350
7.934
7.882
7.705
7.675
7.530
7.501
7.331
7.276
7.269
6.550
6.542
6.521
6.514
6.480
6.473
3.917
3.862
2.995
2.971
2.946
2.921
2.552
1.354
1.329
1.304
10 9 8 7 6 5 4 3 2 1 ppm
8.80
4.01
8.90
4.71
8.12
8.13
23.72
8.83
12.42
12.32

(9)

MernaTalaat-M6-CDCl3-C13

Archive directory: /export/home/vnmr1/vnmrsys/data
Sample directory: DD5mm_test_12Mar2014-21:34:40
File: PROTON

Pulse Sequence: s2pul

Solvent: CDCl3
Ambient temperature
Mercury-300BB "NMR300"

Pulse 45.0 degrees
Acq. time 1.707 sec
Width 18761.7 Hz
1360 repetitions
OBSERVE C13, 75.4520097 MHz
DECOUPLE H1, 300.0688576 MHz
Power 34 dB
continuously on
WALTZ-16 modulated
DATA PROCESSING
Line broadening 1.0 Hz
FT size 65536
Total time 24 hr, 53 min, 46 sec
Date: Aug 26 2021

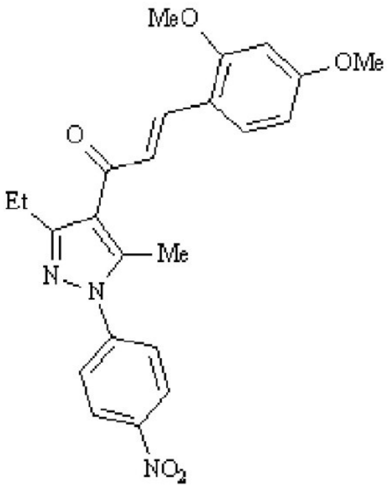

(9)

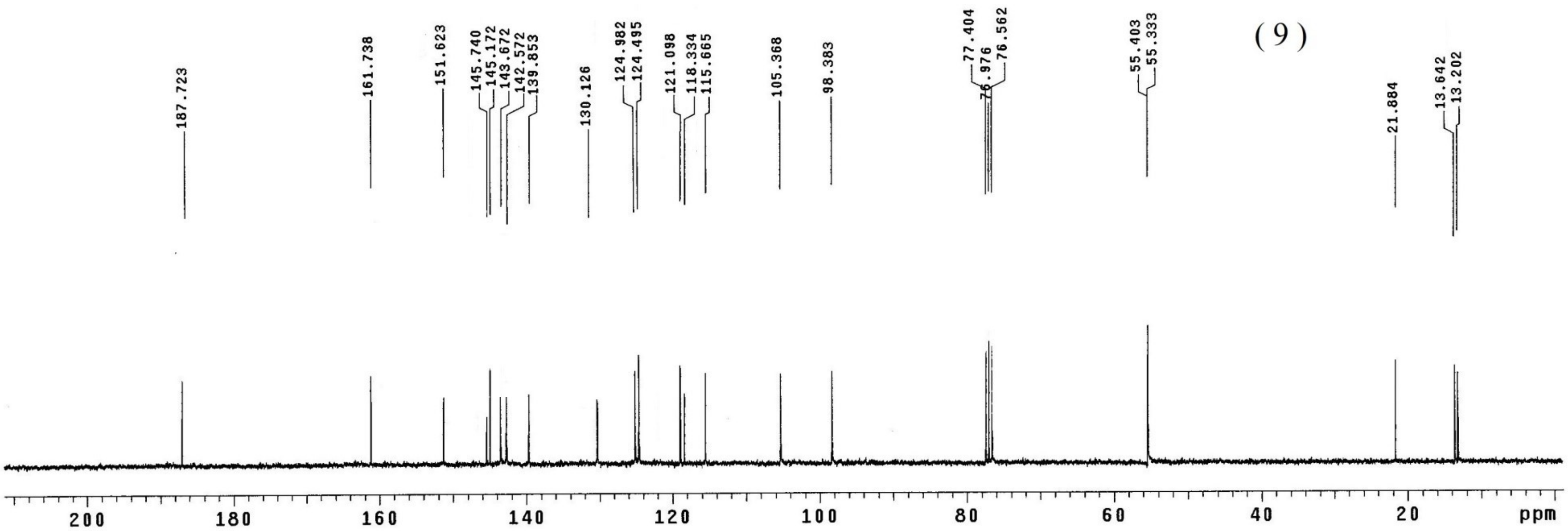

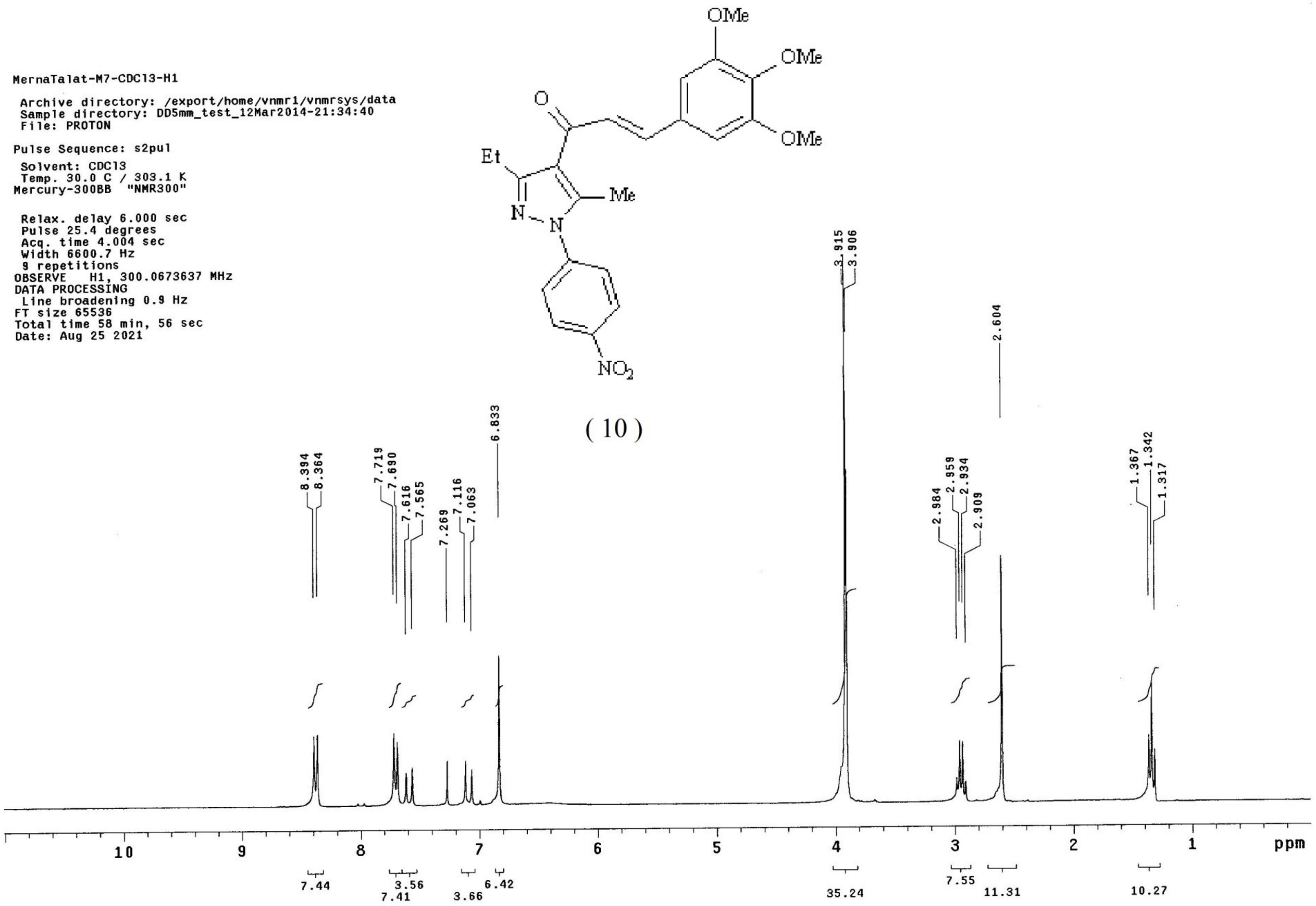
MernaTalat-M7-CDCl3-H1
Archive directory: /export/home/vnmr1/vnmrsys/data
Sample directory: DD5mm_test_12Mar2014-21:34:40
File: PROTON
Pulse Sequence: s2pul
Solvent: CDCl3
Temp. 30.0 C / 303.1 K
Mercury-300BB "NMR300"
Relax. delay 6.000 sec
Pulse 25.4 degrees
Acq. time 4.004 sec
Width 6600.7 Hz
9 repetitions
OBSERVE H1, 300.0673637 MHz
DATA PROCESSING
Line broadening 0.9 Hz
FT size 65536
Total time 58 min, 56 sec
Date: Aug 25 2021
OMe
OMe
OMe
O
Et
Me
N
N
NO2
8.394
8.364
7.719
7.690
7.616
7.565
7.269
7.116
7.063
6.833
3.915
3.906
2.984
2.959
2.934
2.909
2.604
1.367
1.342
1.317
ppm
7.44
3.56
7.41
3.66
6.42
35.24
7.55
11.31
10.27
(10)

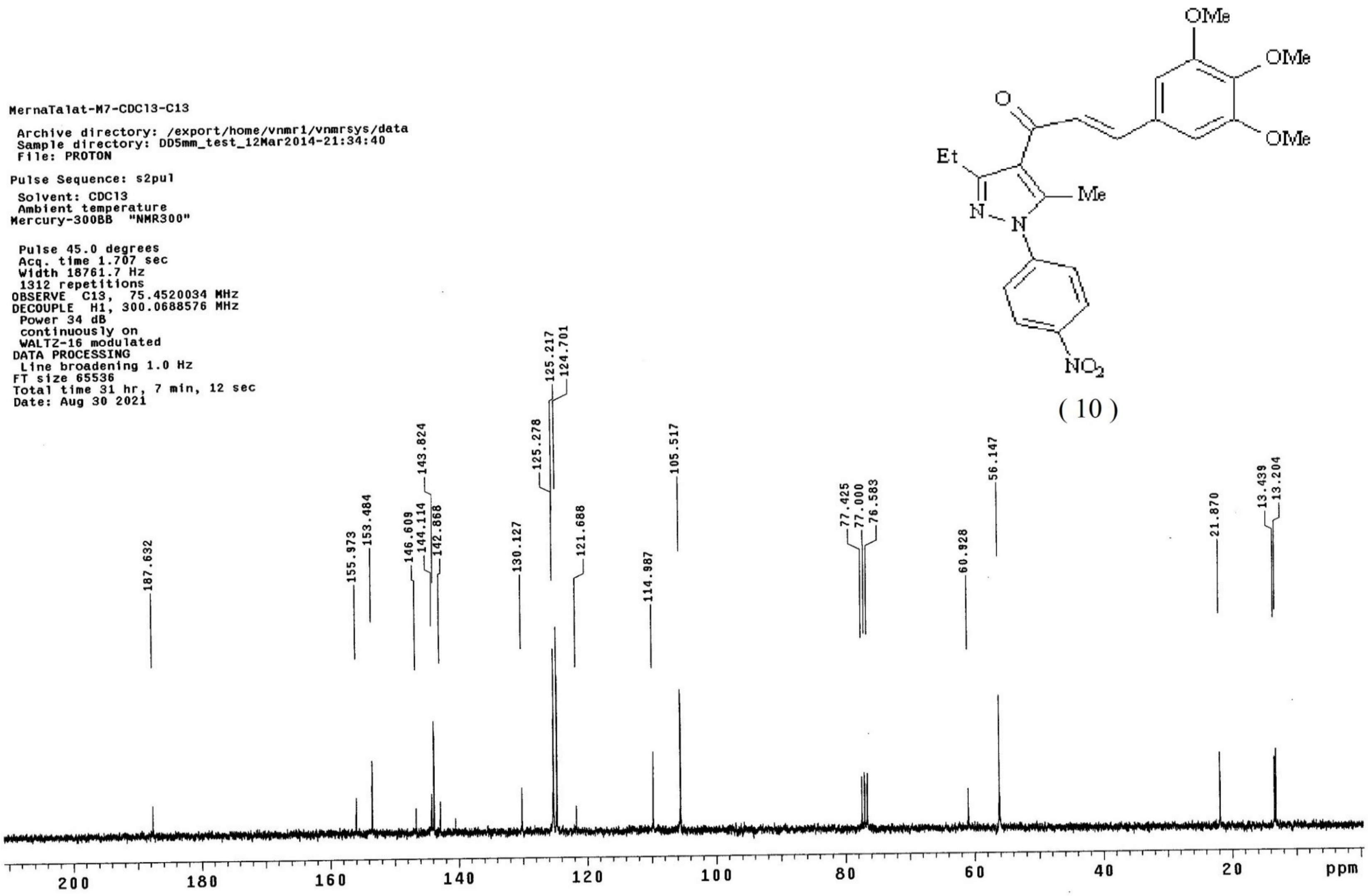
MernaTalat-M7-CDCl3-C13
Archive directory: /export/home/vnmr1/vnmrsys/data
Sample directory: DD5mm_test_12Mar2014-21:34:40
File: PROTON
Pulse Sequence: s2pul
Solvent: CDCl3
Ambient temperature
Mercury-300BB "NMR300"
Pulse 45.0 degrees
Acq. time 1.707 sec
Width 18761.7 Hz
1312 repetitions
OBSERVE C13, 75.4520034 MHz
DECOUPLE H1, 300.0688576 MHz
Power 34 dB
continuously on
WALTZ-16 modulated
DATA PROCESSING
Line broadening 1.0 Hz
FT size 65536
Total time 31 hr, 7 min, 12 sec
Date: Aug 30 2021
OMe
OMe
OMe
O
Et
Me
N
N
NO2
(10)
187.632
155.973
153.484
146.609
144.114
143.824
142.868
130.127
125.278
125.217
124.701
121.688
114.987
105.517
77.425
77.000
76.583
60.928
56.147
21.870
13.439
13.204
200
180
160
140
120
100
80
60
40
20
ppm

MernaTalat-M9-CDCl3-H1

Archive directory: /export/home/vnmr1/vnmrsys/data
Sample directory: DD5mm_test_12Mar2014-21:34:40
File: PROTON

Pulse Sequence: s2pul

Solvent: CDCl3
Temp. 30.0 C / 303.1 K
Mercury-300BB "NMR300"

Relax. delay 6.000 sec
Pulse 25.4 degrees
Acq. time 4.004 sec
Width 6600.7 Hz
15 repetitions
OBSERVE H1, 300.0673637 MHz
DATA PROCESSING
Line broadening 0.9 Hz
FT size 65536
Total time 58 min, 56 sec
Date: Aug 25 2021

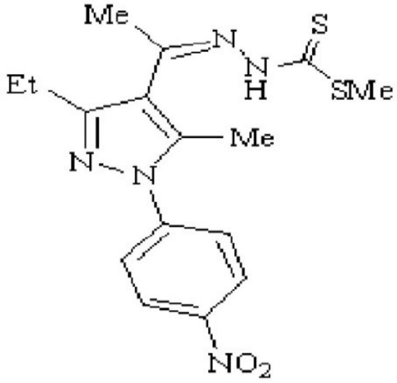

(13)

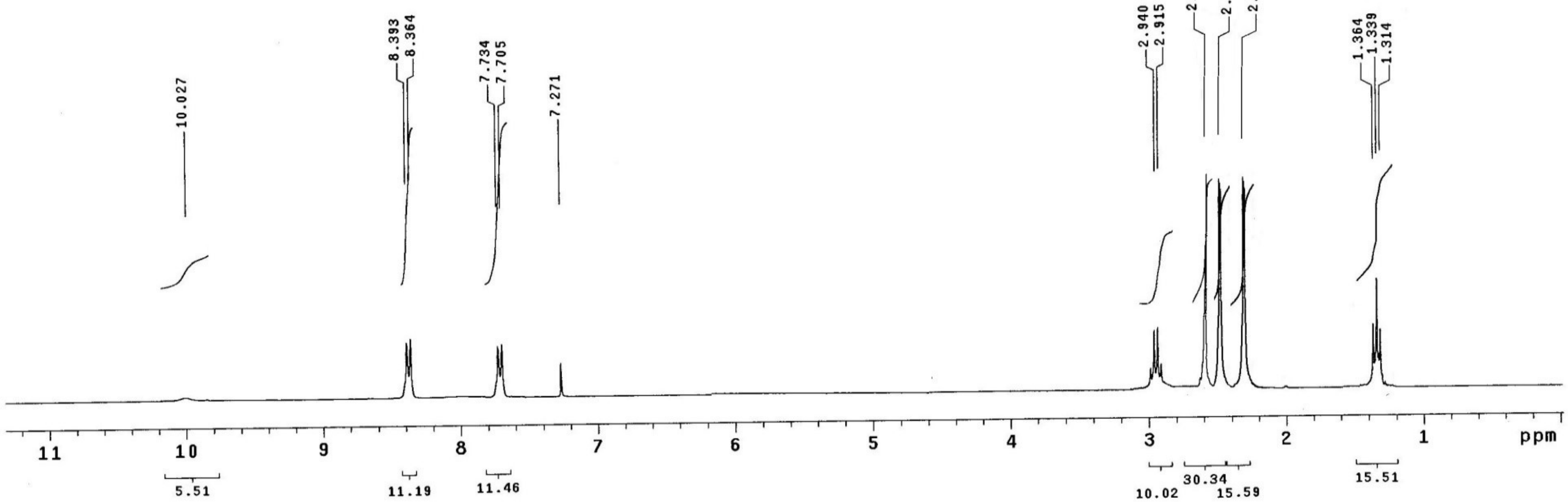

MernaTalaat-M9-CDCl3-C13

Archive directory: /export/home/vnmr1/vnmrsys/data
Sample directory: DD5mm_test_12Mar2014-21:34:40
File: PROTON

Pulse Sequence: s2pul

Solvent: DMSO
Ambient temperature
Mercury-300BB "NMR300"

Pulse 45.0 degrees
Acq. time 1.707 sec
Width 18761.7 Hz
1160 repetitions
OBSERVE C13, 75.4523880 MHz
DECOUPLE H1, 300.0702830 MHz
Power 34 dB
continuously on
WALTZ-16 modulated
DATA PROCESSING
Line broadening 1.0 Hz
FT size 65536
Total time 31 hr, 7 min, 12 sec
Date: Oct 13 2021

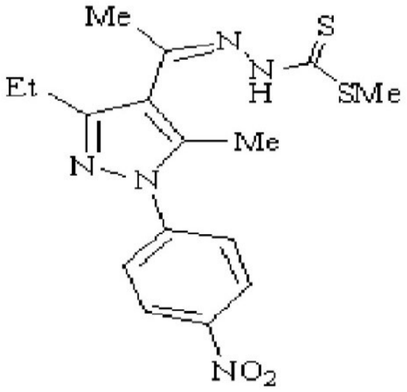

(13)

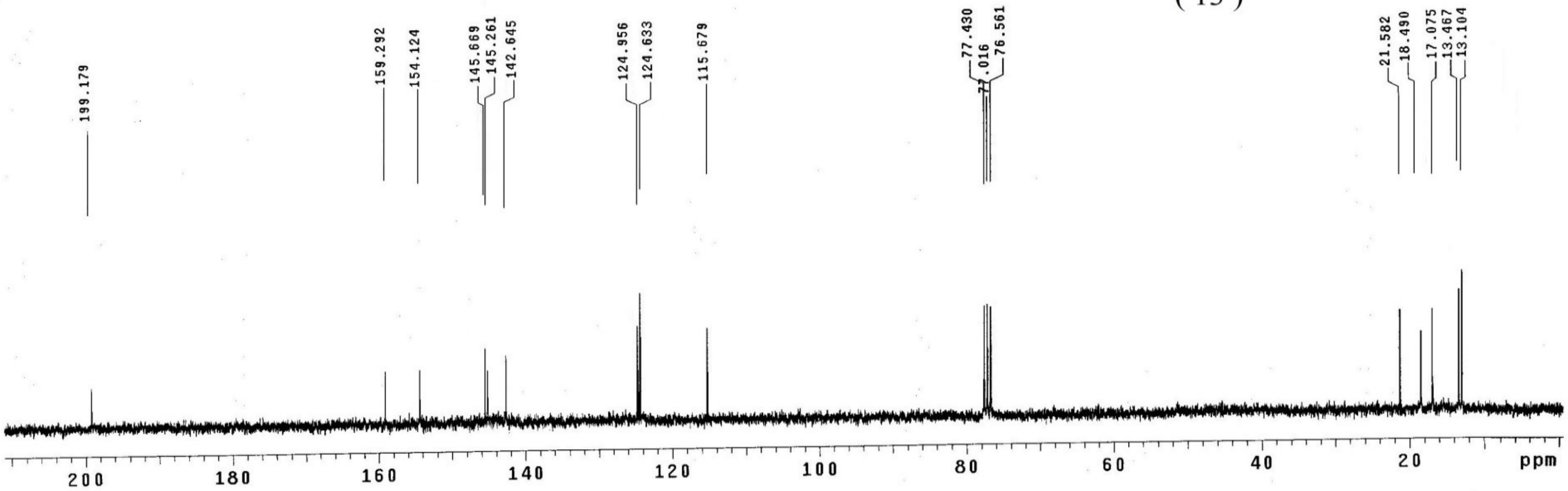

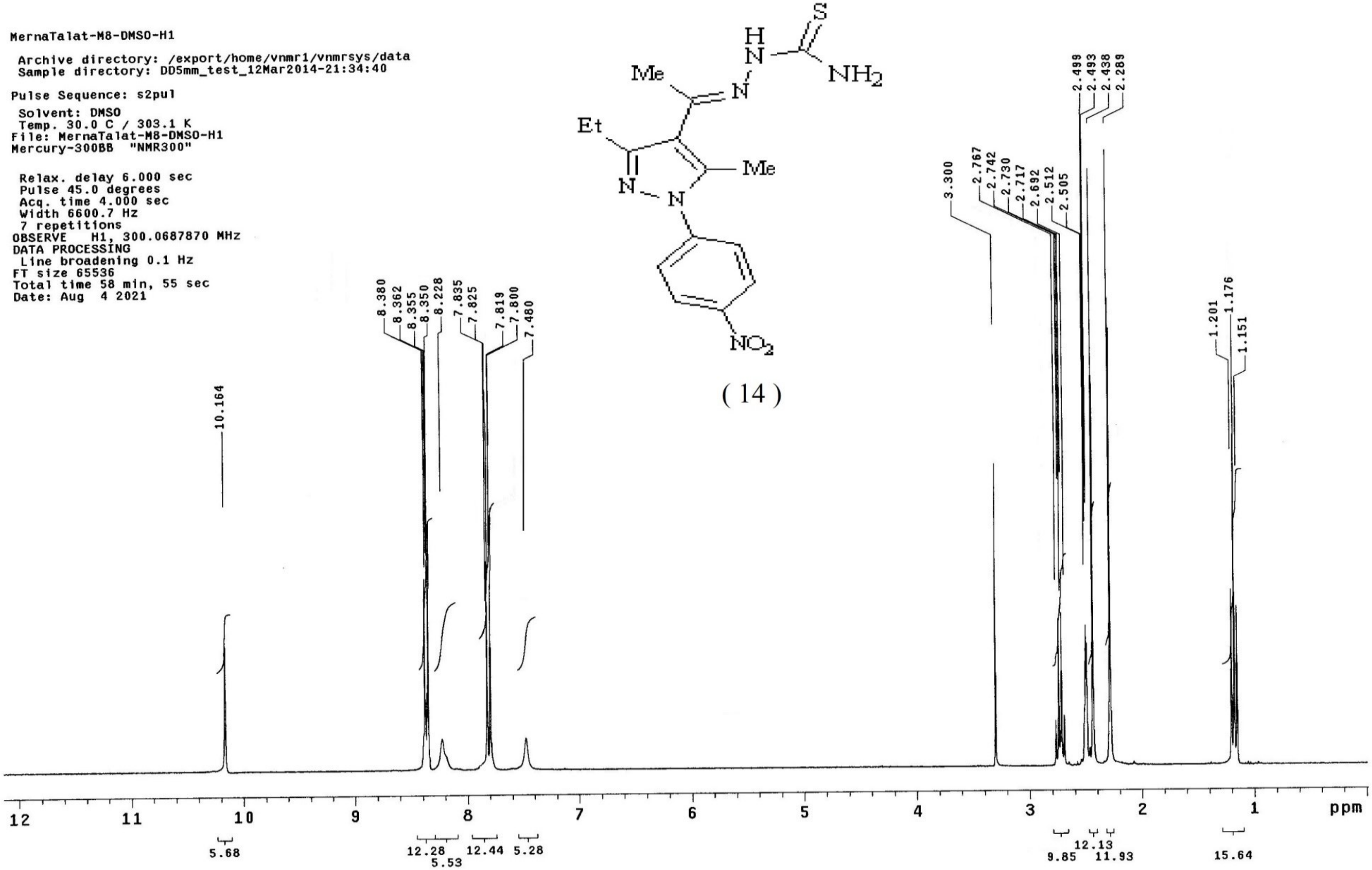
MernaTalat-M8-DMSO-H1
Archive directory: /export/home/vnmr1/vnmrsys/data
Sample directory: DD5mm_test_12Mar2014-21:34:40
Pulse Sequence: s2pul
Solvent: DMSO
Temp. 30.0 C / 303.1 K
File: MernaTalat-M8-DMSO-H1
Mercury-300BB "NMR300"
Relax. delay 6.000 sec
Pulse 45.0 degrees
Acq. time 4.000 sec
Width 6600.7 Hz
7 repetitions
OBSERVE H1, 300.0687870 MHz
DATA PROCESSING
Line broadening 0.1 Hz
FT size 65536
Total time 58 min, 55 sec
Date: Aug 4 2021
Me
Et
Me
NH2
NO2
(14)
10.164
8.380
8.362
8.355
8.350
8.228
7.835
7.825
7.819
7.800
7.480
3.300
2.767
2.742
2.730
2.717
2.692
2.512
2.505
2.499
2.493
2.438
2.289
1.201
1.176
1.151
12 11 10 9 8 7 6 5 4 3 2 1 ppm
5.68
12.28
5.53
12.44
5.28
9.85
12.13
11.93
15.64

MernaTalaat-M8-DMSO-C13

Archive directory: /export/home/vnmr1/vnmrsys/data
Sample directory: DD5mm_test_12Mar2014-21:34:40
File: PROTON

Pulse Sequence: s2pul

Solvent: DMSO
Ambient temperature
Mercury-300BB "NMR300"

Pulse 45.0 degrees
Acq. time 1.707 sec
Width 18761.7 Hz
3456 repetitions
OBSERVE C13, 75.4523885 MHz
DECOUPLE H1, 300.0702830 MHz
Power 34 dB
continuously on
WALTZ-16 modulated
DATA PROCESSING
Line broadening 1.0 Hz
FT size 65536
Total time 31 hr, 7 min, 12 sec
Date: Aug 5 2021

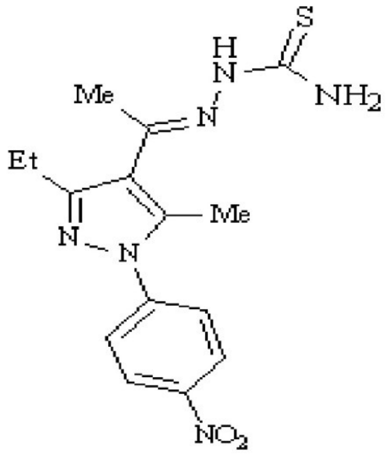

(14)

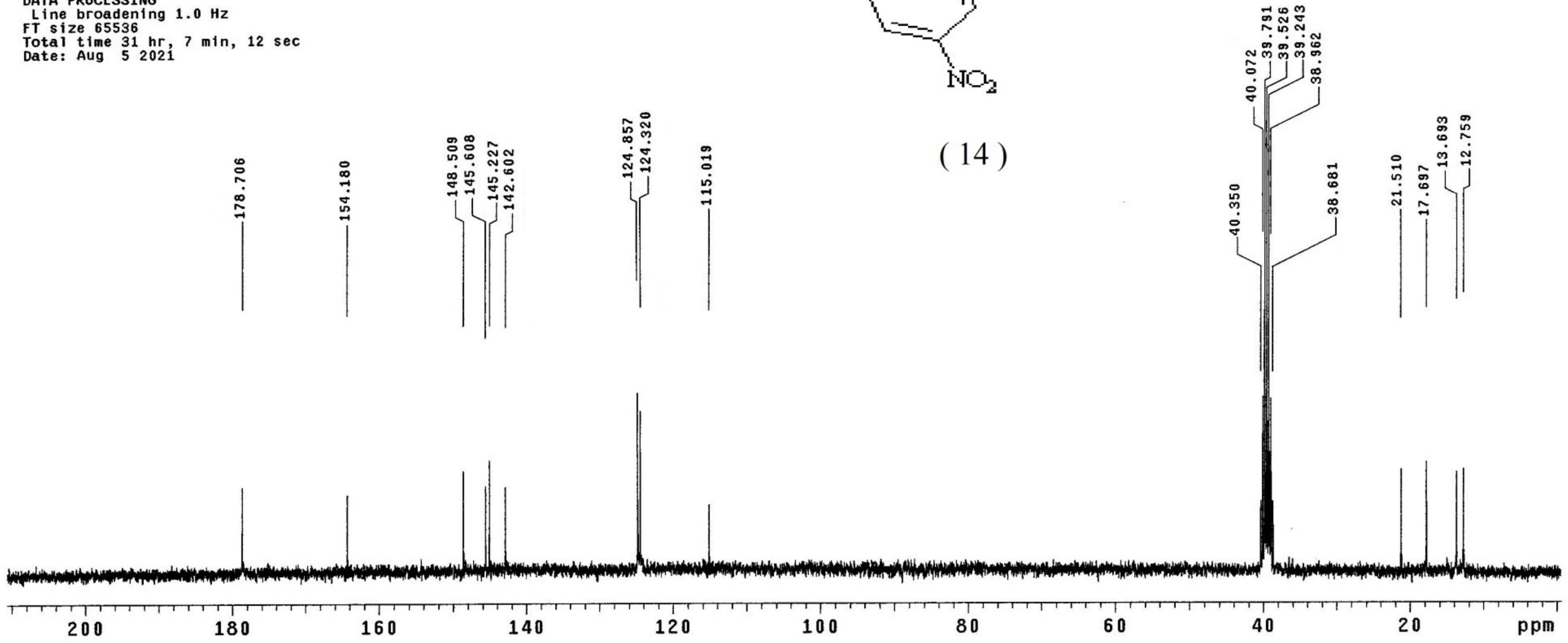

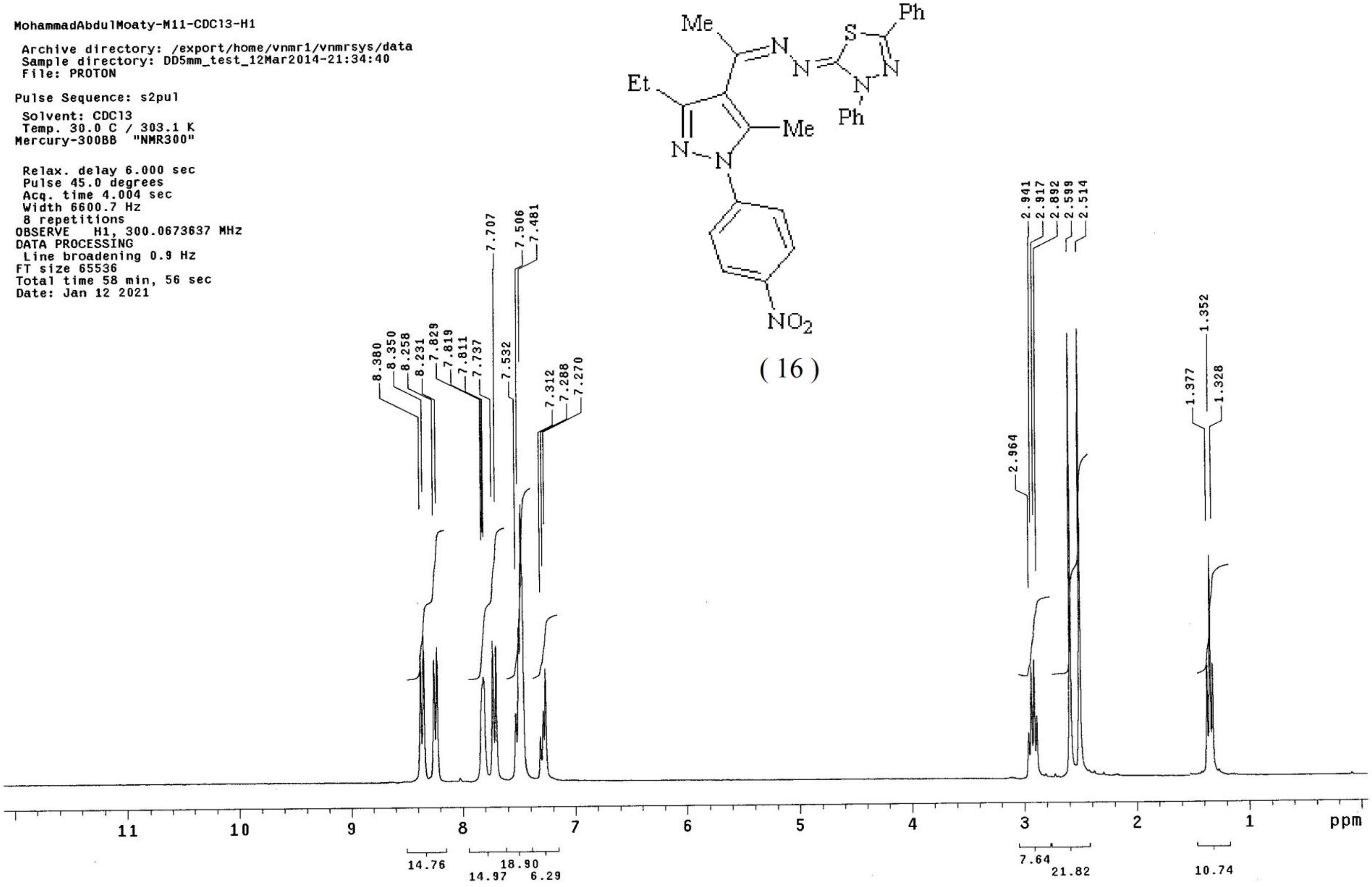
MohammadAbdulMoaty-M11-CDCl3-H1
Archive directory: /export/home/vnmr1/vnmrsys/data
Sample directory: DD5mm_test_12Mar2014-21:34:40
File: PROTON
Pulse Sequence: s2pul
Solvent: CDCl3
Temp. 30.0 C / 303.1 K
Mercury-300BB "NMR300"
Relax. delay 6.000 sec
Pulse 45.0 degrees
Acq. time 4.004 sec
Width 6600.7 Hz
8 repetitions
OBSERVE H1, 300.0673637 MHz
DATA PROCESSING
Line broadening 0.9 Hz
FT size 65536
Total time 58 min, 56 sec
Date: Jan 12 2021
Me
Ph
N
N
S
N
N
Et
N
Ph
Me
N
N
NO2
(16)
8.380
8.350
8.258
8.231
7.829
7.819
7.811
7.737
7.707
7.532
7.506
7.481
7.312
7.288
7.270
2.964
2.941
2.917
2.892
2.599
2.514
1.377
1.352
1.328
11
10
9
8
7
6
5
4
3
2
1
ppm
14.76
14.97
18.90
6.29
7.64
21.82
10.74

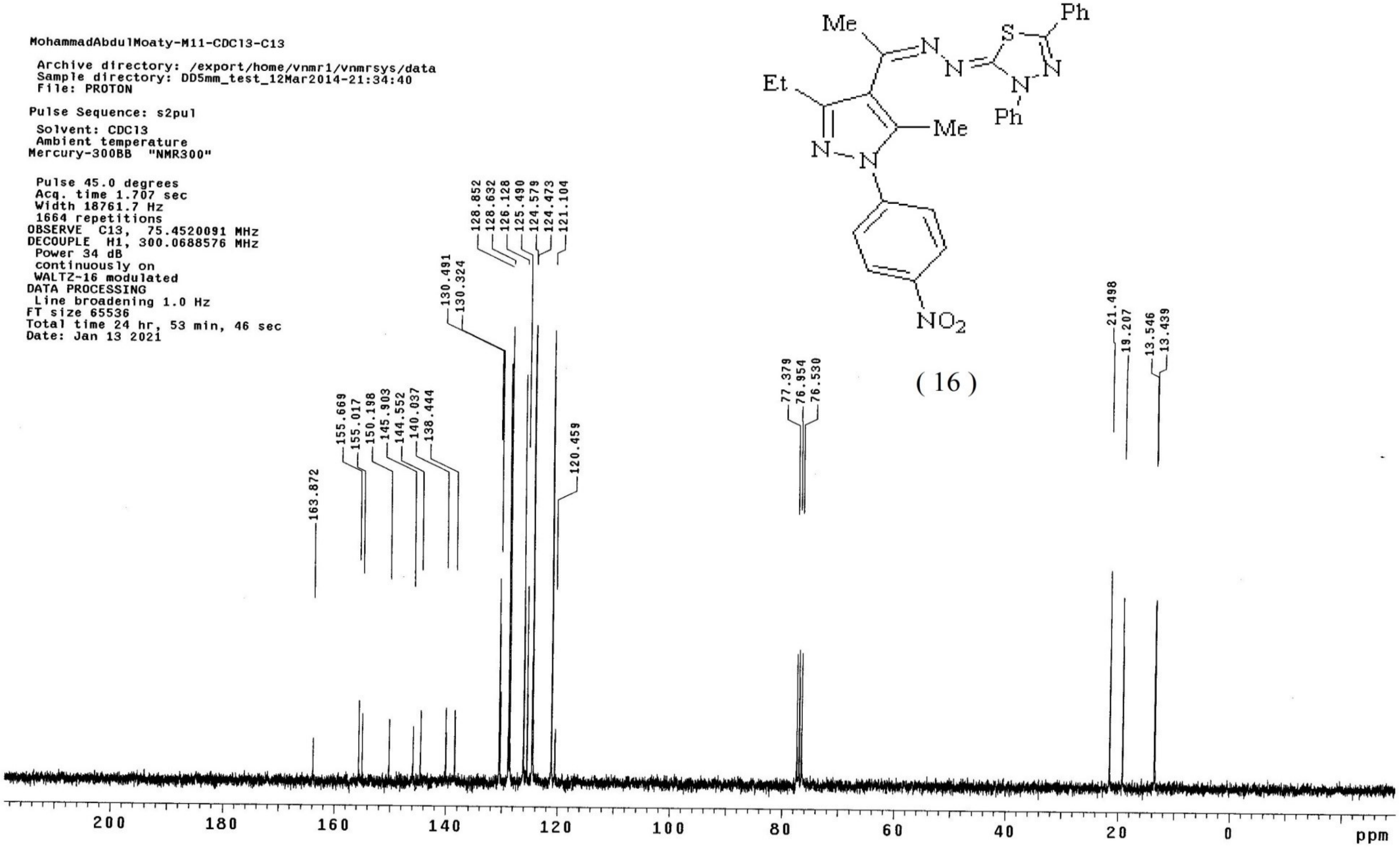
MohammadAbdulMoaty-M11-CDCl3-C13
Archive directory: /export/home/vnmr1/vnmrsys/data
Sample directory: DD5mm_test_12Mar2014-21:34:40
File: PROTON
Pulse Sequence: s2pul
Solvent: CDCl3
Ambient temperature
Mercury-300BB "NMR300"
Pulse 45.0 degrees
Acq. time 1.707 sec
Width 18761.7 Hz
1664 repetitions
OBSERVE C13, 75.4520091 MHz
DECOUPLE H1, 300.0688576 MHz
Power 34 dB
continuously on
WALTZ-16 modulated
DATA PROCESSING
Line broadening 1.0 Hz
FT size 65536
Total time 24 hr, 53 min, 46 sec
Date: Jan 13 2021
163.872
155.669
155.017
150.198
145.903
144.552
140.037
138.444
130.491
130.324
128.852
128.632
126.128
125.490
124.579
124.473
121.104
120.459
77.379
76.954
76.530
21.498
19.207
13.546
13.439
200
180
160
140
120
100
80
60
40
20
0
ppm
Me
Ph
N
N
S
N
N
Et
N
Ph
Me
N
N
NO_2
(16)

MernaTalat-M24-CDCl3-H1

Archive directory: /export/home/vnmr1/vnmrsys/data
Sample directory: DD5mm_test_12Mar2014-21:34:40
File: PROTON

Pulse Sequence: s2pul

Solvent: CDCl3
Temp. 30.0 C / 303.1 K
Mercury-300BB "NMR300"

Relax. delay 6.000 sec
Pulse 45.0 degrees
Acq. time 4.004 sec
Width 6600.7 Hz
8 repetitions
OBSERVE H1, 300.0673635 MHz
DATA PROCESSING
Line broadening 0.9 Hz
FT size 65536
Total time 58 min, 56 sec
Date: Mar 30 2021

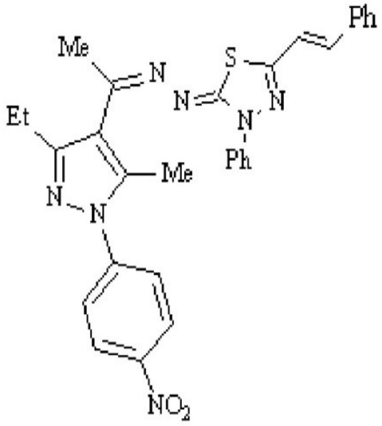

(17)

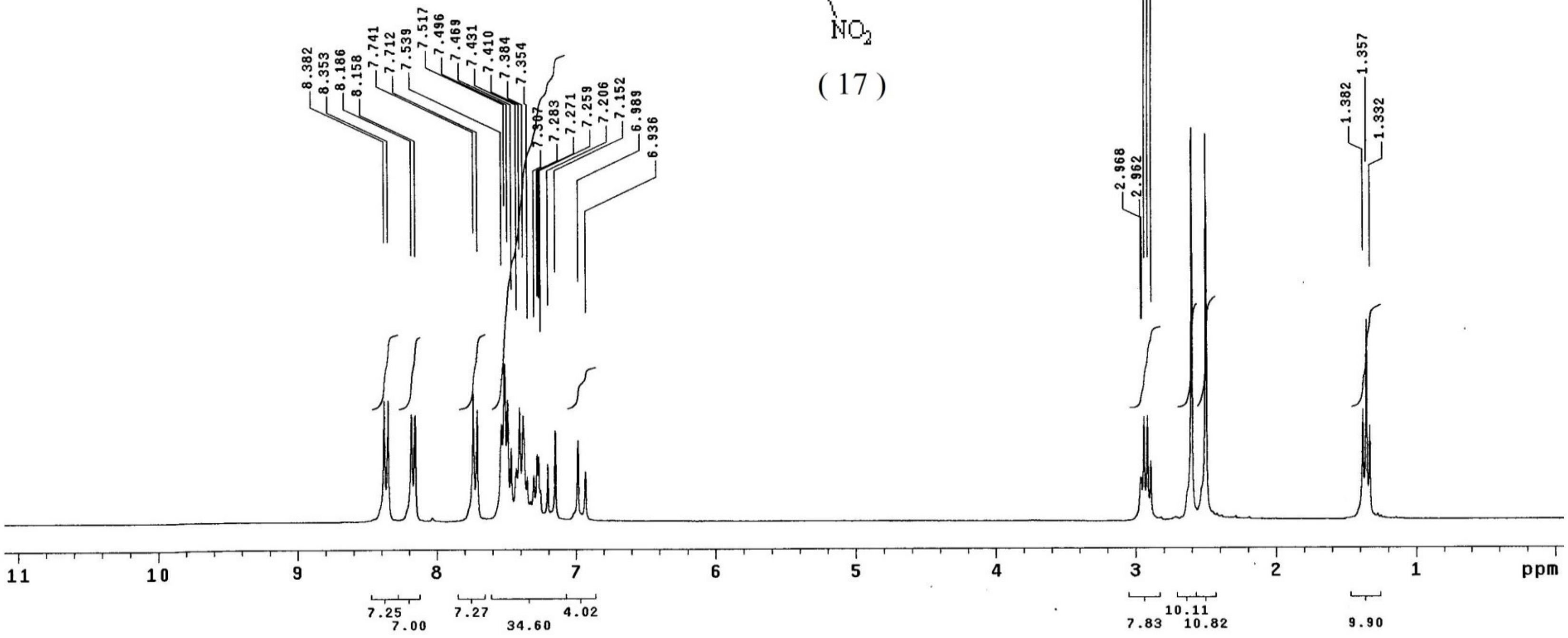

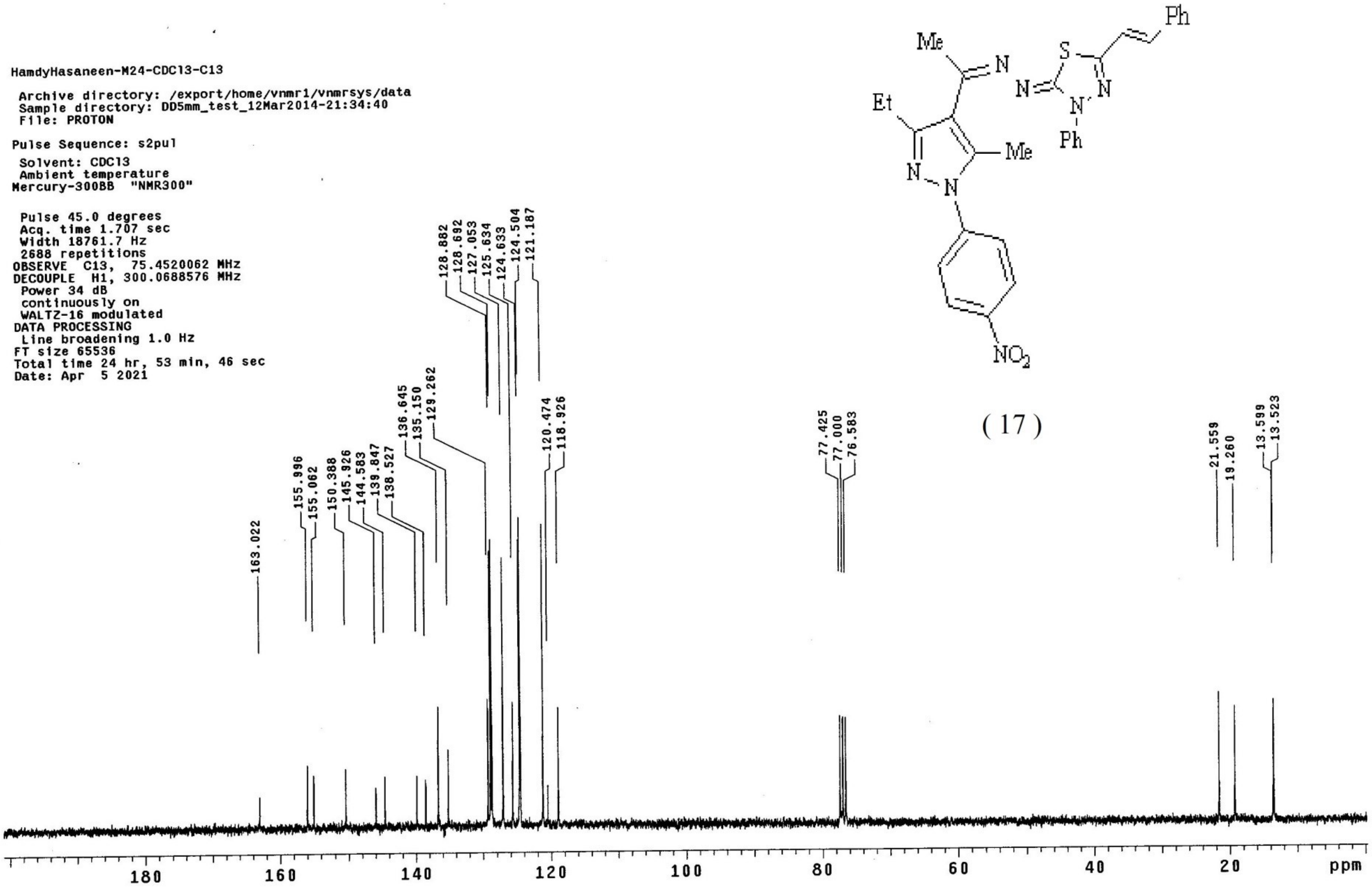

HamdyHasaneen-M24-CDCl3-C13
Archive directory: /export/home/vnmr1/vnmrsys/data
Sample directory: DD5mm_test_12Mar2014-21:34:40
File: PROTON
Pulse Sequence: s2pul
Solvent: CDCl3
Ambient temperature
Mercury-300BB "NMR300"
Pulse 45.0 degrees
Acq. time 1.707 sec
Width 18761.7 Hz
2688 repetitions
OBSERVE C13, 75.4520062 MHz
DECOUPLE H1, 300.0688576 MHz
Power 34 dB
continuously on
WALTZ-16 modulated
DATA PROCESSING
Line broadening 1.0 Hz
FT size 65536
Total time 24 hr, 53 min, 46 sec
Date: Apr 5 2021
163.022
155.996
155.062
150.388
145.926
144.583
139.847
138.527
136.645
135.150
129.262
128.882
128.692
127.053
125.634
124.633
124.504
121.187
120.474
118.926
77.425
77.000
76.583
21.559
19.260
13.599
13.523
Me
N
N
S
N
N
Ph
Et
Me
N
N
Ph
NO2
180
160
140
120
100
80
60
40
20
ppm

(17)

MernaTalat-M23-CDCl3-H1

Archive directory: /export/home/vnmr1/vnmrsys/data
Sample directory: DD5mm_test_12Mar2014-21:34:40
File: PROTON

Pulse Sequence: s2pul

Solvent: CDCl3
Temp. 30.0 C / 303.1 K
Mercury-300BB "NMR300"

Relax. delay 6.000 sec
Pulse 45.0 degrees
Acq. time 4.004 sec
Width 6600.7 Hz
9 repetitions
OBSERVE H1, 300.0673635 MHz
DATA PROCESSING
Line broadening 0.9 Hz
FT size 65536
Total time 58 min, 56 sec
Date: Mar 30 2021

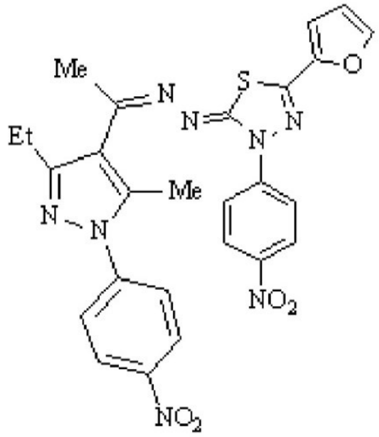

(18)

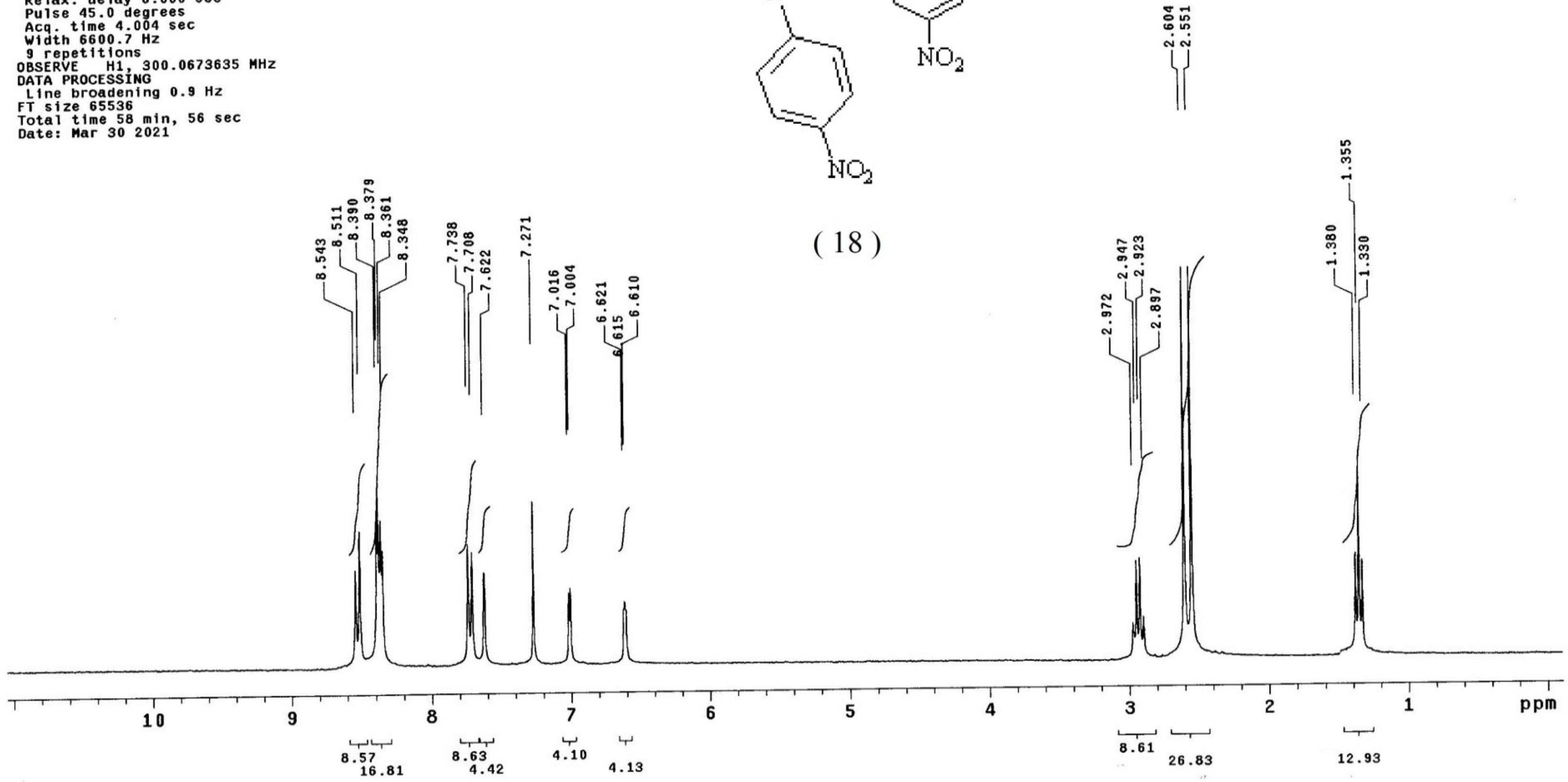

MernaTalat-M23-CDCl3-C13

Archive directory: /export/home/vnmr1/vnmrsys/data
Sample directory: DD5mm_test_12Mar2014-21:34:40
File: PROTON

Pulse Sequence: s2pul

Solvent: CDCl3
Ambient temperature
Mercury-300BB "NMR300"

Pulse 45.0 degrees
Acq. time 1.707 sec
Width 18761.7 Hz
2968 repetitions
OBSERVE C13, 75.4520074 MHz
DECOUPLE H1, 300.0688576 MHz
Power 34 dB
continuously on
WALTZ-16 modulated
DATA PROCESSING
Line broadening 1.0 Hz
FT size 65536
Total time 24 hr, 53 min, 46 sec
Date: Apr 13 2021

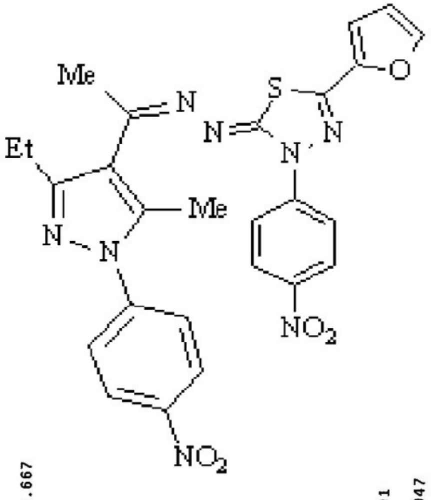

(18)

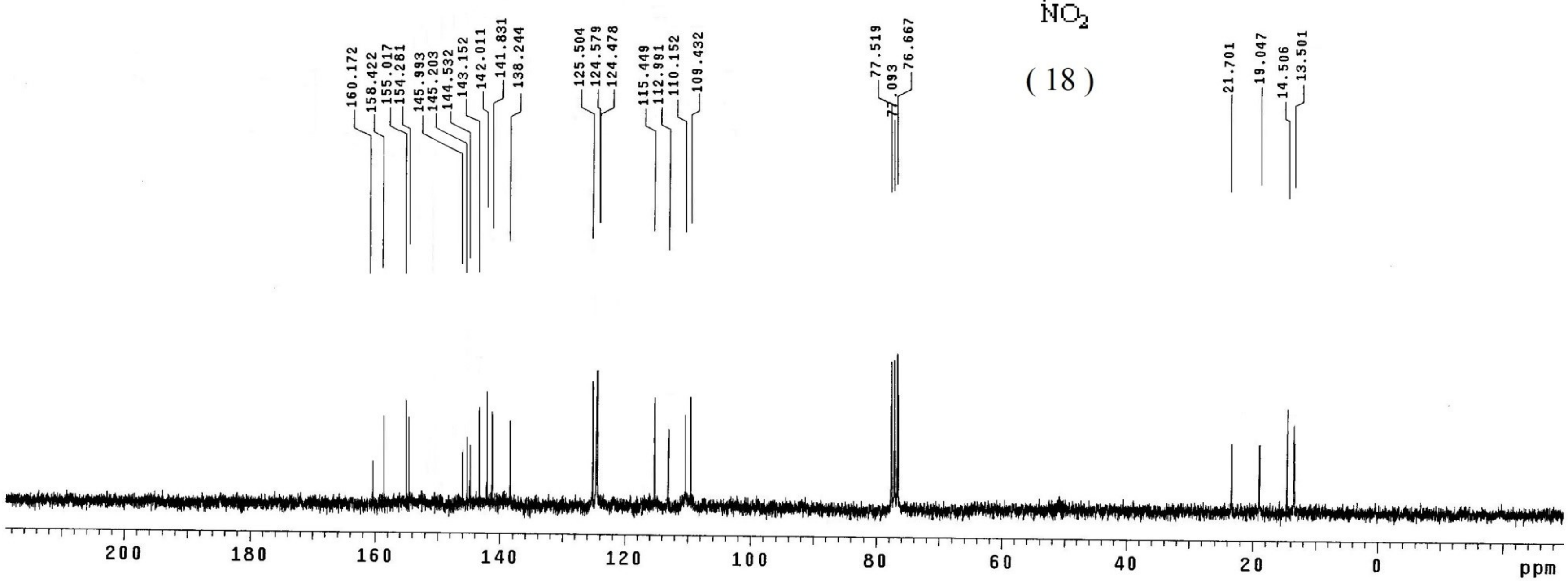

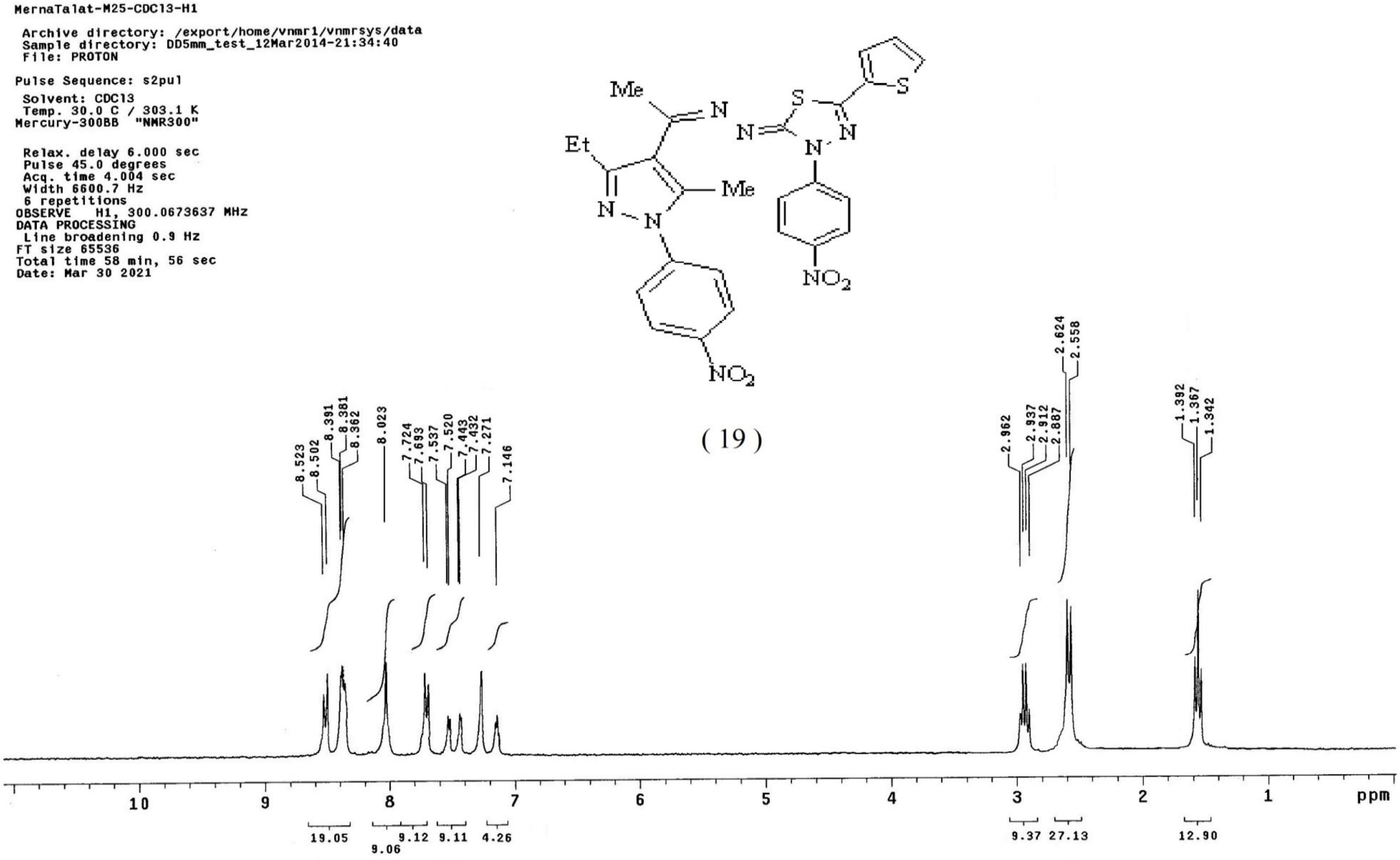
MernaTalat-M25-CDCl3-H1
Archive directory: /export/home/vnmr1/vnmrsys/data
Sample directory: DD5mm_test_12Mar2014-21:34:40
File: PROTON
Pulse Sequence: s2pul
Solvent: CDCl3
Temp. 30.0 C / 303.1 K
Mercury-300BB "NMR300"
Relax. delay 6.000 sec
Pulse 45.0 degrees
Acq. time 4.004 sec
Width 6600.7 Hz
6 repetitions
OBSERVE H1, 300.0673637 MHz
DATA PROCESSING
Line broadening 0.9 Hz
FT size 65536
Total time 58 min, 56 sec
Date: Mar 30 2021
Me
N
Et
Me
N
N
S
N
N
N
S
N
NO2
NO2
(19)
8.523
8.502
8.391
8.381
8.362
8.023
7.724
7.693
7.537
7.520
7.443
7.432
7.271
7.146
2.962
2.937
2.912
2.887
2.624
2.558
1.392
1.367
1.342
10
9
8
7
6
5
4
3
2
1
ppm
19.05
9.06
9.12
9.11
4.26
9.37
27.13
12.90

MernaTalaat-M25-CDCl3-C13

Archive directory: /export/home/vnmr1/vnmrsys/data
Sample directory: DD5mm_test_12Mar2014-21:34:40
File: PROTON

Pulse Sequence: s2pul

Solvent: CDCl3
Ambient temperature
Mercury-300BB "NMR300"

Pulse 45.0 degrees
Acq. time 1.707 sec
Width 18761.7 Hz
3064 repetitions
OBSERVE C13, 75.4520034 MHz
DECOUPLE H1, 300.0688576 MHz
Power 34 dB
continuously on
WALTZ-16 modulated
DATA PROCESSING
Line broadening 1.0 Hz
FT size 65536
Total time 31 hr, 7 min, 12 sec
Date: Aug 30 2021

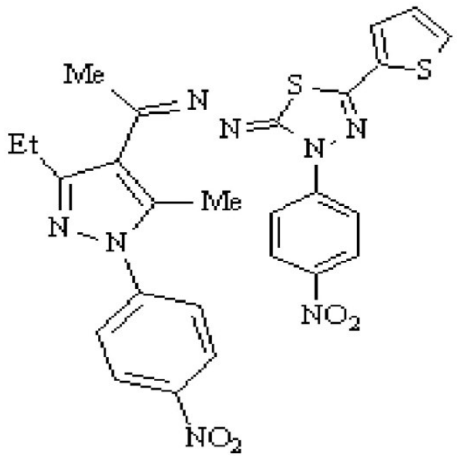

(19)

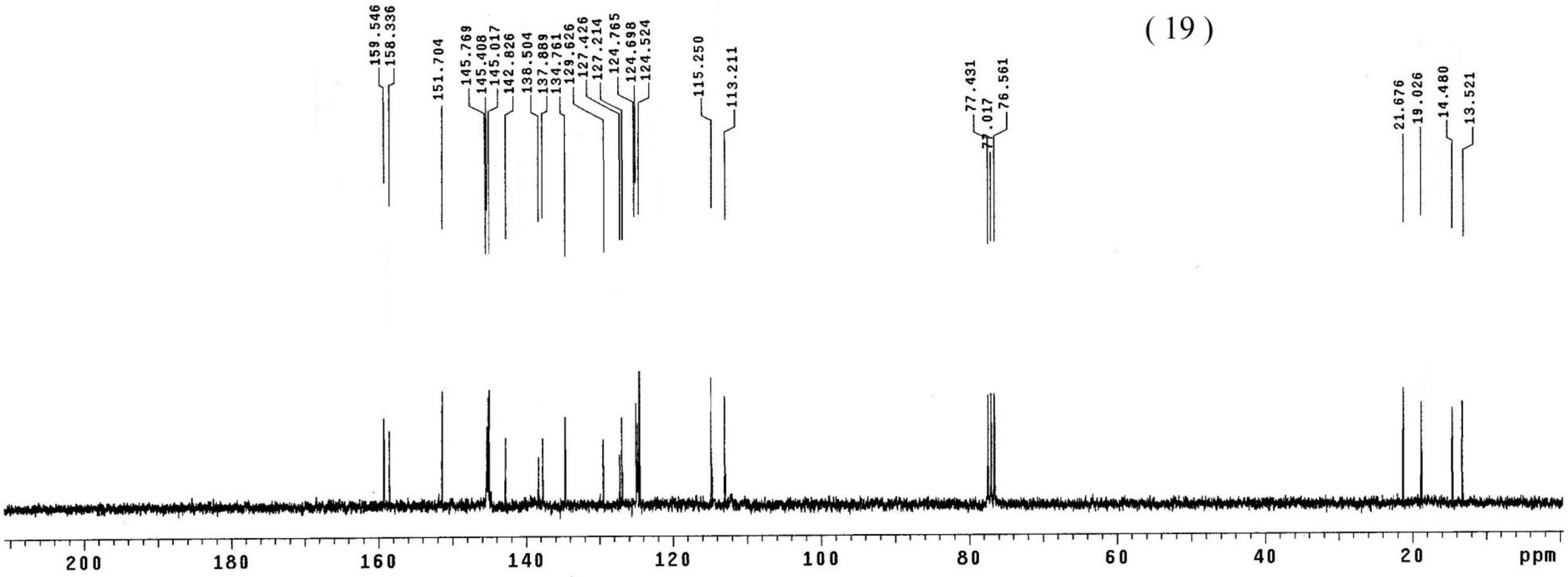

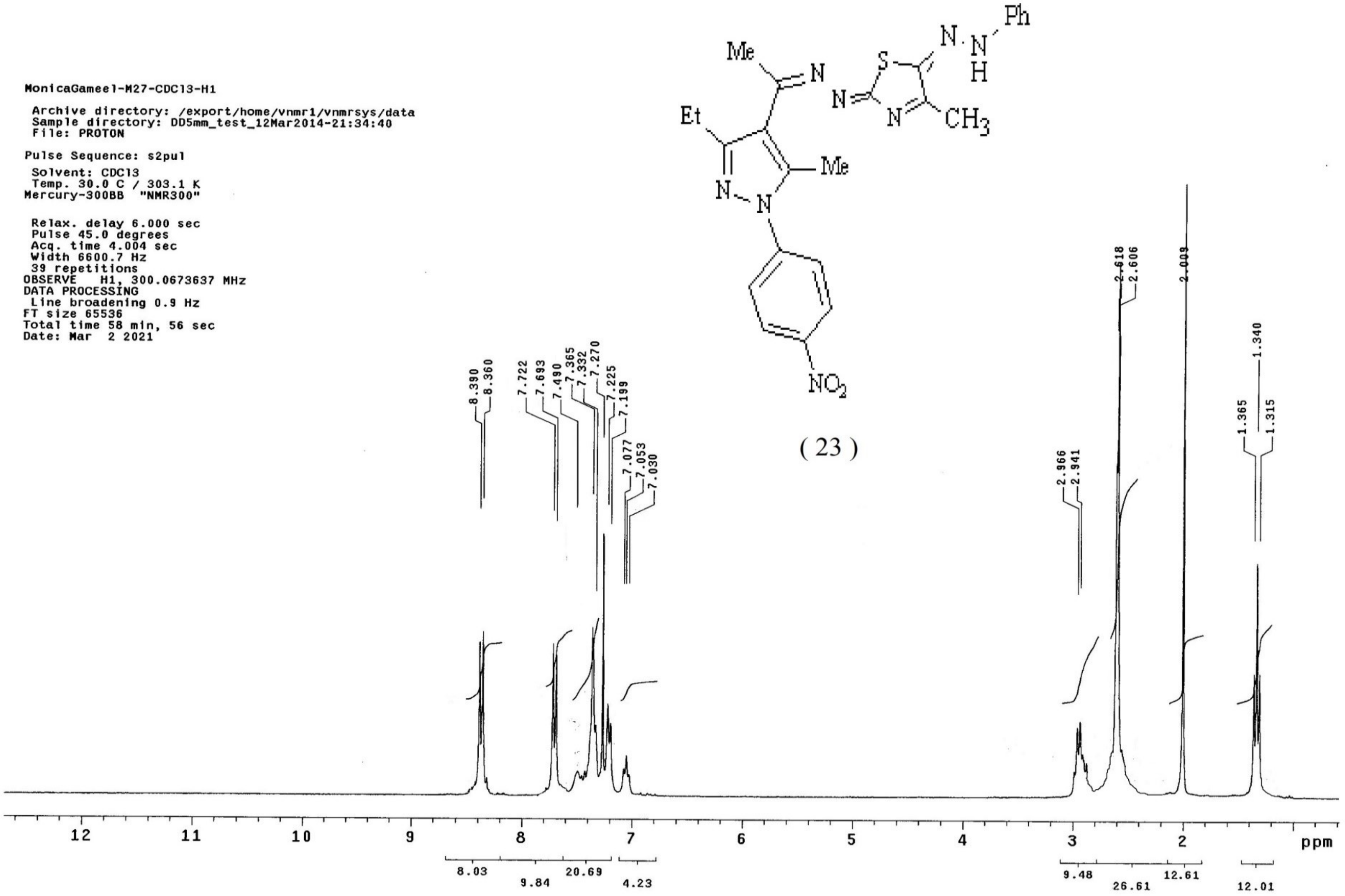
MonicaGameel-M27-CDCl3-H1
Archive directory: /export/home/vnmr1/vnmrsys/data
Sample directory: DD5mm_test_12Mar2014-21:34:40
File: PROTON
Pulse Sequence: s2pul
Solvent: CDCl3
Temp. 30.0 C / 303.1 K
Mercury-300BB "NMR300"
Relax. delay 6.000 sec
Pulse 45.0 degrees
Acq. time 4.004 sec
Width 6600.7 Hz
39 repetitions
OBSERVE H1, 300.0673637 MHz
DATA PROCESSING
Line broadening 0.9 Hz
FT size 65536
Total time 58 min, 56 sec
Date: Mar 2 2021
Me
N
N
S
N
N
H
Ph
Et
CH3
Me
N
N
NO2
8.390
8.360
7.722
7.693
7.490
7.365
7.332
7.270
7.225
7.199
7.077
7.053
7.030
2.966
2.941
2.618
2.606
2.009
1.365
1.340
1.315
12
11
10
9
8
7
6
5
4
3
2
ppm
8.03
9.84
20.69
4.23
9.48
26.61
12.61
12.01

(23)

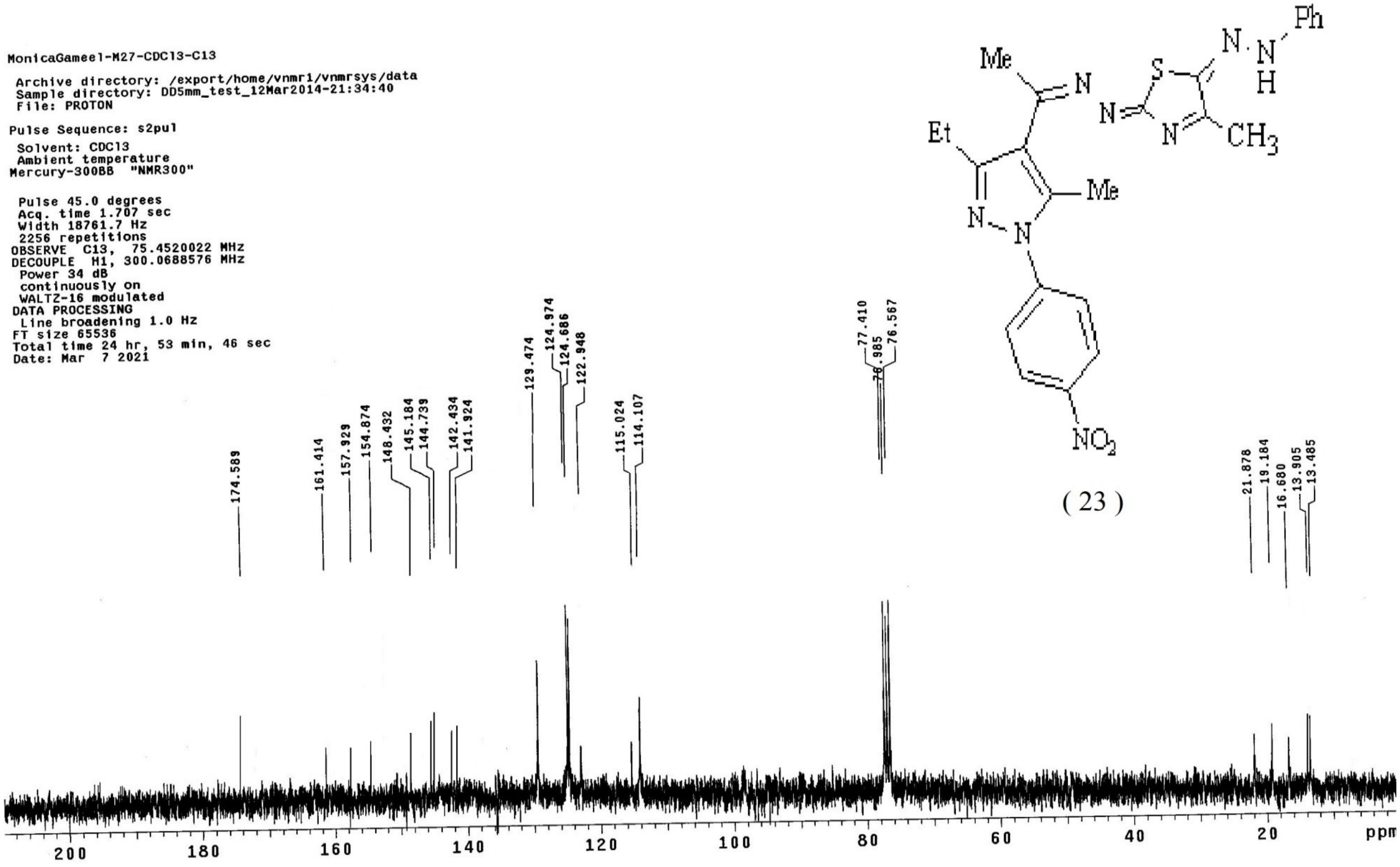
MonicaGameel-M27-CDCl3-C13
Archive directory: /export/home/vnmr1/vnmrsys/data
Sample directory: DD5mm_test_12Mar2014-21:34:40
File: PROTON
Pulse Sequence: s2pul
Solvent: CDCl3
Ambient temperature
Mercury-300BB "NMR300"
Pulse 45.0 degrees
Acq. time 1.707 sec
Width 18761.7 Hz
2256 repetitions
OBSERVE C13, 75.4520022 MHz
DECOUPLE H1, 300.0688576 MHz
Power 34 dB
continuously on
WALTZ-16 modulated
DATA PROCESSING
Line broadening 1.0 Hz
FT size 65536
Total time 24 hr, 53 min, 46 sec
Date: Mar 7 2021
174.589
161.414
157.929
154.874
148.432
145.184
144.739
142.434
141.924
129.474
124.974
124.686
122.948
115.024
114.107
77.410
76.985
76.567
21.878
19.184
16.680
13.905
13.485
Me
N
S
N
N
Ph
H
Et
N
CH3
Me
N
N
NO2
200
180
160
140
120
100
80
60
40
20
ppm

(23)

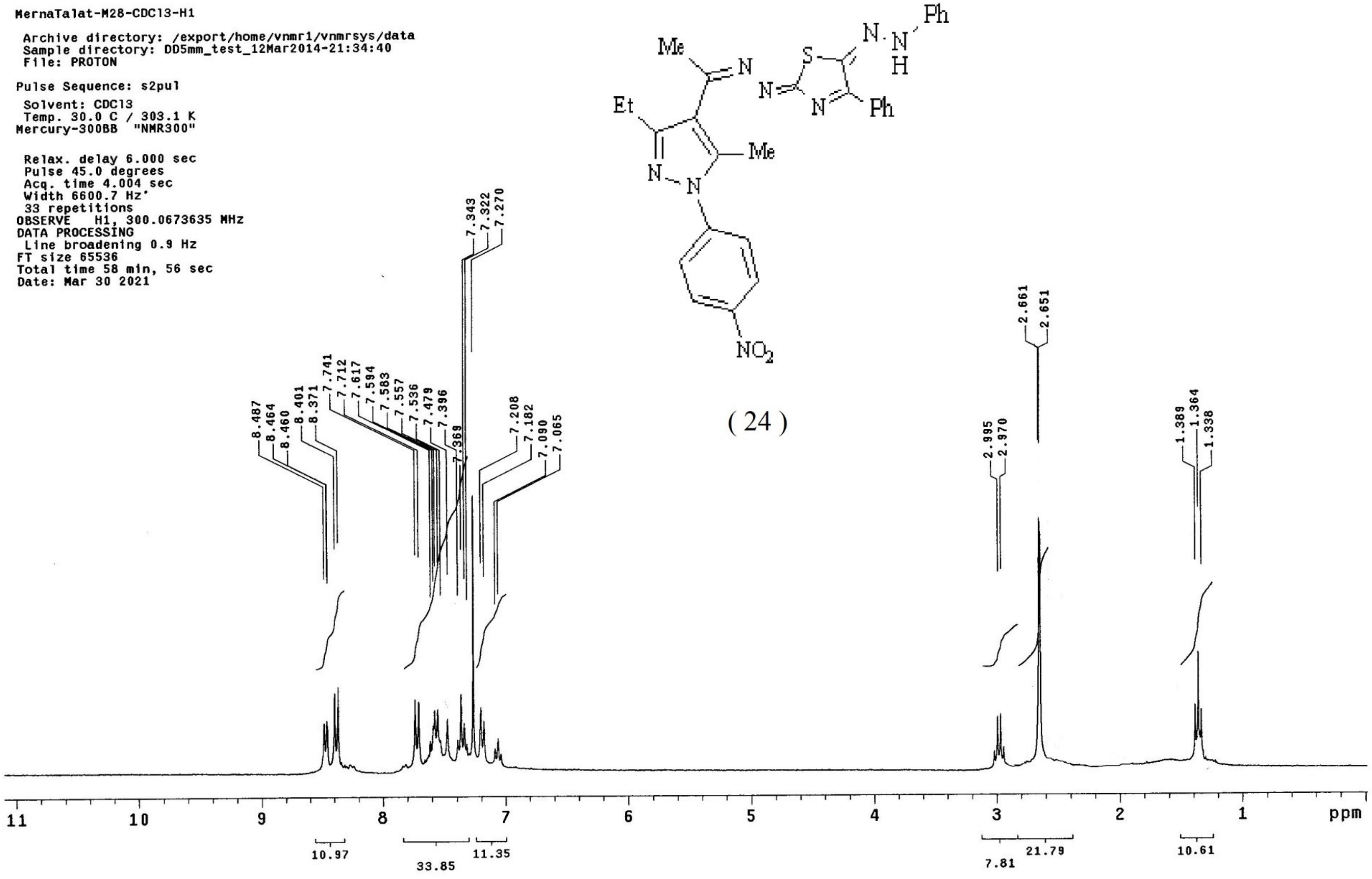
MernaTalat-M28-CDCl3-H1
Archive directory: /export/home/vnmr1/vnmrsys/data
Sample directory: DD5mm_test_12Mar2014-21:34:40
File: PROTON
Pulse Sequence: s2pul
Solvent: CDCl3
Temp. 30.0 C / 303.1 K
Mercury-300BB "NMR300"
Relax. delay 6.000 sec
Pulse 45.0 degrees
Acq. time 4.004 sec
Width 6600.7 Hz
33 repetitions
OBSERVE H1, 300.0673635 MHz
DATA PROCESSING
Line broadening 0.9 Hz
FT size 65536
Total time 58 min, 56 sec
Date: Mar 30 2021
Me
N
S
N
N
H
Ph
Et
N
Ph
Me
N
N
NO2
(24)
8.487
8.464
8.460
8.401
8.371
7.741
7.712
7.617
7.594
7.583
7.557
7.536
7.479
7.396
7.369
7.343
7.322
7.270
7.208
7.182
7.090
7.065
2.995
2.970
2.661
2.651
1.389
1.364
1.338
11
10
9
8
7
6
5
4
3
2
1
ppm
10.97
33.85
11.35
7.81
21.79
10.61

HamdyHasaneen-M28-CDCl3-C13

Archive directory: /export/home/vnmr1/vnmrsys/data
Sample directory: DD5mm_test_12Mar2014-21:34:40
File: PROTON

Pulse Sequence: s2pul

Solvent: CDCl3
Ambient temperature
Mercury-300BB "NMR300"

Pulse 45.0 degrees
Acq. time 1.707 sec
Width 18761.7 Hz
1488 repetitions
OBSERVE C13, 75.4520165 MHz
DECOUPLE H1, 300.0688576 MHz
Power 34 dB
continuously on
WALTZ-16 modulated
DATA PROCESSING
Line broadening 1.0 Hz
FT size 65536
Total time 31 hr, 7 min, 12 sec
Date: Mar 31 2021

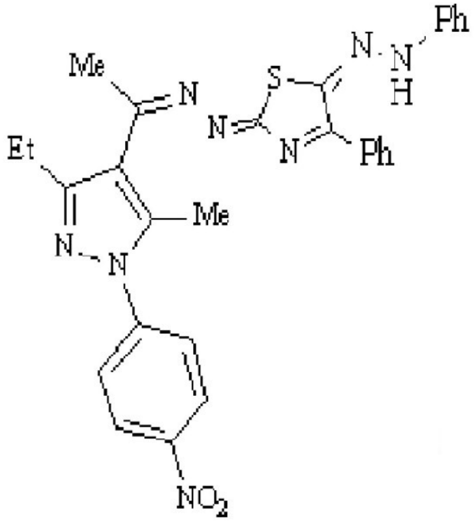

(24)

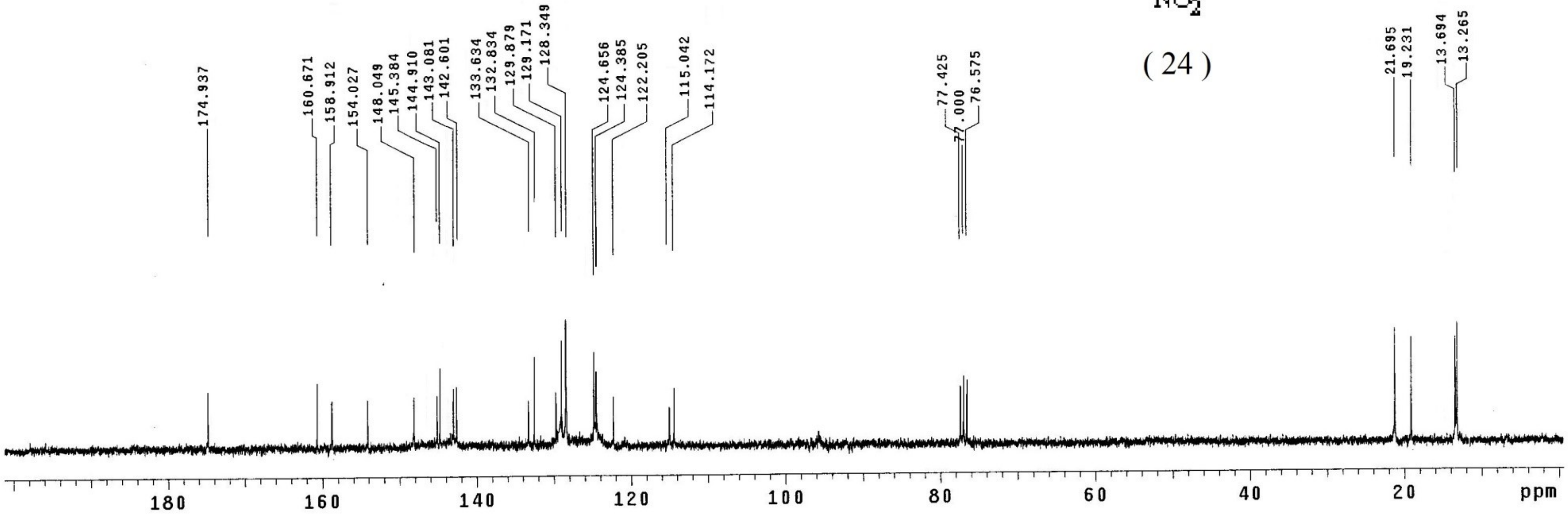

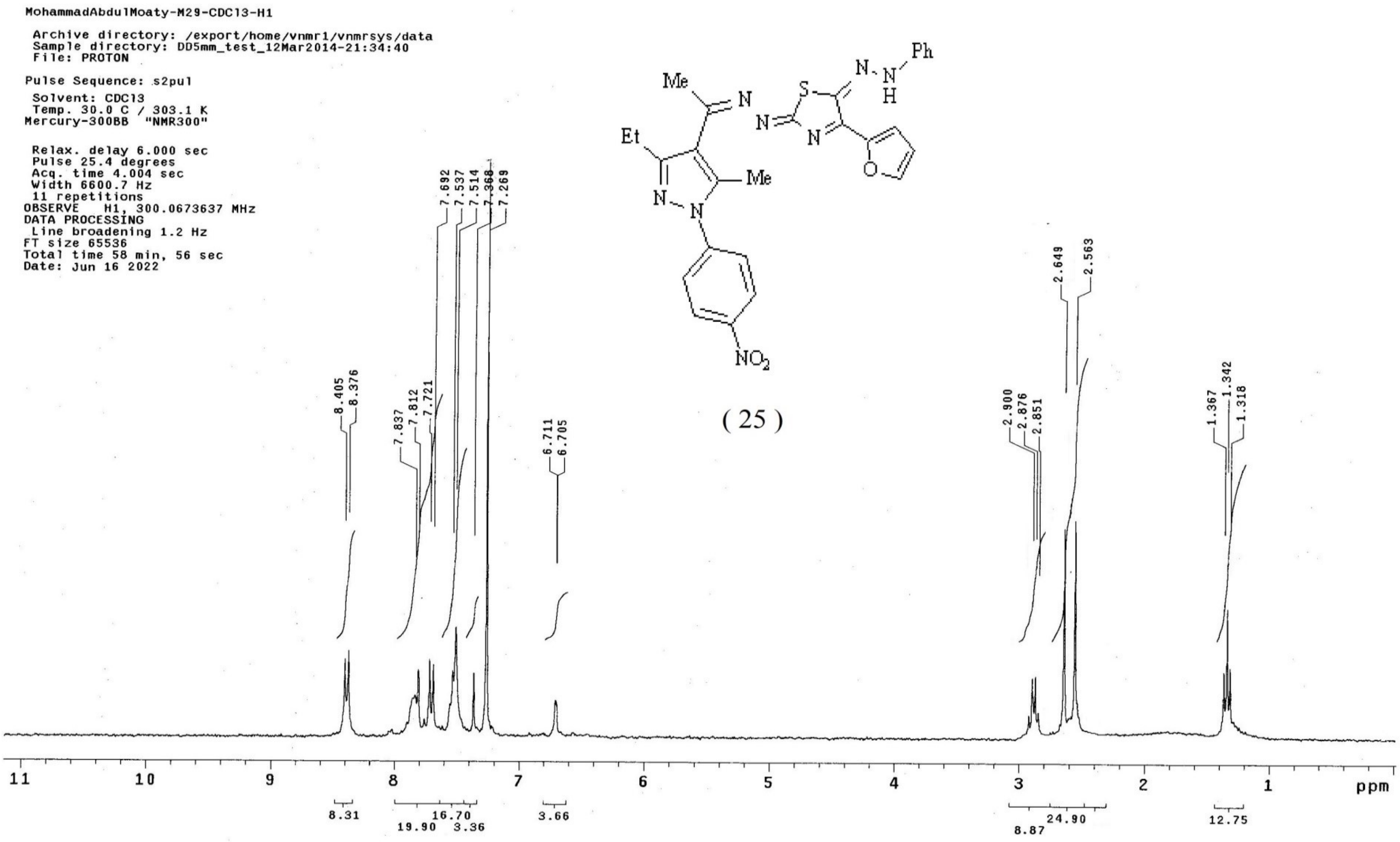
MohammadAbdulMoaty-M29-CDCl3-H1
Archive directory: /export/home/vnmr1/vnmrsys/data
Sample directory: DD5mm_test_12Mar2014-21:34:40
File: PROTON
Pulse Sequence: s2pul
Solvent: CDCl3
Temp. 30.0 C / 303.1 K
Mercury-300BB "NMR300"
Relax. delay 6.000 sec
Pulse 25.4 degrees
Acq. time 4.004 sec
Width 6600.7 Hz
11 repetitions
OBSERVE H1, 300.0673637 MHz
DATA PROCESSING
Line broadening 1.2 Hz
FT size 65536
Total time 58 min, 56 sec
Date: Jun 16 2022
ppm

(25)

HamdyHasaneen-M29-CDCl3-C13

Archive directory: /export/home/vnmr1/vnmrsys/data
Sample directory: DD5mm_test_12Mar2014-21:34:40
File: PROTON

Pulse Sequence: s2pul

Solvent: CDCl3
Ambient temperature
Mercury-300BB "NMR300"

Pulse 45.0 degrees
Acq. time 1.707 sec
Width 18761.7 Hz
2472 repetitions
OBSERVE C13, 75.4520034 MHz
DECOUPLE H1, 300.0688576 MHz
Power 34 dB
continuously on
WALTZ-16 modulated
DATA PROCESSING
Line broadening 1.0 Hz
FT size 65536
Total time 31 hr, 7 min, 12 sec
Date: Oct 6 2021

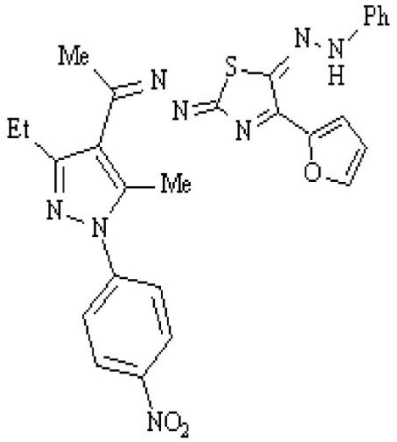

(25)

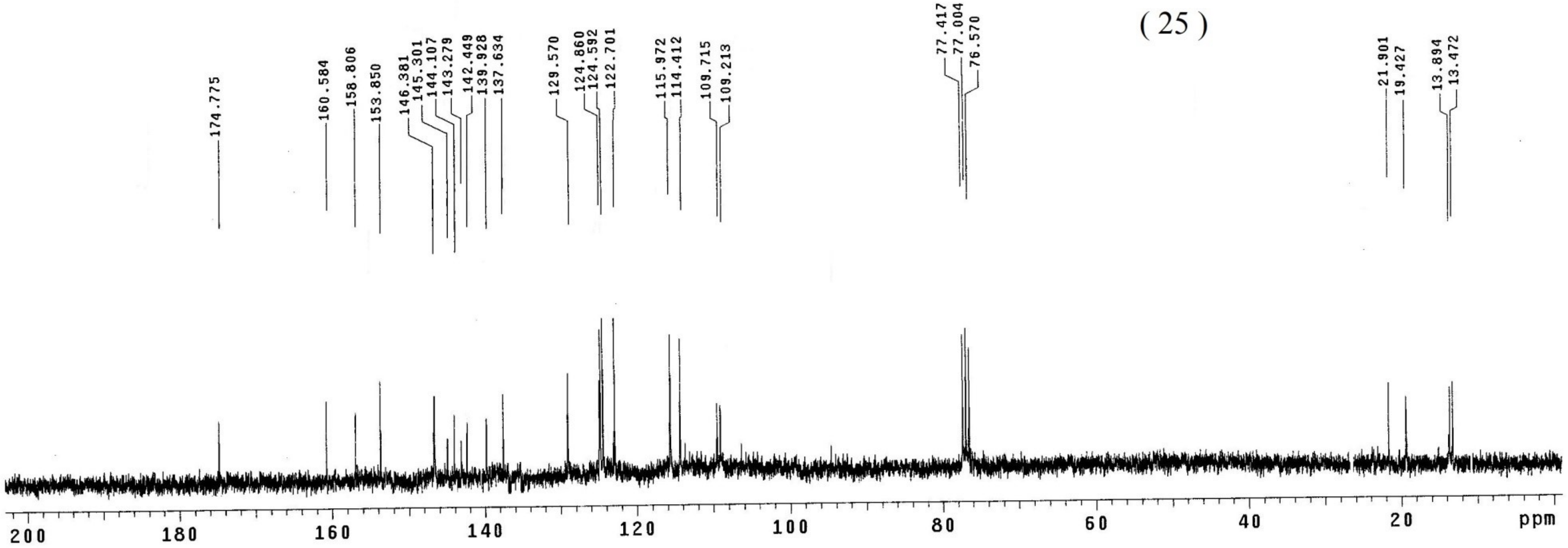

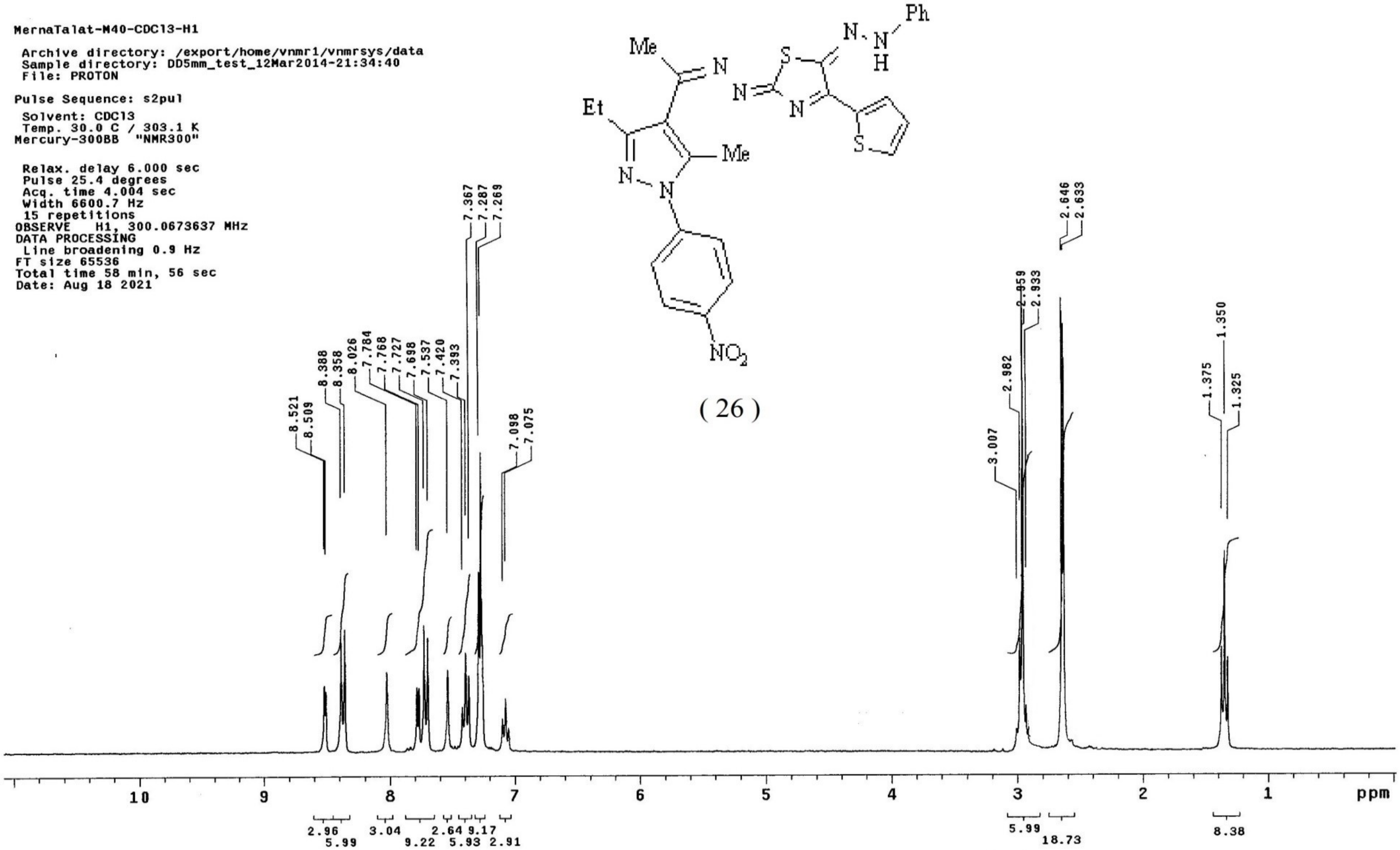
MernaTalat-M40-CDCl3-H1
Archive directory: /export/home/vnmr1/vnmrsys/data
Sample directory: DD5mm_test_12Mar2014-21:34:40
File: PROTON
Pulse Sequence: s2pul
Solvent: CDCl3
Temp. 30.0 C / 303.1 K
Mercury-300BB "NMR300"
Relax. delay 6.000 sec
Pulse 25.4 degrees
Acq. time 4.004 sec
Width 6600.7 Hz
15 repetitions
OBSERVE H1, 300.0673637 MHz
DATA PROCESSING
Line broadening 0.9 Hz
FT size 65536
Total time 58 min, 56 sec
Date: Aug 18 2021
Me
N
N
S
N
N
H
Ph
Et
Me
N
N
S
NO2
(26)
8.521
8.509
8.388
8.358
8.026
7.784
7.768
7.727
7.698
7.537
7.420
7.393
7.367
7.287
7.269
7.098
7.075
3.007
2.982
2.959
2.933
2.646
2.633
1.375
1.350
1.325
10
9
8
7
6
5
4
3
2
1
ppm
2.96
5.99
3.04
9.22
2.64
5.93
9.17
2.91
5.99
18.73
8.38

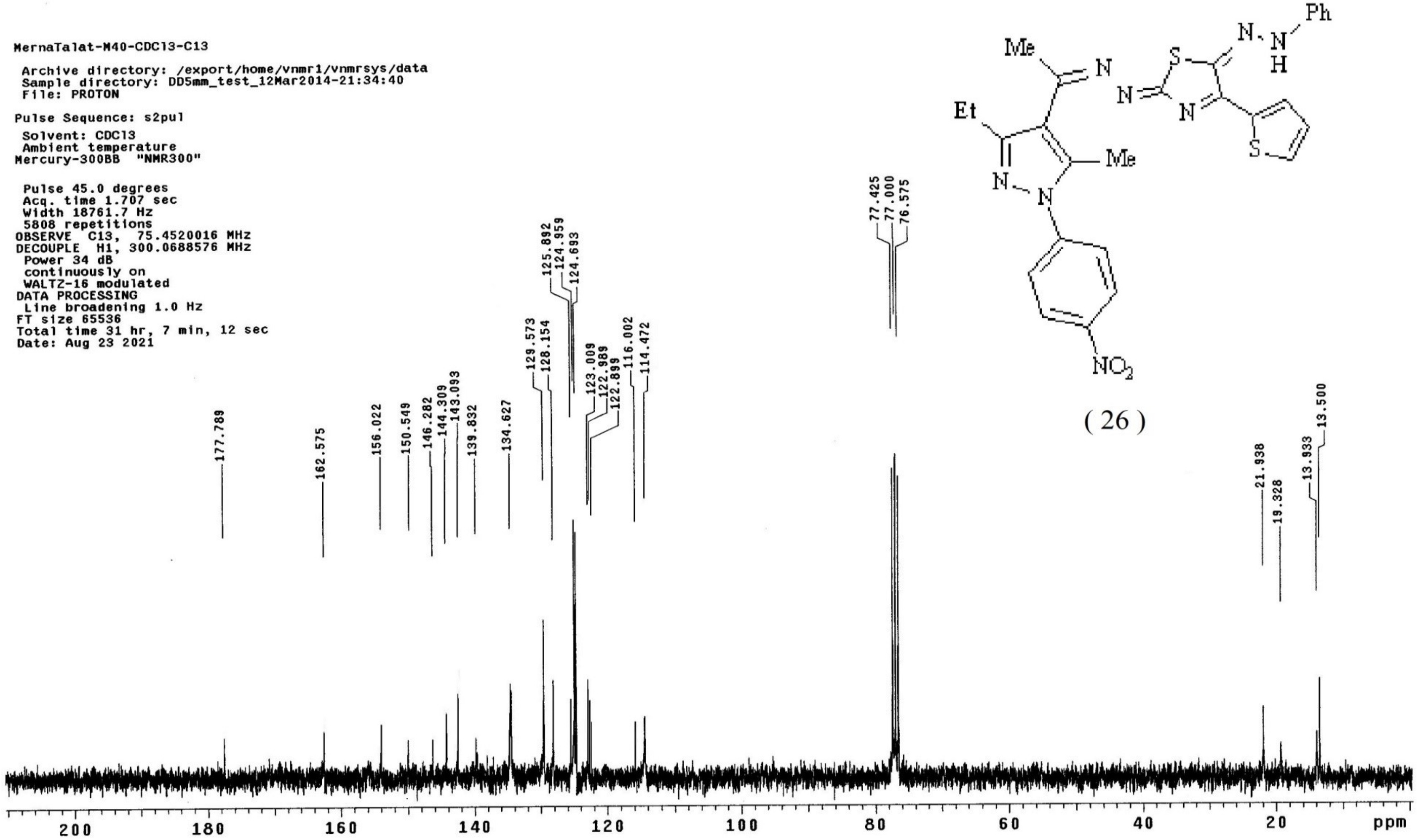
MernaTalat-M40-CDCl3-C13
Archive directory: /export/home/vnmr1/vnmrsys/data
Sample directory: DD5mm_test_12Mar2014-21:34:40
File: PROTON
Pulse Sequence: s2pul
Solvent: CDCl3
Ambient temperature
Mercury-300BB "NMR300"
Pulse 45.0 degrees
Acq. time 1.707 sec
Width 18761.7 Hz
5808 repetitions
OBSERVE C13, 75.4520016 MHz
DECOUPLE H1, 300.0688576 MHz
Power 34 dB
continuously on
WALTZ-16 modulated
DATA PROCESSING
Line broadening 1.0 Hz
FT size 65536
Total time 31 hr, 7 min, 12 sec
Date: Aug 23 2021
177.789
162.575
156.022
150.549
146.282
144.309
143.093
139.832
134.627
129.573
128.154
125.892
124.959
124.693
123.009
122.989
122.899
116.002
114.472
77.425
77.000
76.575
21.938
19.328
13.933
13.500
200
180
160
140
120
100
80
60
40
20
ppm
Me
N
N
S
N
N
N
H
Ph
Et
Me
N
N
S
NO2
(26)

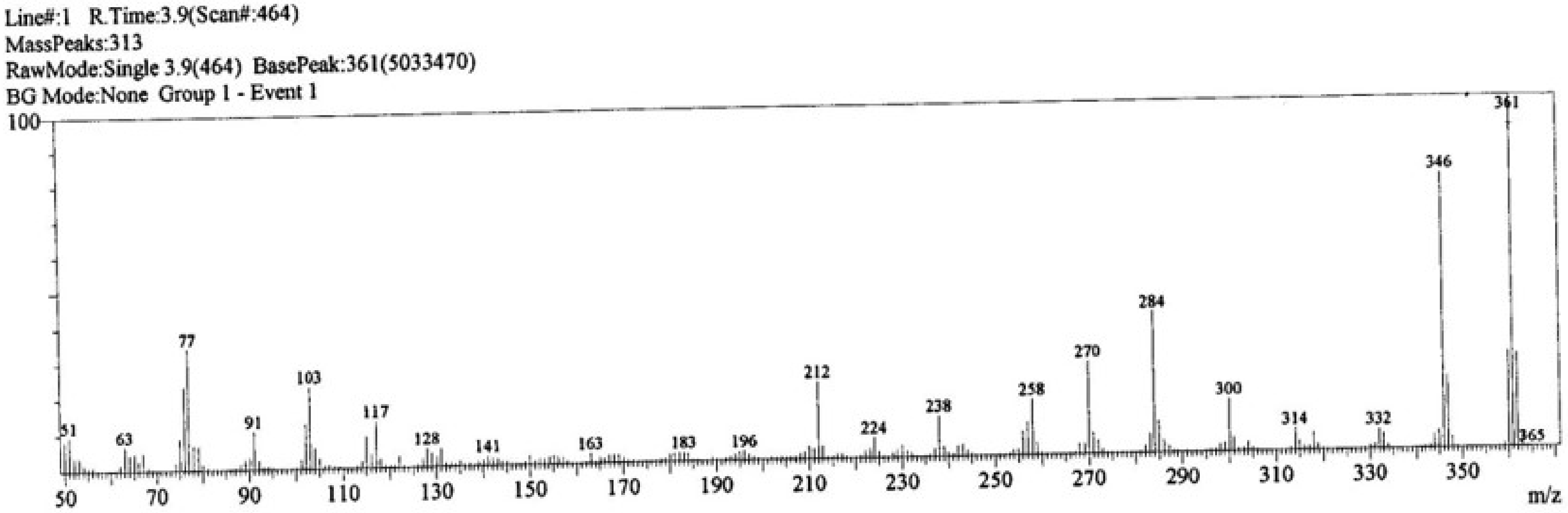

Mass Table
Line#:1 R.Time:3.9(Scan#:464)
MassPeaks:313
RawMode:Single 3.9(464) BasePeak:361(5033470)
BG Mode:None Group 1 - Event 1

#	m/z	Abs. In	Rel. Int.
1	50.05	403995	8.03
2	51.00	482354	9.58
3	52.05	189657	3.77
4	53.05	180497	3.59
5	54.05	82369	1.64
6	55.05	52016	1.03
7	56.05	55906	1.11
8	57.10	18018	0.36
9	58.05	2991	0.06

Mass spectrometry of compound **4**.

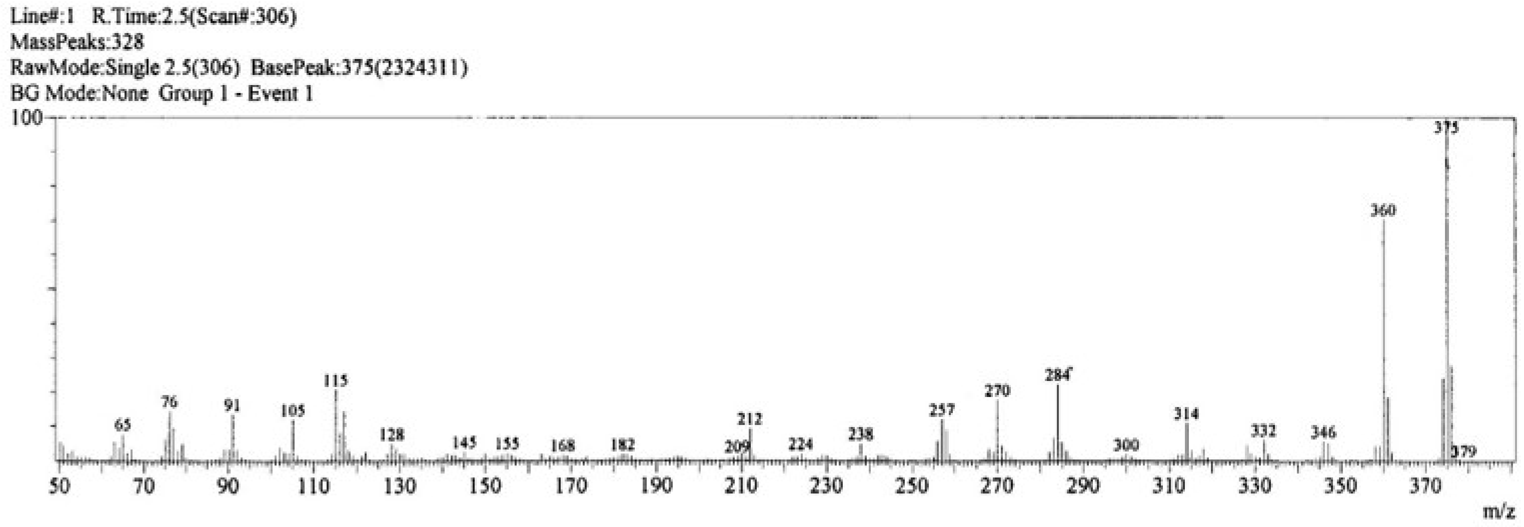

Mass Table
Line#:1 R.Time:2.5(Scan#:306)
MassPeaks:328
RawMode:Single 2.5(306) BasePeak:375(2324311)
BG Mode:None Group 1 - Event 1

#	m/z	Abs. In	Rel. Int.	#	m/z	Abs. In	Rel. Int.	#	m/z	Abs. In	Rel. Int.
1	50.05	130298	5.61	4	53.05	68307	2.94	7	56.10	26516	1.14
2	51.05	102015	4.39	5	54.10	32065	1.38	8	57.10	19106	0.82
3	52.05	55471	2.39	6	55.05	29480	1.27	9	58.00	4439	0.19

Mass spectrometry of compound **7**.

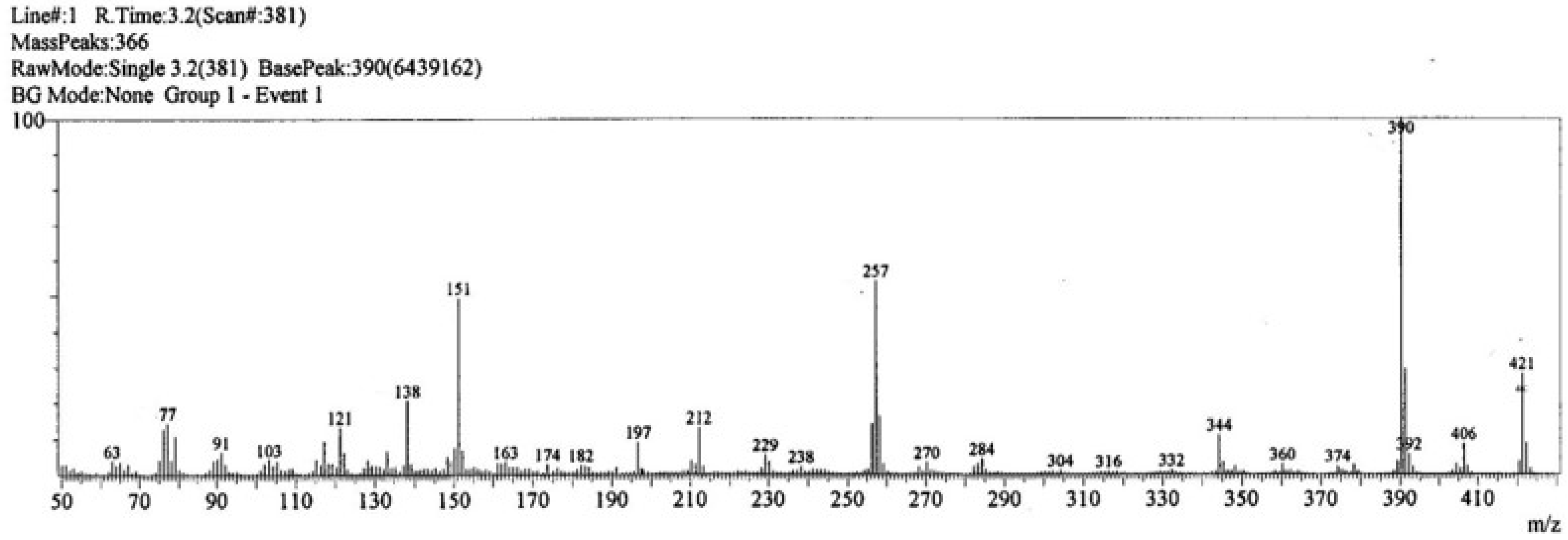

Mass Table
Line#:1 R.Time:3.2(Scan#:381)
MassPeaks:366
RawMode:Single 3.2(381) BasePeak:390(6439162)
BG Mode:None Group 1 - Event 1

#	m/z	Abs. In	Rel. Int.	#	m/z	Abs. In	Rel. Int.	#	m/z	Abs. In	Rel. Int.
1	50.05	171915	2.67	4	53.10	113916	1.77	7	56.10	39190	0.61
2	51.05	179539	2.79	5	54.10	52082	0.81	8	57.10	25751	0.40
3	52.10	83591	1.30	6	55.05	77956	1.21	9	58.15	4574	0.07

Mass spectrometry of compound **9**.

Line#:1 R.Time:2.1(Scan#:255)
MassPeaks:279
RawMode:Single 2.1(255) BasePeak:225(289166)
BG Mode:None Group 1 - Event 1

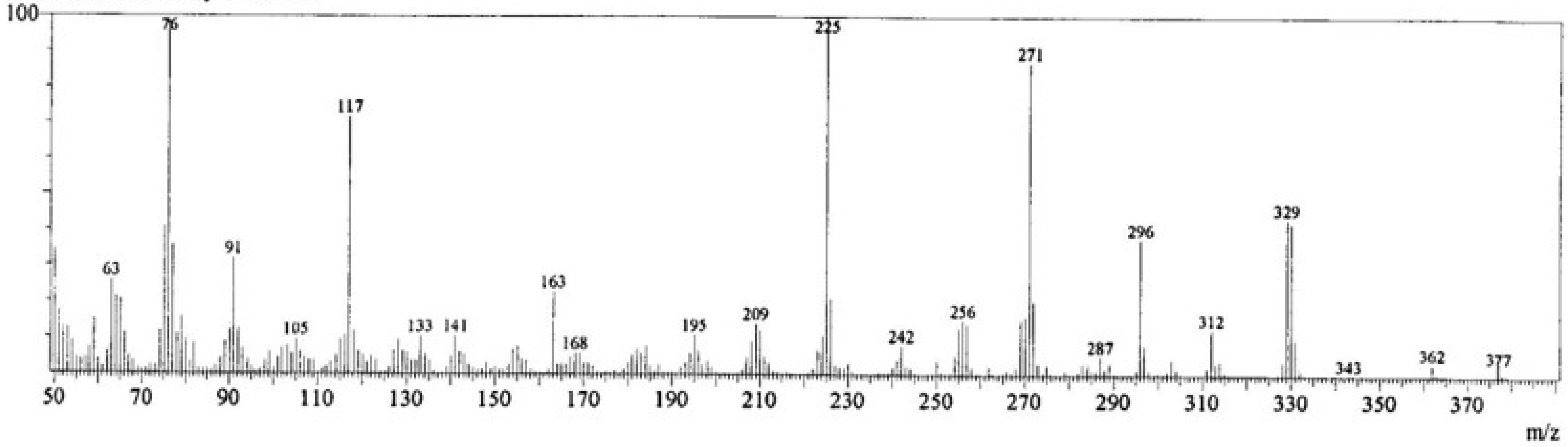

Mass Table
Line#:1 R.Time:2.1(Scan#:255)
MassPeaks:279
RawMode:Single 2.1(255) BasePeak:225(289166)
BG Mode:None Group 1 - Event 1

#	m/z	Abs. In	Rel. Int.	#	m/z	Abs. In	Rel. Int.	#	m/z	Abs. In	Rel. Int.
1	50.00	100018	34.59	4	53.05	36340	12.57	7	56.10	11215	3.88
2	51.05	49651	17.17	5	54.05	26017	9.00	8	57.05	12186	4.21
3	52.05	37384	12.93	6	55.10	13516	4.67	9	58.00	20849	7.21

Mass spectrometry of compound **13**.

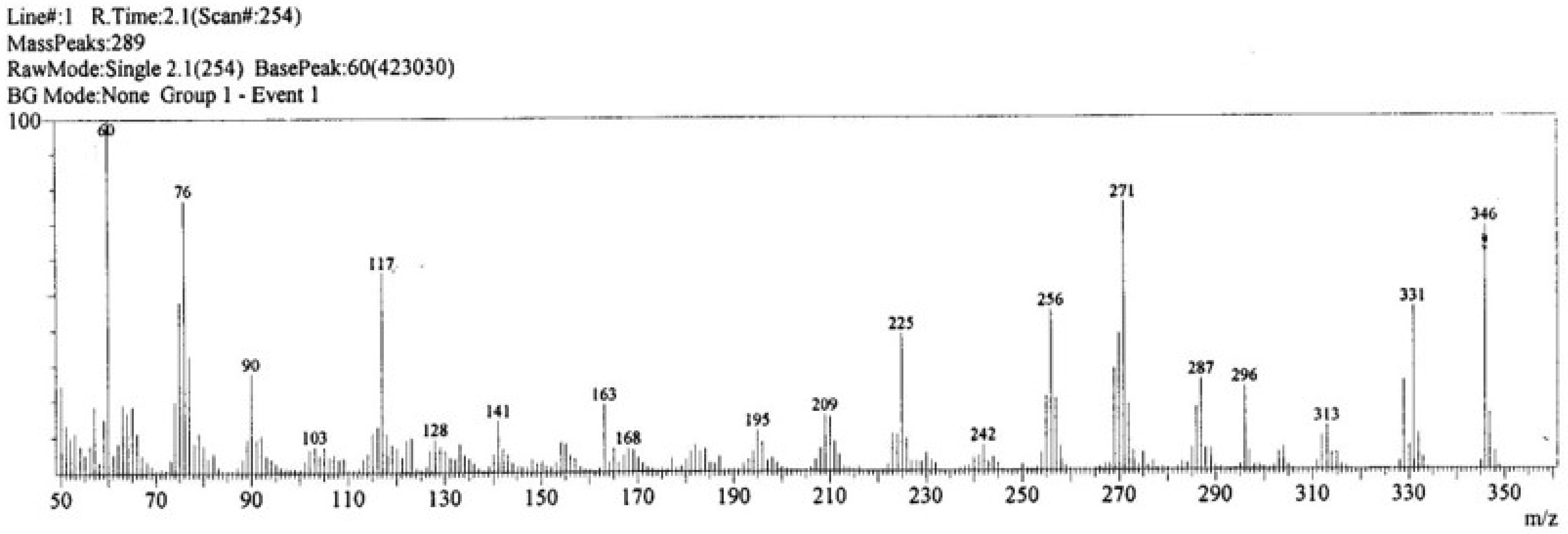

Mass Table
Line#:1 R.Time:2.1(Scan#:254)
MassPeaks:289
RawMode:Single 2.1(254) BasePeak:60(423030)
BG Mode:None Group 1 - Event 1

#	m/z	Abs. In	Rel. Int.	#	m/z	Abs. In	Rel. Int.	#	m/z	Abs. In	Rel. Int.
1	50.05	103272	24.41	4	53.05	47124	11.14	7	56.10	32281	7.63
2	51.05	56509	13.36	5	54.05	31940	7.55	8	57.05	78022	18.44
3	52.05	40823	9.65	6	55.10	21977	5.20	9	58.05	13451	3.18

Mass spectrometry of compound **14**.

Line#:1 R.Time:3.6(Scan#:429)
MassPeaks:332
RawMode:Single 3.6(429) BasePeak:91(452497)
BG Mode:None Group 1 - Event 1

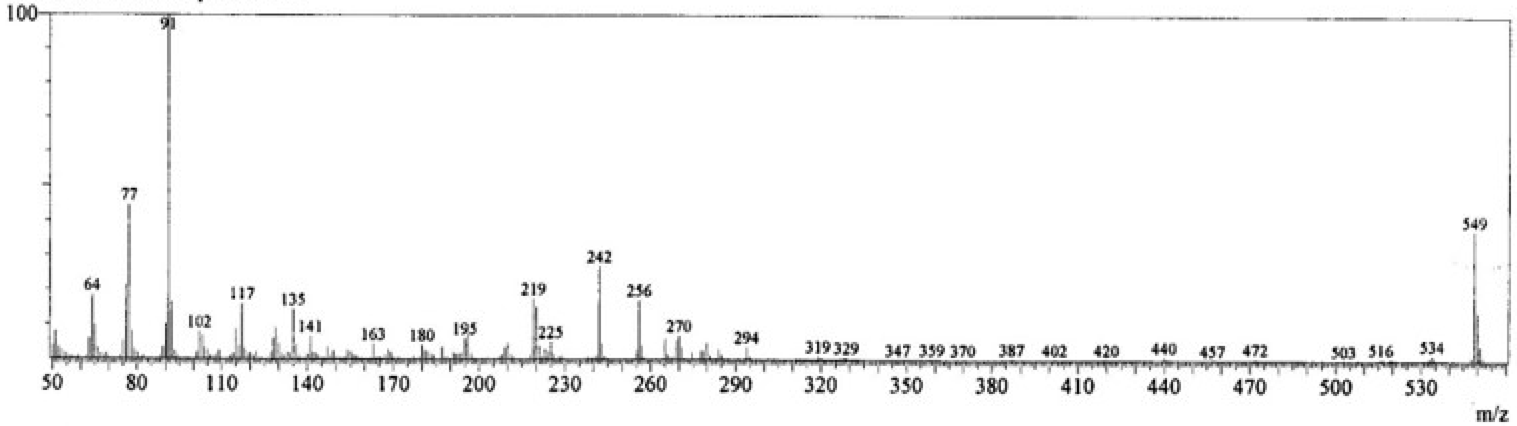

Mass Table
Line#:1 R.Time:3.6(Scan#:429)
MassPeaks:332
RawMode:Single 3.6(429) BasePeak:91(452497)
BG Mode:None Group 1 - Event 1

#	m/z	Abs. In	Rel. Int.	#	m/z	Abs. In	Rel. Int.	#	m/z	Abs. In	Rel. Int.
1	50.05	17536	3.88	4	53.05	11440	2.53	7	56.10	4516	1.00
2	51.05	35963	7.95	5	54.05	7970	1.76	8	57.05	3855	0.85
3	52.05	15270	3.37	6	55.05	6859	1.52	9	58.05	1760	0.39

Mass spectrometry of compound **17**.

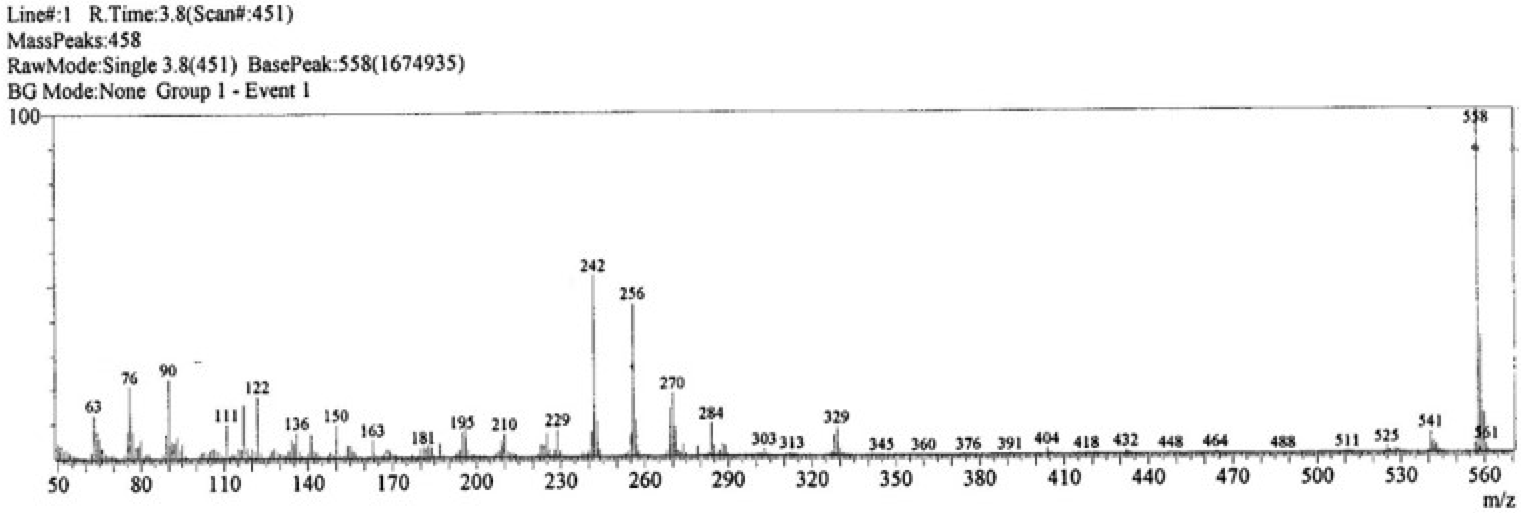

Mass Table
Line#:1 R.Time:3.8(Scan#:451)
MassPeaks:458
RawMode:Single 3.8(451) BasePeak:558(1674935)
BG Mode:None Group 1 - Event 1

#	m/z	Abs. In	Rel. Int.	#	m/z	Abs. In	Rel. Int.	#	m/z	Abs. In	Rel. Int.
1	50.05	73865	4.41	4	53.05	46038	2.75	7	56.05	14331	0.86
2	51.05	61739	3.69	5	54.05	28966	1.73	8	57.05	11224	0.67
3	52.05	40734	2.43	6	55.05	22633	1.35	9	58.00	7087	0.42

Mass spectrometry of compound **18**.

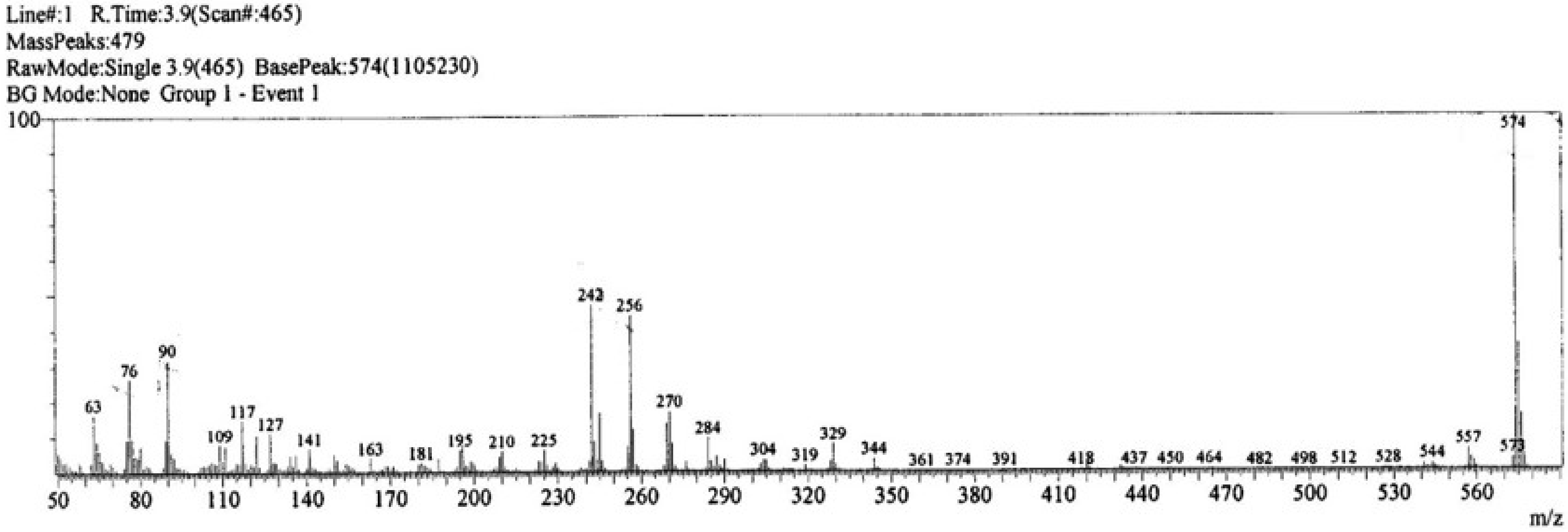

Mass Table
Line#:1 R.Time:3.9(Scan#:465)
MassPeaks:479
RawMode:Single 3.9(465) BasePeak:574(1105230)
BG Mode:None Group 1 - Event 1

#	m/z	Abs. In	Rel. Int.	#	m/z	Abs. In	Rel. Int.	#	m/z	Abs. In	Rel. Int.
1	50.05	60149	5.44	4	53.05	34412	3.11	7	56.10	10642	0.96
2	51.05	48788	4.41	5	54.10	23186	2.10	8	57.05	9888	0.89
3	52.05	34338	3.11	6	55.10	13017	1.18	9	58.00	33687	3.05

Mass spectrometry of compound **19**.

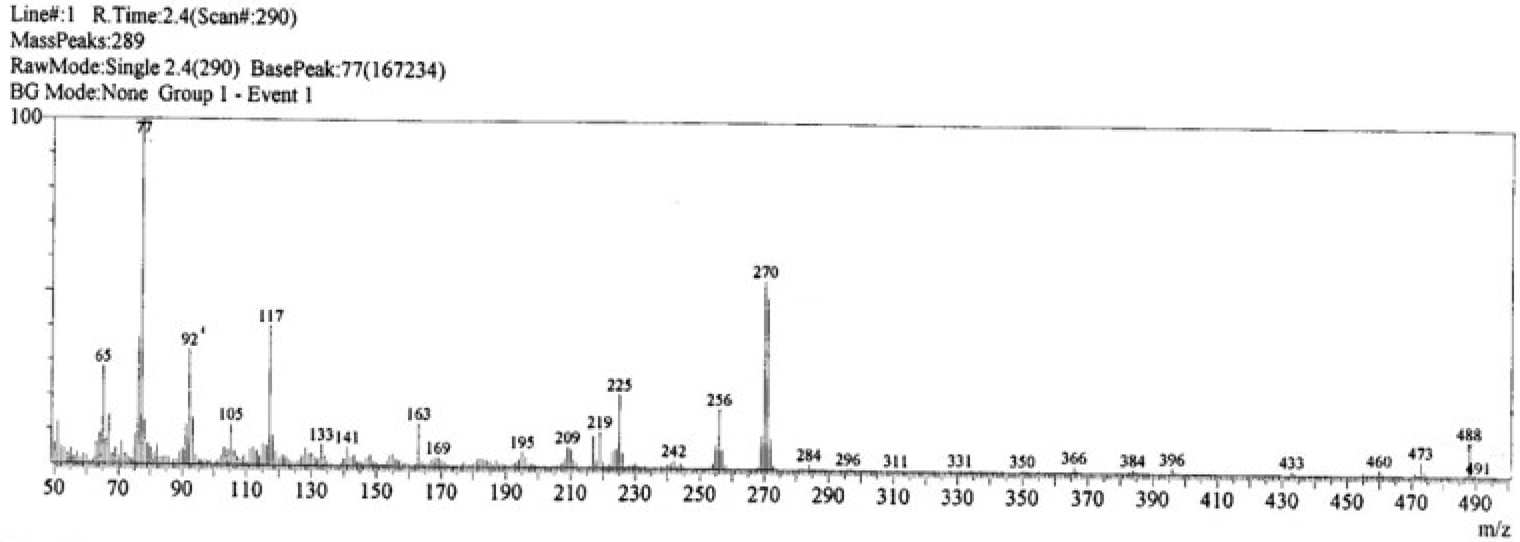

Mass Table
Line#:1 R.Time:2.4(Scan#:290)
MassPeaks:289
RawMode:Single 2.4(290) BasePeak:77(167234)
BG Mode:None Group 1 - Event 1

#	m/z	Abs. In	Rel. Int.	#	m/z	Abs. In	Rel. Int.	#	m/z	Abs. In	Rel. Int.
1	50.00	9914	5.93	4	53.05	7429	4.44	7	56.05	3812	2.28
2	51.00	20022	11.97	5	54.05	5722	3.42	8	57.05	5633	3.37
3	52.05	8420	5.03	6	55.05	7386	4.42	9	58.05	2613	1.56

Mass spectrometry of compound **23**.

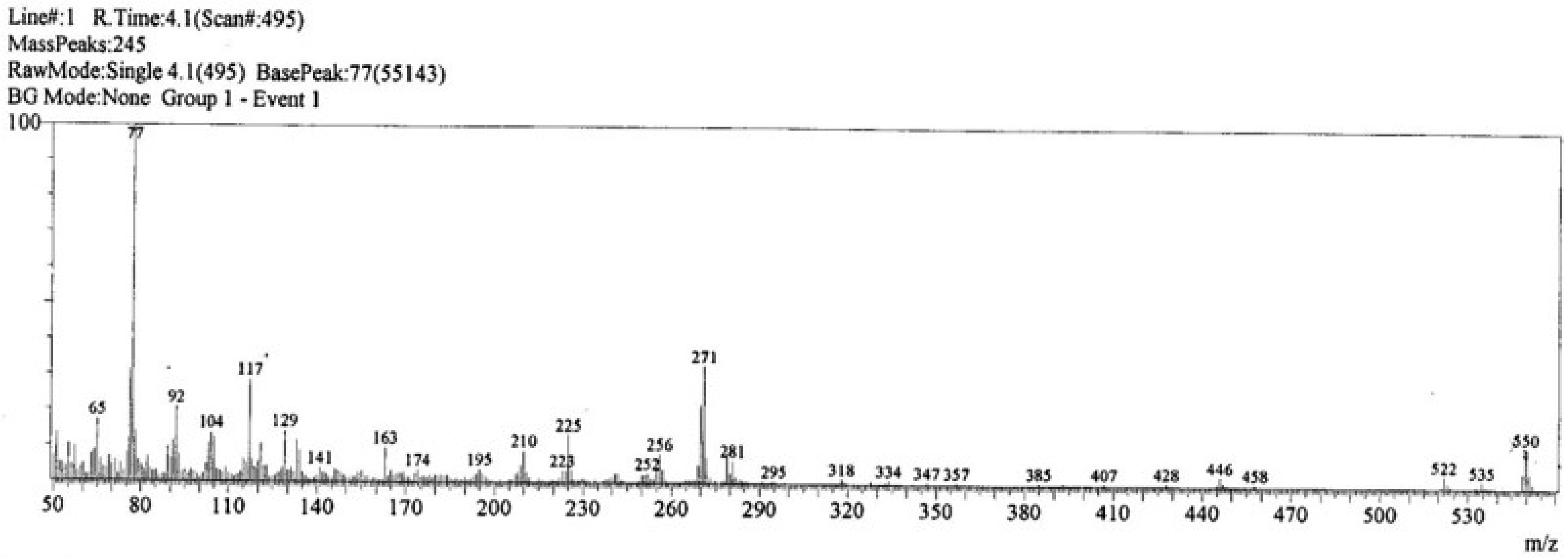

Mass Table
Line#:1 R.Time:4.1(Scan#:495)
MassPeaks:245
RawMode:Single 4.1(495) BasePeak:77(55143)
BG Mode:None Group 1 - Event 1

#	m/z	Abs. In	Rel. Int.	#	m/z	Abs. In	Rel. Int.	#	m/z	Abs. In	Rel. Int.
1	50.05	3937	7.14	4	53.05	2886	5.23	7	56.05	2674	4.85
2	51.00	7417	13.45	5	54.05	2471	4.48	8	57.05	5387	9.77
3	52.05	2978	5.40	6	55.05	5769	10.46	9	58.05	1615	2.93

Mass spectrometry of compound **24**.

Line#:1 R.Time:2.2(Scan#:268)
MassPeaks:218
RawMode:Single 2.2(268) BasePeak:77(35302)
BG Mode:None Group 1 - Event 1

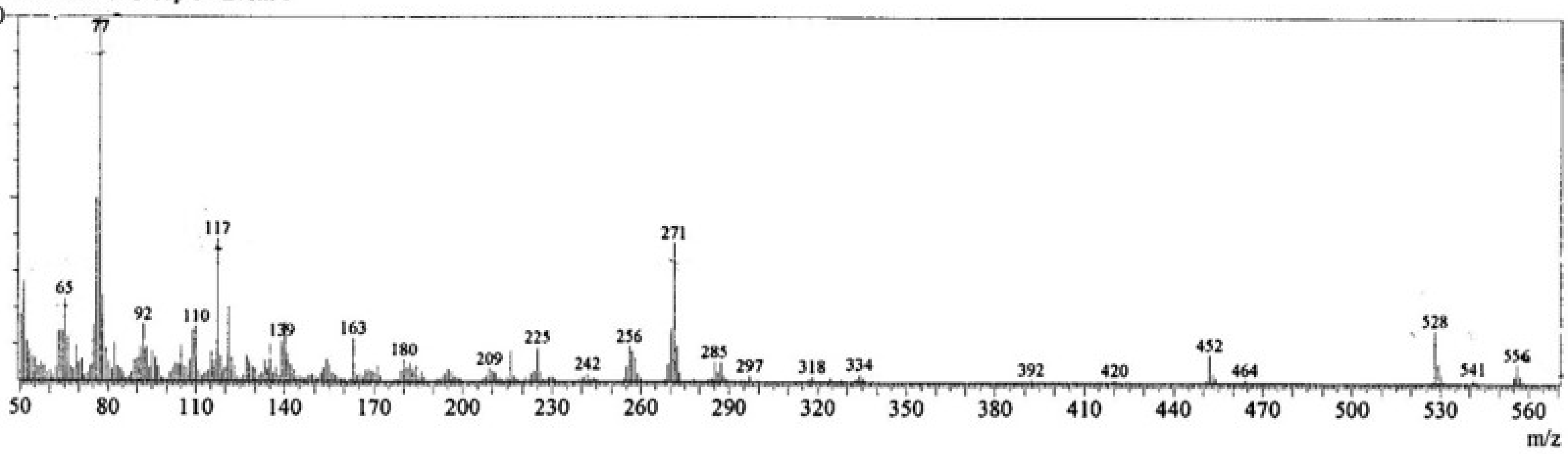

Mass Table
Line#:1 R.Time:2.2(Scan#:268)
MassPeaks:218
RawMode:Single 2.2(268) BasePeak:77(35302)
BG Mode:None Group 1 - Event 1

#	m/z	Abs. In	Rel. Int.	#	m/z	Abs. In	Rel. Int.	#	m/z	Abs. In	Rel. Int.
1	50.05	6456	18.29	4	53.05	2993	8.48	7	56.10	1398	3.96
2	51.05	9674	27.40	5	54.05	2328	6.59	8	57.05	1777	5.03
3	52.10	3879	10.99	6	55.05	2291	6.49	9	58.00	1623	4.60

Mass spectrometry of compound **26**.

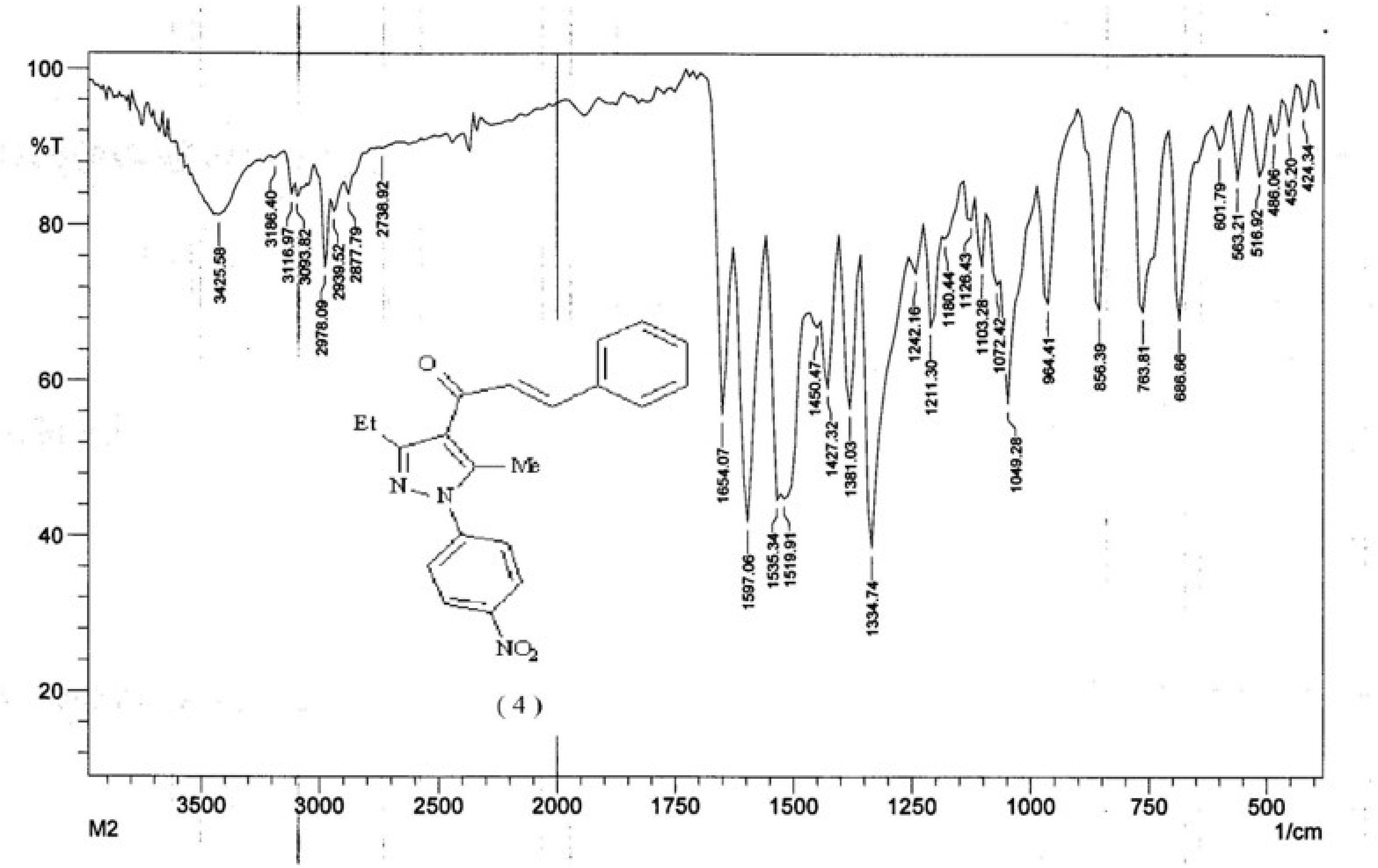

IR spectrum of compound **4**.

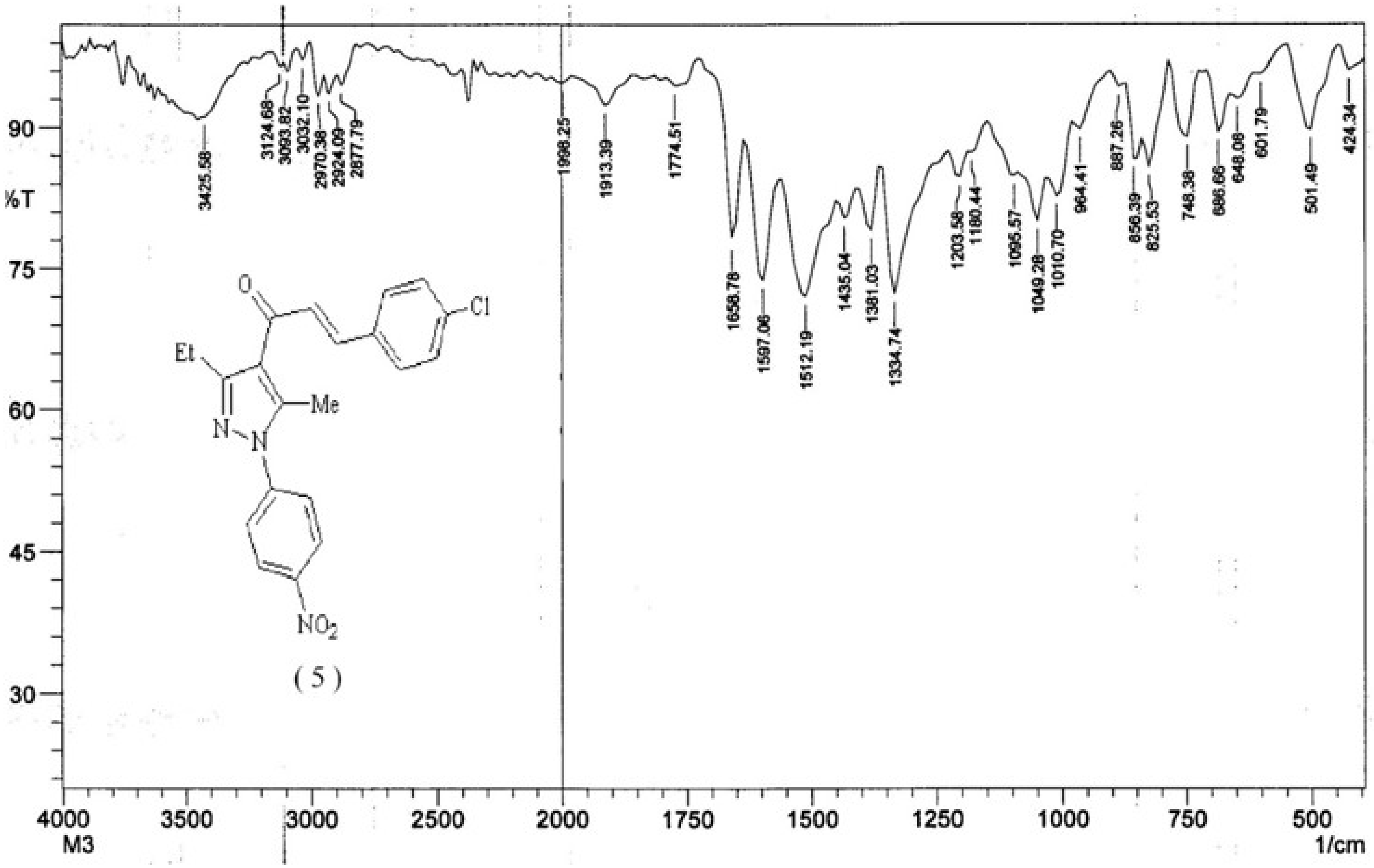

IR spectrum of compound **5**.

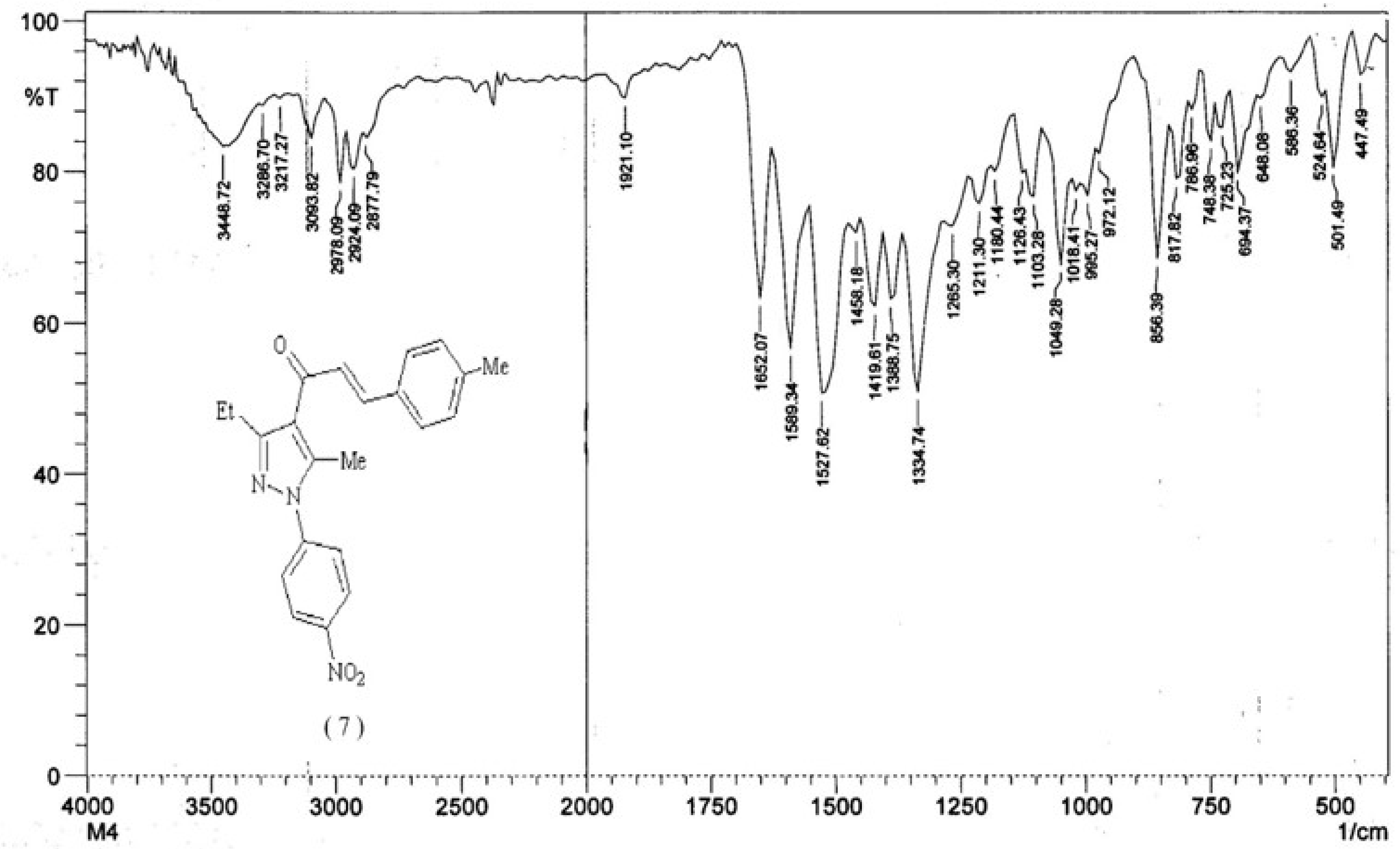

IR spectrum of compound **7**.

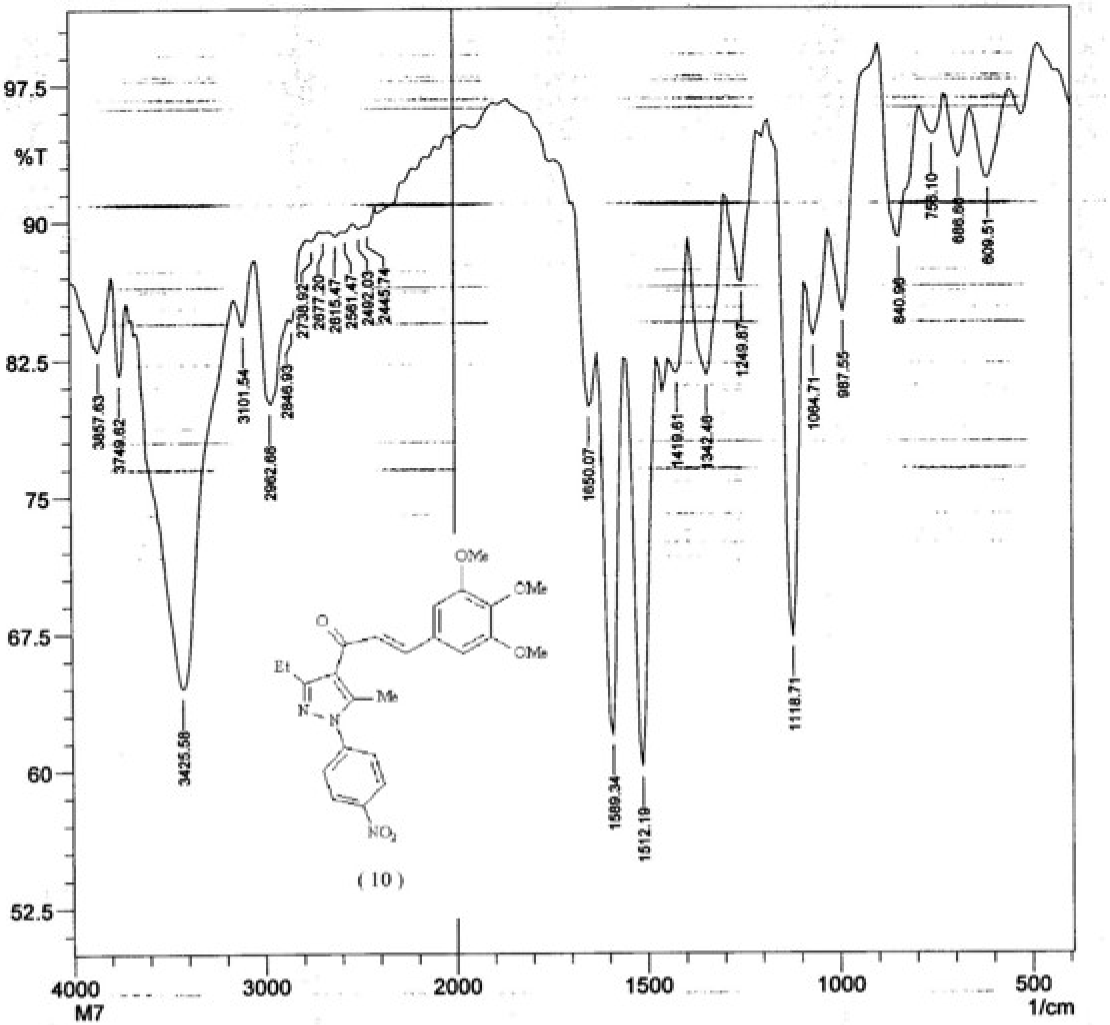

IR spectrum of compound **10**.

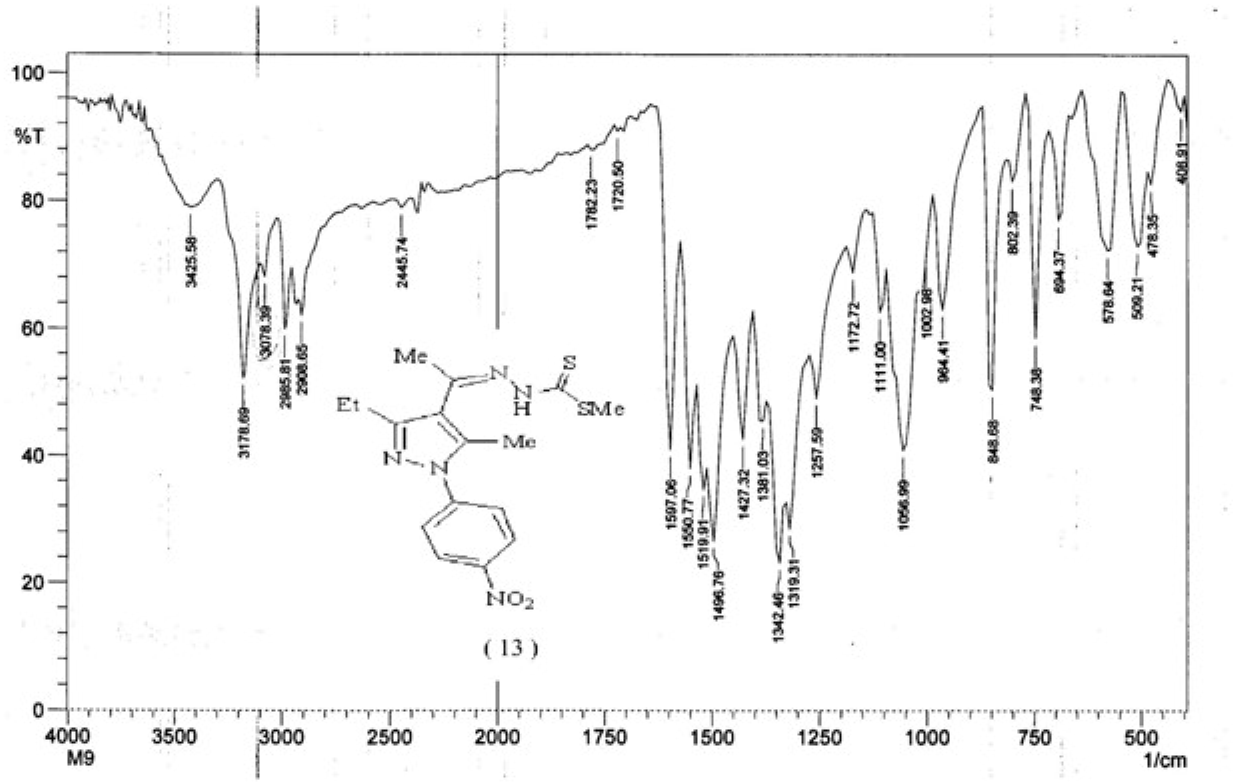

IR spectrum of compound **13**.

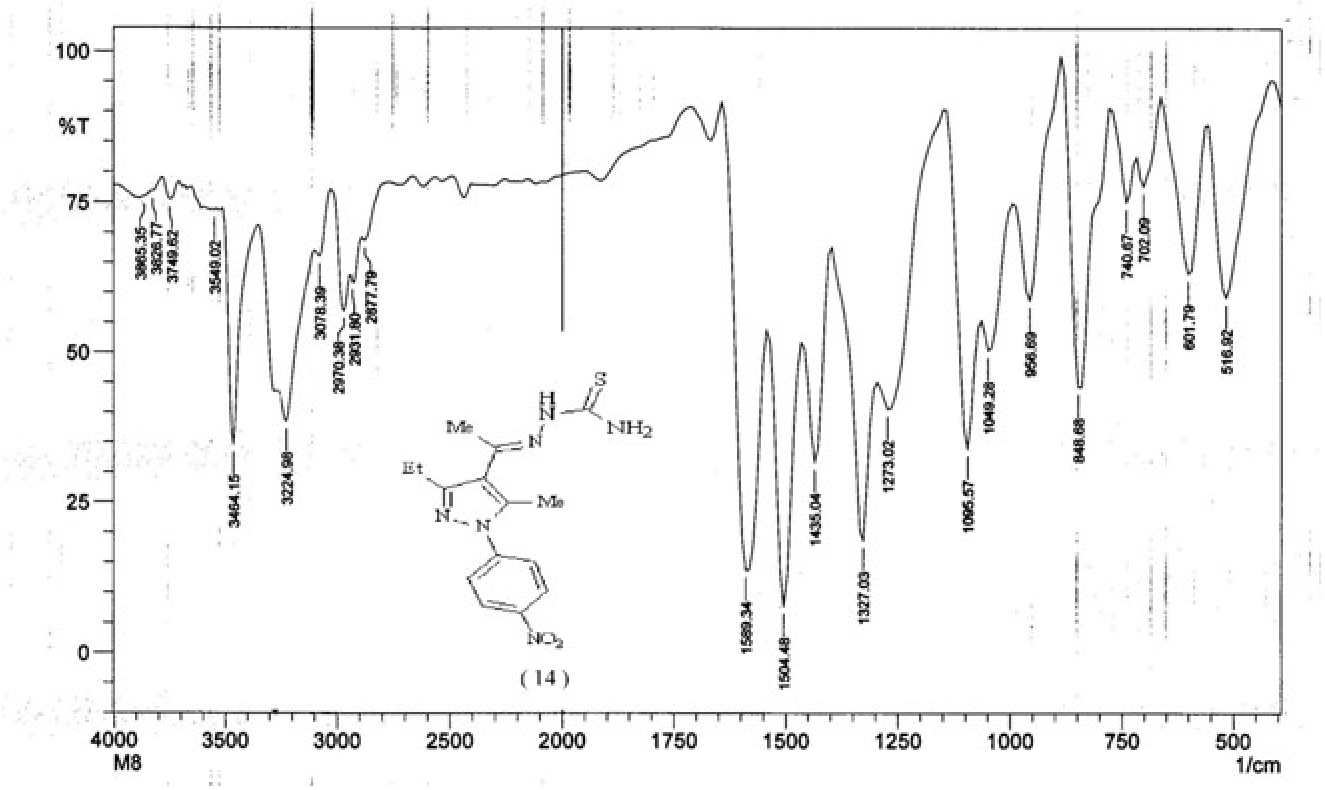

IR spectrum of compound **14**.

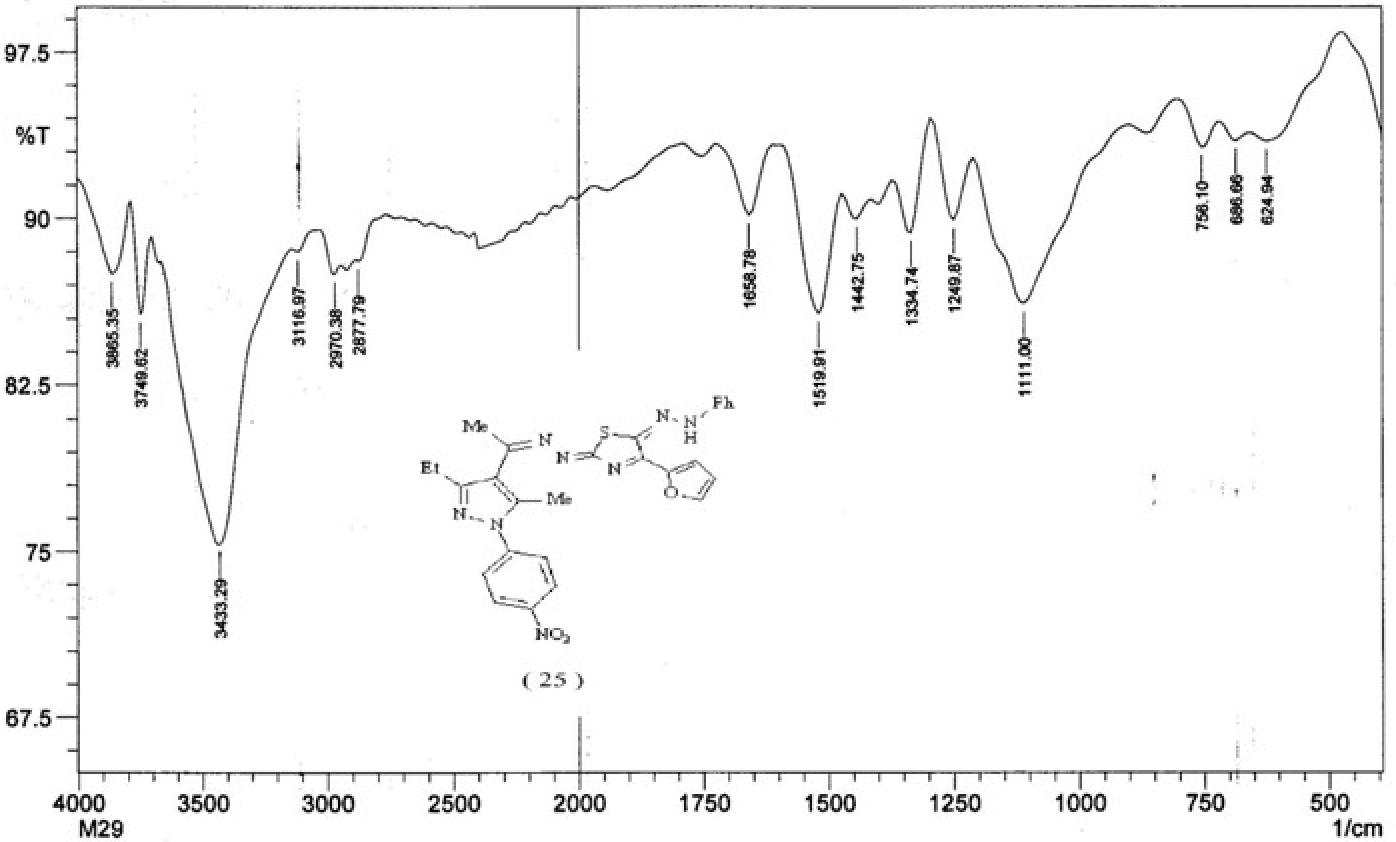

IR spectrum of compound **25**.
